# An early warning precision public health approach for assessing COVID-19 vulnerability in the UK: the Moore-Hill Vulnerability Index (MHVI)

**DOI:** 10.1186/s12889-023-17092-7

**Published:** 2023-11-02

**Authors:** Harriet Moore, Bartholomew Hill, Jay Emery, Mark Gussy, Aloysius Niroshan Siriwardena, Robert Spaight, Frank Tanser

**Affiliations:** 1 Department of Geography, University of Lincoln, Lincoln, United Kingdom; 2 Development, Inequalities, Resilience and Environments Research Group, Lincoln, United Kingdom; 3 EDGE Consortium, Lincoln, Ontario, United Kingdom, Canada; 4 WATERWISER/WEDC, Loughborough University, Loughborough, United Kingdom; 5 Lincoln International Institute for Rural Health, Lincoln, United Kingdom; 6 Community and Health Research Unit, School of Health and Social Care, University of Lincoln, Lincoln, United Kingdom; 7 East Midlands Ambulance Service NHS Trust, Nottingham, England; 8 School for Data Science and Computational Thinking, Stellenbosch University, Stellenbosch, South Africa

**Keywords:** COVID-19, Vulnerability index, Precision public health, Ambulance data

## Abstract

**Background:**

Most COVID-19 vulnerability indices rely on measures that are biased by rates of exposure or are retrospective like mortality rates that offer little opportunity for intervention. The Moore-Hill Vulnerability Index (MHVI) is a precision public health early warning alternative to traditional infection fatality rates that presents avenues for mortality prevention.

**Methods:**

We produced an infection-severity vulnerability index by calculating the proportion of all recorded positive cases that were severe and attended by ambulances at small area scale for the East Midlands of the UK between May 2020 and April 2022. We produced maps identifying regions with high and low vulnerability, investigated the accuracy of the index over shorter and longer time periods, and explored the utility of the MHVI compared to other common proxy measures and indices. Analysis included exploring the correlation between our novel index and the Index of Multiple Deprivation (IMD).

**Results:**

The MHVI captures geospatial dynamics that single metrics alone often overlook, including the compound health challenges associated with disadvantaged and declining coastal towns inhabited by communities with post-industrial health legacies. A moderate negative correlation between MHVI and IMD reflects spatial analysis which suggests that high vulnerability occurs in affluent rural as well as deprived coastal and urban communities. Further, the MHVI estimates of severity rates are comparable to infection fatality rates for COVID-19.

**Conclusions:**

The MHVI identifies regions with known high rates of poor health outcomes prior to the pandemic that case rates or mortality rates alone fail to identify. Pre-hospital early warning measures could be utilised to prevent mortality during a novel pandemic.

**Supplementary Information:**

The online version contains supplementary material available at 10.1186/s12889-023-17092-7.

## Introduction

The COVID-19 pandemic has illuminated persistent health inequalities globally, within countries, and between regions [1]. The impact of the virus on human health [2], and economic security [3], has not been homogenous. In the context of financial constraint, governments have struggled to prioritise the allocation of scarce resources to address the most immediate challenges of the pandemic, including mitigating transmission, and protecting vulnerable communities from severe illness and mortality. To this end, numerous efforts have been made during the pandemic and retrospectively in consideration of future pandemics to develop vulnerability indices for informing mitigation measures, such as localised lockdowns and the dissemination of personal protective equipment (PPE) to healthcare workers [4]. Here, we present the Moore-Hill Vulnerability Index (MHVI) which represents the proportion of all positive cases that are likely to have been *severe* in the East Midlands of the UK at a small area scale known as Middle Super Output Area (MSOA: average of 7200 people). Our approach utilizes spatial records of ambulance attendance as a proxy for severe illness. In this context, vulnerability refers to the intersection of both *exposure* and *susceptibility to sever illness* [5]; similar to traditional infection-fatality rate analysis, our proposed index suggests that regions with higher proportions of severe cases compared to instances of infection are more ‘vulnerable’ than those with lower proportions of severe cases. To our knowledge, the MHVI is the first index to operationalise prehospital data for precision public health and as an early warning system during a novel pandemic.

Over the past two years, multiple publications have presented vulnerability indices for COVID-19. Of these, most vulnerability indices fall into three categories. The first category is indices that use pre-existing metrics, such as regional rates of severe respiratory illness prior to the current pandemic [6, 7] to *estimate* areas with communities that are likely to be particularly vulnerable to illness from COVID-19 [8–12]. The second category is indices that utilise case rates or estimates of basic reproductive numbers (R0) [13] as measures of vulnerability, including developing predictive models. Multiple indices aggregate pre-existing metrics [14–18], like socio-economic [19, 20], demographic [21], landscape [22], and health measures [4, 23, 24] to predict positive case rates.

Case-rates are a more reliable indicator of vulnerability when the physiological impact of a virus is relatively homogenous and infection is likely to result in severe illness or mortality, such as with cholera in the 1800s [25] or more recently in Africa [26]. Historically, without treatment the proportion of all positive cholera cases resulting in mortality has been high [27, 26]. Thus, in many regions of the world, community case-rates are a useful metric for identifying vulnerable areas and prioritising investment. By comparison to cholera, a much lower proportion of individuals who contract COVID-19 experience severe or fatal symptoms [28], although the likelihood of mortality varies markedly between demographic groups, regions [29], and individuals with prior health conditions [30]. Thus, like influenza, positive case-rates are not a reliable indicator for identifying particularly vulnerable communities or regions for the purpose of mitigation.

The third category of vulnerability indices uses existing metrics similar to those described above (e.g., socio-economic measures) to predict cases of mortality [31–34]. Mortality reflects vulnerability, however, without consideration of unequal rates of exposure. Two communities with similar underlying vulnerabilities to severe illness from COVID-19 may experience vastly different mortality rates per population due to varying exposure. For example, in the UK aging communities in rural areas who are typically more vulnerable to severe illness from COVID-19 tend to be spatially isolated from younger communities in urban areas [35]. In the early days of the pandemic these vulnerable rural communities were relatively spatially segregated from high-density urban areas with high case-rates. As the pandemic progressed, some areas experienced increased exposure due to internal migration [36], including the mobility between urban and rural areas as urban residents relocated to second homes in rural areas [37], and for holidays during summer months [38]. Thus, the likelihood of exposure, and in turn mortality, increased in popular, previously isolated locations. For this reason, mortality rates alone may underrepresent vulnerability in more spatially isolated rural areas; vulnerability is the product of both physical exposure, which is likely to vary over time in the context of evolving mitigation policy, and underlying susceptibility.

The most reliable measure of vulnerability considers the relationship between all positive cases and severe cases, such as infection fatality rate (IFR) [39, 40] or case fatality rate (CFR) [41, 42]. Both IFR and CFR reflect the proportion of positive cases that result in mortality. These measures are more accurate than either positive case-rates or mortality rates alone because vulnerability is the product of both physical exposure, reflected in case rates, and underlying susceptibility, reflected in mortality rates. Thus, geospatial comparison between regions to identify priority areas for investment and localised lockdowns should consider the ratio of all cases that are likely to be severe to control for exposure rates. Here, we present the MHVI which captures exposure and underlying susceptibility by computing the proportion of all positive cases that required ambulance attendance as an early-warning proxy measure of severe illness or future mortality.

Ambulance attendance for suspected severe COVID-19 reflects the intersectionality of exposure to the virus and underlying susceptibility, often related to pre-existing comorbidities and deprivation [5]. Unlike mortality rates, ambulance attendance records afford an opportunity for intervention in advance of illness escalating to fatality. For example, Fitzpatrick et al. [43] conducted data linkage in Scotland and explored patient pathways from ambulance attendance through to successive interactions with health care services. They found that ambulance attendance for patients with suspected COVID-19 that did not result in conveyance to hospital because symptoms were not deemed to be sufficiently acute was a strong predictor of mortality from COVID-19 within a 30-day period. Further, for 50% of 5,720 patients attended by ambulances for COVID-19, the next unscheduled health appointment was presentation to emergency departments for severe COVID-19. Thus, ambulance attendance can act as an early warning for fatality and vulnerability to severe illness, as well as for tracking the geospatial progression of contagion in the early days of a novel pandemic before widespread community testing [5].

Records of ambulance attendance may inform a suitable metric for precision public health because during a pandemic with successive periods of lockdown when mobility is restricted, medical emergencies occur within homes, thus indicating vulnerable communities and regions. Our index computes the proportion of all confirmed positive cases that are likely to have been severe by comparing the number of positive cases reported by GOV.UK to the number of ambulance attendances for suspected severe COVID-19 in the East Midlands of the UK, between May 18th, 2020, when widespread community testing began, and April 2nd, 2022. Prior to widespread community testing, records of positive case-rates were not a reliable reflection of cases in communities. The MHVI is reported as a ratio from 0 to 1 whereby 0 reflects least vulnerability to severe illness and 1 reflects greatest vulnerability. These MHVI scores are computed and visually displayed using mapping at the MSOA scale. Here we present our findings about most vulnerable regions in the East Midlands of the UK, demonstrate the utility of the MHVI as an infection-severity precision public health early warning system, and suggest some avenues for reducing risk of mortality in the early phase of a novel pandemic.

## Methods

### Site location and population

The study region was the East Midlands, which represents a microcosm of the wider UK in relation to ethnic diversity, urban-rural characteristics, and socio-economic dynamics [44]. The East Midlands spans an area of 15,627km [2], with an estimated population of 4.8 million, and lies in the Central Eastern part of England, including the urban areas of Derby, Leicester, Lincoln, Nottingham and Northampton. The population included, (a) all patients attended by ambulance for suspected severe illness from COVID-19 following 999 calls to the East Midlands Ambulance Service NHS Trust (EMAS) between May 18th, 2020, and April 2nd, 2022, and (b) all patients who tested positive with COVID-19 determined over the same period, as determined by laboratory testing and reported by the UK Health Security Agency, via the GOV.UK data request dashboard [45].

### Study design

The research employed a cross-sectional design using retrospective routine data collated by EMAS, and open-source records of weekly confirmed COVID-19 cases occurring at the scale of MSOA reported by GOV.UK. The EMAS dataset contained all records of ambulance attendance for suspected severe illness from COVID-19, including partial postcodes for each record. Postcodes were aggregated to the scale of MSOA using ArcGISPro^TIM^. Thus, both datasets involved in the research contained records of numbers of patients with suspected or confirmed COVID-19 as well as a common geospatial reference, the MSOA.

### Research aims

The first aim of the research was to develop, and visually display with mapping an early warning precision public health vulnerability index, which we have termed the MHVI, using a similar approach to computing IFR and CFR. This was achieved by comparing case records of ambulance attendance for suspected severe illness from COVID-19 with records of all positive cases for the East Midlands at the scale of Middle Super Output Area (MSOA). The analysis was conducted for all data recorded between 18th May 2020 and 2nd April, 2022, as well as for three cumulative intervals: 18th May, 2020 to October 31st (approximately 5 months), 18th May, 2020 to 17th April, 2021 (12 months), and 18th May, 2020 to October 2nd, 2021 (approximately 17 months). These dates were chosen in accordance with the weekly cumulative data available from GOV.UK, and to ensure that each time period included the addition of a similar range of new data. This approach was taken to explore the utility of the MHVI in the real-world context of a newly emerging infectious disease and evolving datasets. The purpose of conducting the analysis at these cumulative intervals was partly methodological, and twofold; to consider changing spatial patterns of vulnerability over the course of the pandemic, and to determine an approximate timeframe for the MHVI stabilising to reflect the geospatial patterns observed from analysing the full dataset.

The second aim of the research was to explore the utility of the MHVI for identifying vulnerable regions. This was achieved by comparing the spatial characteristics of the MHVI with the characteristics of case-rate data and with a validated vulnerability metric; the Index of Multiple Deprivation (IMD). Maps were prepared to visually display case-rate data and the IMD for the East Midlands region. Our expectation was that vulnerable regions identified by the MHVI would correspond with some vulnerable regions highlighted by case-rates and identify some additional regions that case-rates alone may obscure. Deprivation is one of the strongest predictors of vulnerability to severe illness from COVID-19 [46, 47]. Thus, if reliable, we expected considerable overlap between the MHVI and the IMD, with the MHVI identifying additional regions, such as affluent aging communities in rural areas. In addition to visual analysis, we computed a Pearson’s correlation coefficient to determine the strength and direction of relationship between the MHVI and IMD decile scores. These analyses were undertaken to consider the validity of our new metric.

### Measures

Three measures were included in the research: positive cases recorded by GOV.UK and obtained via an online data request dashboard developed by the UK Health Security Agency (GOV.UK, 2022), records of patients attended by the East Midlands Ambulance NHS Trust (EMAS) for suspected severe illness from COVID-19, and the Index of Multiple Deprivation (IMD) scores for the study region. The IMD is an aggregate index that synthesises values from nine domains, including income, employment and health measures [48]. This research utilises decile IMD values rather than raw scores. Table 1 summarises these data and data sources.

**Table 1:** Measures included in the research

Measure	Data	Source
IMD	Decile values (1 = most deprived, 10 = most affluent)	https://hub.arcgis.com/datasets/communities:lower-super-output-area-lsoa-imd-2019-osgb1936
Positive cases	Weekly sum of cases per MSOA	https://coronavirus.data.gov.uk/details/download
Severe suspected cases	Daily sum of ambulance attendance	EMAS

Records of ambulance attendance for suspected severe illness from COVID-19 were collated and obtained from EMAS, including provisional diagnosis by medically trained clinicians. In the absence of laboratory testing, these clinical assessments, including biological measures like blood-oxygen levels, have been shown to be reliable indicators of COVID-19 positive patients [5, 49]. Before laboratory capacity for community testing increased in May of 2020, testing by polymerase chain reaction (PCR) was largely confined to patients suspected of severe illness, including those conveyed by ambulance to hospitals. Prior research has found a very strong positive correlation between daily rates of suspected severe illness from COVID-19 based on ambulance attendance records, and daily rates of cases confirmed by PCR in the early months prior to May 18th when widespread community testing began [5]. After this point in time, the number of confirmed cases far exceed the number of ambulance attendance for suspected severe illness from COVID-19. Confirmed cases reflect risk of exposure while severe illness, such as cases attended by ambulances, indicate the intersectionality of exposure *and* underlying susceptibility, including old age and prior health conditions.

### Data cleaning and handling

**Fig. 1:**
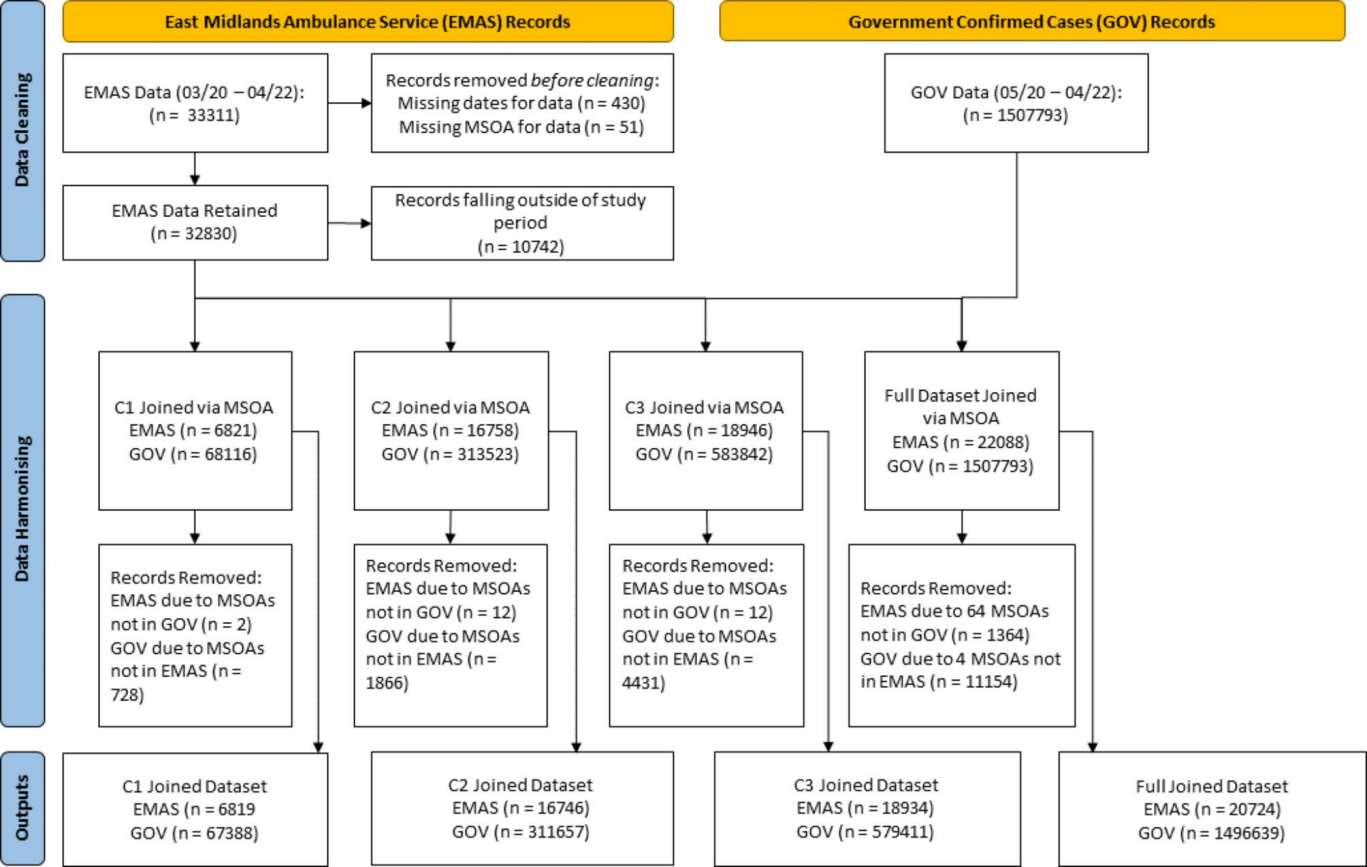
Schematic of data harmonisation steps for East Midlands Ambulance Service NHS Trust (EMAS) records of suspected severe illness from COVID-19 and GOV.UK records of cases confirmed by laboratory testing. Harmonisation steps are included for the full dataset and three cumulative periods, C1, C2 and C3

We linked one socio-economic dataset and two clinical datasets. For the purpose of comparative analysis with the MHVI, Index of Multiple Deprivation decile scores were aggregated from lower super output area (LSOA) to MSOA. Clinical datasets included daily records of ambulance attendance for suspected severe COVID-19 in the East Midlands area, and weekly aggregate records of COVID-19 cases confirmed cases by laboratory tests, over a seven-day period, from the date the sample was taken of the person being tested. Two stages of data cleaning were undertaken over a two-day period. Firstly, cleaning was conducted to harmonise the daily ambulance records with weekly GOV.UK records of confirmed cases at the scale of MSOA to produce the final data set for computing the MHVI for the period May 18th, 2020 to April 2nd, 2022. Secondly, this process was repeated on sub-sets of the data to investigate changing geospatial trends over time, and to identify a reliable time-period for analysing vulnerability. Three cumulative sub-sets were produced at approximately 5-month intervals (0-5months, 0–11 months, and 0–17 months). Additional cleaning was also required to harmonize ambulance and GOV.UK records over these time periods. Data harmonising steps are detailed in S-3 and visualised in Fig. 1.

### Construction and spatial representation of the Moore-Hill Vulnerability Index (MHVI)

Following data harmonisation, the MHVI was calculated for the full dataset (May 18th, 2020, to April 2nd, 2022) as well as for the three cumulative periods specified above (C1, C2 and C3). MHVI scores compare the number of ambulance attendances for suspected severe illness from COVID-19 to the overall number of positive cases proportionally, at the scale of MSOA. A score closer to the value of 1 indicates that a high proportion of all positive cases were likely to have been attended by ambulances and considered severe. A score closer to the value of 0 indicates that a low proportion of all positive cases were likely to have been attended by ambulances. These scores were displayed visually on four maps, one each for the three cumulative periods considered, and a fourth displaying scores computed from the full dataset. For the purpose of mapping, scores were divided into class intervals using the geometric intervals classification (GIC). This algorithm takes into consideration equal interval, natural breaks [50], and quantile methods, to balance and highlight both middle and extreme values within classifications [51]. This approach minimizes the sum of squares of the number of elements in each interval class. Given that the rates of positive and severe cases of COVID-19 varied over time, the range of scores also varied per cumulative period. Thus, rather than using standardised intervals for all maps, intervals were computed for each cumulative period. Interval values are displayed on maps to demonstrate how proportions of overall cases that are likely to have been severe changed throughout the pandemic.

## Results

### Descriptive statistics

Table 2 reports descriptive statistics, including the number of records of patients attended by ambulances for suspected severe COVID-19 (EMAS data) and the number of records of confirmed cases (GOV.UK). These statistics are reported for the full study period (Full) between May 18th, 2020, and April 2nd, 2022, as well as for three cumulative periods: cumulative period one (C1) is between May 18th, 2020, and October 31st, 2020; cumulative period two (C2) is between May 18th, 2020, and April 17th, 2021; cumulative period three (C3) is between May 18th, 2020, and October 2nd, 2021. These statistics reflect the data included in the research after data cleaning.

**Table 2:** Descriptive statistics for the number of records of patients attended by ambulances for suspected severe COVID-19 by the East Midlands Ambulance Service NHS Trust (EMAS), the number of records of confirmed cases by GOV.UK, and the number MSOAs with both ambulance attendance and GOV.UK records

Time period	EMAS suspected cases	GOV.UK confirmed cases	Number of MSOAs
C1	6819	67,388	568
C2	16,758	311,657	569
C3	18,934	579,411	569
Full	20,724	1,496,639	564

### The Moore-Hill Vulnerability Index scores

The first aim of the study was to develop and display the MHVI. The MHVI was determined by computing the proportion of COVID-19 cases confirmed and reported by GOV.UK that were likely to have been severe based on ambulance attendance for suspect severe COVID-19. This analysis was conducted for the full dataset (May 18th, 2020, to April 2nd, 2022) as well as for the three cumulative periods defined above. The MHVI was computed at the scale of MSOA. MHVI scores are values between 0 (representing low vulnerability) and 1 (representing high vulnerability). These scores are presented in S-2 (Table S5-S8). A summary of the 20 maximum and minimum values for the full dataset, including number of confirmed cases (GOV.UK), number of suspected severe illness (EMAS), and location is presented in Table 3 below. For the purpose of ground-truthing these observations we have also included aggregate IMD decile scores for each of the most and least vulnerable MSOAs.

**Table 3:** Maximum and minimum values for the full dataset, including MHVI, records of positive cases of COVID-19 (GOV.UK), records of cases attended by ambulance for suspected severe illness from COVID-19 (EMAS), and location (MSOA). The top twenty maximum MHVI values and records, representing greatest vulnerability, are represented in black. These values are listed in order from most vulnerable regions. The bottom twenty minimum MHVI values and records, representing least vulnerability, are represented in grey. These values are listed in order from least vulnerable regions

MHVI	GOV.UK cases	EMAS cases	MSOA	Area name	IMD
0.0616	1640	101	E02005433	Ingoldmells & Chapel St Leonards	1
0.0437	1098	47	E02005429	Sutton-on-Sea	2
0.0405	1826	73	E02005428	Mablethorpe	1
0.0331	2176	72	E02002884	Forest Fields	2
0.0314	2069	65	E02005438	Skegness South	1
0.0301	2160	65	E02002887	Beechdale	1
0.0291	2679	78	E02004116	Clay Cross	3
0.0276	3152	87	E02002813	Rose Hill & Castleward	1
0.0265	1846	49	E02005437	Skegness Town	1
0.0265	2188	57	E02004102	Buxton North	5
0.0259	2205	57	E02005846	Worksop Cheapside	1
0.0253	1892	47	E02004034	Ripley West	8
0.0252	3806	94	E02002898	Lenton & Dunkirk	6
0.0252	2102	53	E02005467	Holbeach	6
0.0250	1797	43	E02004067	Boythorpe & Birdholme	1
0.0249	2121	53	E02005664	Kingsley Park & Racecourse	3
0.0248	2302	57	E02002904	Clifton South	2
0.0244	2369	58	E02002831	Rushey Mead South	4
0.0241	3323	80	E02002845	North Evington & Rowlatts Hill	2
0.0240	2331	56	E02005673	Stornton & Sixfields	4
0.0032	2843	9	E02005630	Oundle, Warmington & Titchmarsh	9
0.0040	3221	13	E02005390	Burbage Sketchley & Stretton	10
0.0046	2394	11	E02004088	Long Eaton West	10
0.0046	2166	10	E02005919	Ratcliffe, Sutton Bonington & Gotham	9
0.0048	2523	12	E02005380	Market Bosworth, Barlestone & Sheepy Magna	9
0.0050	2622	13	E02005343	Countesthorpe & Kilby	10
0.0050	2773	14	E02005372	Dunton Bassett, Claybrooke & Swinford	9
0.0051	2559	13	E02005920	East Leake	10
0.0052	2115	11	E02004122	Willington South & Repton	10
0.0053	3000	16	E02005381	Desford & Newbold Verdon	8
0.0057	2479	13	E02005865	Ravenshead & Newstead	9
0.0057	3004	15	E02005909	Lady Bay (urban minor con)****	10
0.0058	2739	16	E02005499	Dunholme & Welton	8
0.0059	3207	19	E02005387	Hinckley West (urban city town)****	7
0.0061	2311	14	E02002867	Uppingham, Lyddington & Braunston	9
0.0061	2950	18	E02005622	Long Buckby East & Ravensthorpe	10
0.0061	1471	9	E02005918	Keyworth South	9
0.0061	3104	19	E02005370	Broughton Astley	10
0.0065	1841	12	E02005460	Ruskington West & Cranwell	10
0.0066	2725	18	E02005399	Ashby de la Zouch North (urban city town)***	8

### Visual analysis

**Fig. 2:**
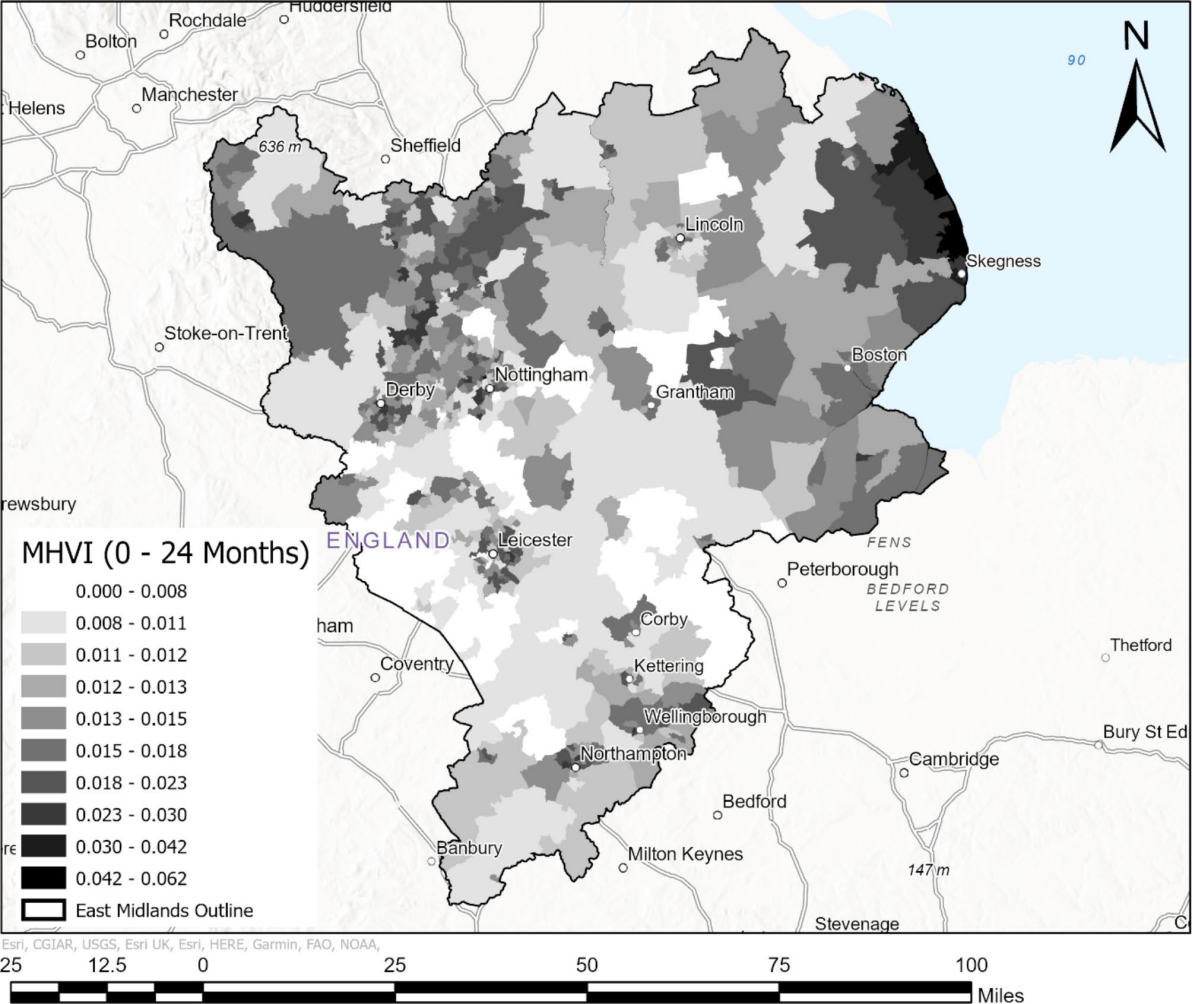
Map displaying MHVI scores for the East Midlands of the UK at the scale of MSOA. Scores were calculated for the entire study period, between May 18th, 2020, and April 2nd, 2022

**Fig. 3:**
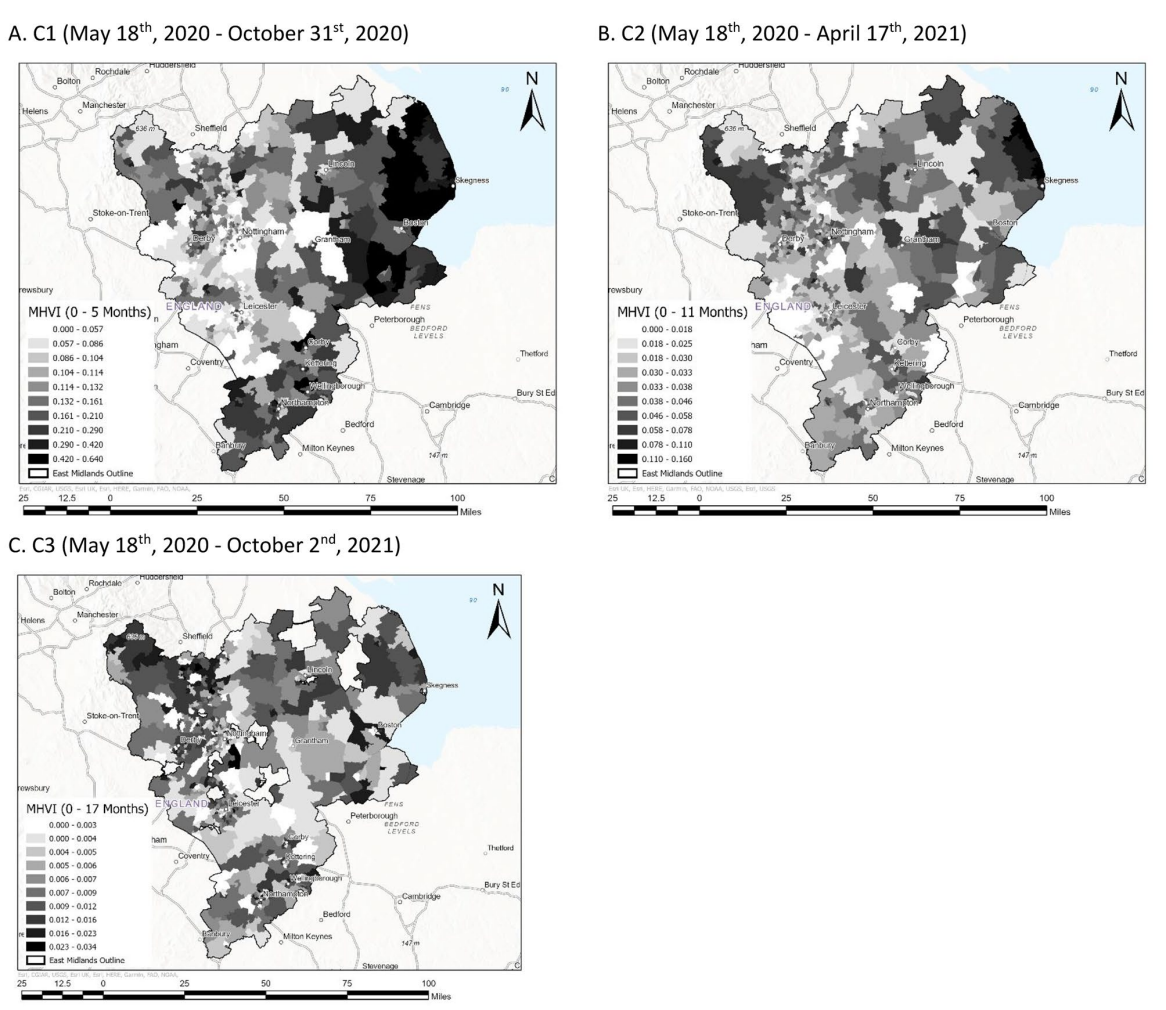
Map displaying MHVI scores for the East Midlands of the UK at the scale of MSOA. Scores were calculated for each of three data sub-sets reflecting the three cumulative periods considered for the purpose of comparative analysis (C1, C2 and C3)

Maps were produced to visually present the MHVI at the scale of MSOA for the full study period (May 18th, 2020, to April 2nd, 2022) as well as for each of the cumulative periods defined above (C1, C2 and C3). Fig. 2 displays the MHVI mapped at MSOA for the full study period. Fig. 3 displays the MHVI mapped at MSOA for each of the cumulative periods C1 (map a), C2 (map b), and C3 (map c).

### Exploring the utility of the MHVI for identifying vulnerable regions

**Fig. 4:**
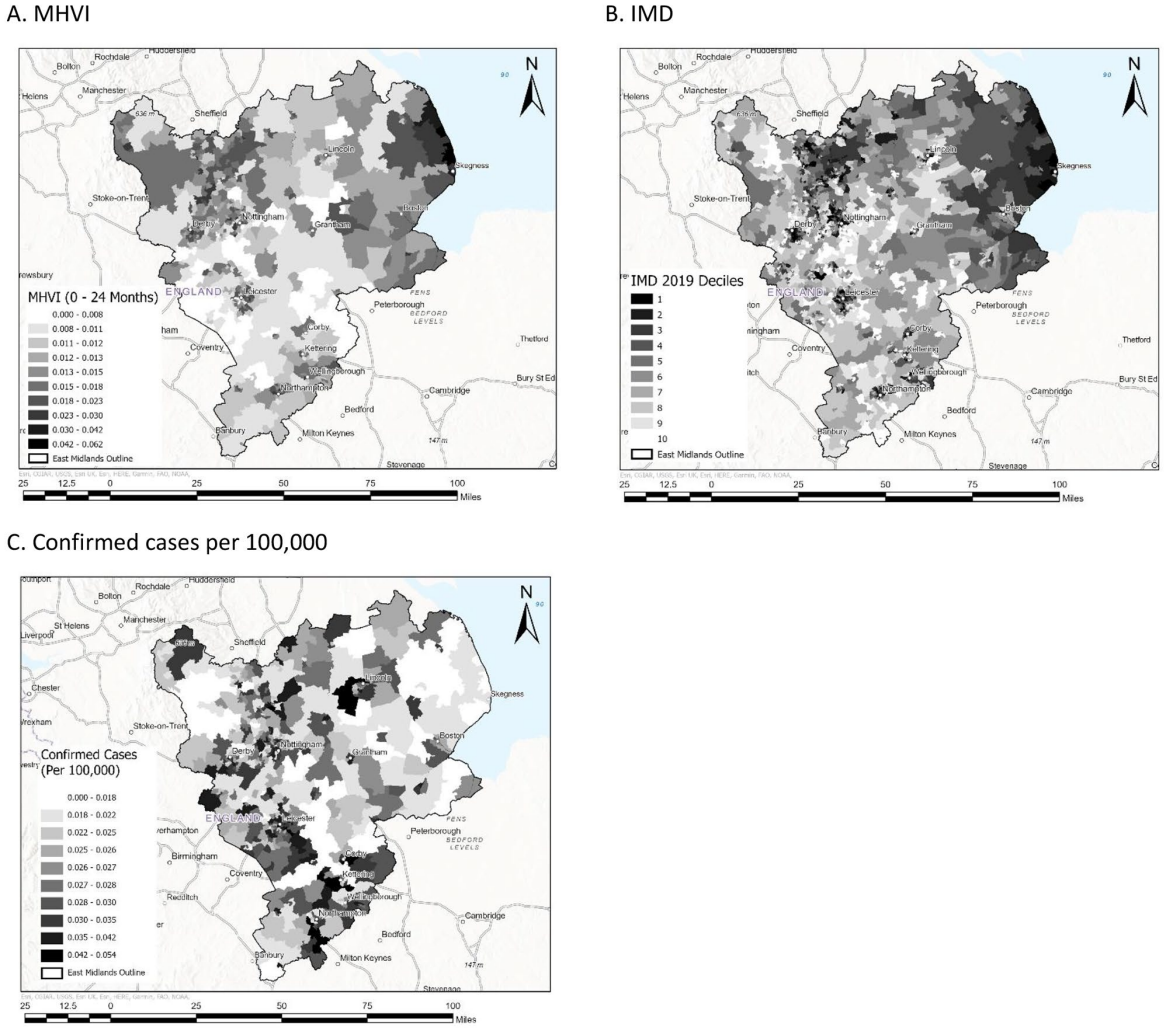
Maps visually comparing the spatial distribution of MHVI scores (**a**), the Index of Multiple Deprivation (**b**), and confirmed cases of COVID-19 based on GOV.UK testing data (**c**)

The second aim of the research was to consider the utility of our novel index for identifying vulnerable regions by comparing the geospatial distribution and patterns of the MHVI to other indices that are often used as proxies for, or measure of vulnerability to severe illness from diseases like COVID-19. Thus, two additional maps are displayed in Fig. 4 alongside the MHVI map (Fig. 4a) for the purpose of comparative analysis. The first additional map is of case rates per 100,000 people based on GOV.UK records of confirmed COVID-19 cases (Fig. 4c). The values for positive case-rates per 100,000 at MSOA scale are presented in S-2 (Table S5). The second additional map displays the Index of Multiple Deprivation scores for the study region, including all MSOAs that appear in the full dataset capturing both EMAS and GOV.UK cases occurring between May 18th, 2020, and April 2nd, 2022 (Fig. 4b). The IMD map was used to explore the utility of the MHVI for identifying vulnerable regions; deprivation is a widely accepted proxy measure of vulnerability to severe illness [52], including from COVID-19 [46]. This visual comparative analysis was used to ground-truth our novel metric, the MHVI.

In addition to the visual analysis presented above, we considered the strength and direction of relationship between the MHVI scores and IMD decile scores for each MSOA by computing the Pearson correlation coefficient. In addition to MHVI scores, Table S5 in S-2 presents the IMD decile scores for each MSOA included in the research (n = 569). The analysis found a moderate negative correlation between IMD decile scores and MHVI scores, r=-.51, *p* < .01, N = 569. Given that lower IMD scores indicate greater deprivation and higher MHVI scores indicate greater vulnerability, this finding suggests that deprivation is associated with greater vulnerability at an area level.

## Discussion

Emerging infectious diseases occurring in developed regions of the modern world vary considerably from those of the past; compared to diseases with very high infection fatality rates like cholera [25, 26] and smallpox [53], the risk of mortality from COVID-19, influenza, and other SARS viruses varies widely; the infection fatality rate is low for the general population, with considerably higher risk of mortality for aging populations [54] and people with pre-existing health conditions [55]. The COVID-19 virus displays unique epidemiological traits, like a longer incubation period than influenza, and the virus is most contagious before symptoms present [56]. These viral characteristics have proved particularly challenging for mitigation; in the early phase of the pandemic the spatial nature of vulnerability, including how to prioritise regional investment and mitigation measures, was not immediately obvious. As the pandemic progressed, the effect of human behaviour on viral-host dynamics became evident; the ‘rush to the pub’ in advance of the first national UK lockdown, outdoor space crowding in the summer months [57], and the regional migration from urban to rural areas [58] increased case rates as well as the exposure of vulnerable communities. In this rapidly evolving context, traditional measures of vulnerability like case rates and mortality, are susceptible to error. The current research develops a novel early warning precision public health equivalent to the infection fatality rate, which controls for changing levels of exposure. In the following, we consider the geospatial patterns of vulnerability identified by mapping the MHVI including the characteristics of highly vulnerable regions, and explore the utility of the MHVI compared to other validated indices.

### Geospatial patterns identified by the MHVI

**Fig. 5:**
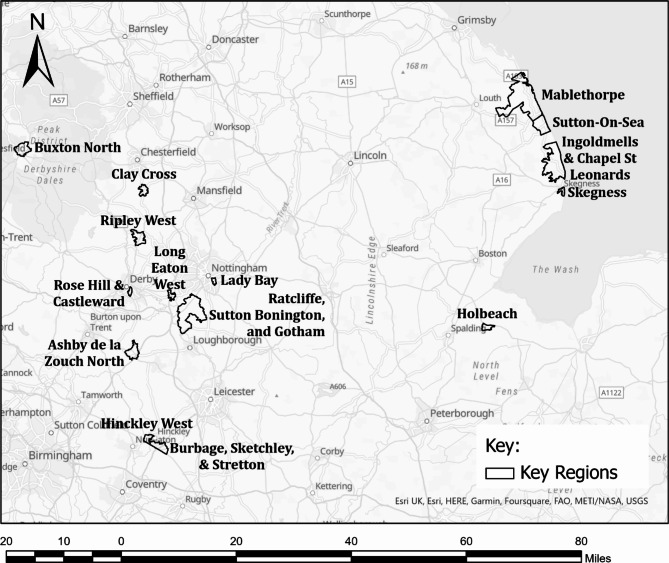
Map of the East Midlands region emphasizing 14 MSOA regions of interest

Above we presented four maps visually displaying the MHVI scores computed using GOV.UK weekly rolling positive COVID-19 cases and EMAS records of cases of suspected severe illness from COVID-19, including a map showing scores for the complete dataset (Fig. 2), and maps showing scores for each of three cumulative periods (Fig. 3) preceding the final complete dataset. Here, we outline some observations about the location and characteristics of regions with highest and lowest vulnerability scores, and consider the time period over which our index becomes a reliable reflection of longer-term trends. The analysis below highlights some key regions of interest in the East Midlands area. These regions are emphasized in Fig. 5.

### Most and least vulnerable areas

Our first observation about the spatial distribution of MHVI scores computed from the complete dataset is that there are three interrelated defining features of regions associated with high and low vulnerability; the degree of deprivation, rural-urban dynamics, and the economic characteristics of regions, including the nature of current economic activities within the region, and historic legacies of economic transformations.

Overall, we found a moderate negative correlation between MHVI scores and IMD decile scores, suggesting that higher MHVI scores (indicating great vulnerability) are more likely to occur in regions with lower IMD scores (indicating greater deprivation). Degree of deprivation and vulnerability reflects the urban-rural characteristics of regions. Without exception, the top twenty least vulnerable regions are categorised in the most affluent IMD deciles, with 19 categorised in the top three most affluent deciles (deciles 8–10). With five exceptions, least vulnerable regions are classified as rural villages in dispersed areas or rural towns on the fringe of mixed urban-rural land uses [59]. The exceptions include three urban areas situated in rural hinterlands such as extensive nature reserves and agricultural areas (Burbage Sketchley & Stretton; Long Eaton West; Ashby de la Zouch North). Only two of least vulnerable regions are located in wider urban landscapes (Hinckley West; Lady Bay).

Nearly all least vulnerable regions are former agricultural areas that have retained characteristics of rurality and undergone processes of rural gentrification whereby the resident rural population is displaced by ex-urban populations with greater capital [60]. These affluent rural villages and towns are often located in the rural hinterlands of major cities, like Ratcliffe, Sutton Bonington and Gotham located in the rural hinterlands of Nottingham. Exceptions include Hinckley West and Lady Bay which have industrial legacies. Notably, Hinckley West is also the least affluent of the low-vulnerability regions. However, most of the least vulnerable regions are characterised by affluence, rurality, and the likely transition from predominately agricultural societies to gentrifying middleclass societies [60]. The process of urban-rural in-migration has been particularly prolific in the East Midlands compared to other regions of the UK [61], as original residents are superseded by younger families and professional couples who often commute to nearby larger urban areas for employment [62].

By comparison, the majority of most vulnerable regions are classified as urban areas, with the exception of Holbeach which is a rural town with urban fringe hinterland. Of these, approximately one quarter have industrial legacies, one quarter are located on the Lincolnshire coastline with tourism legacies, and the remainder were agricultural, or priory settlements subsumed into urban areas by degrees. In contrast to least vulnerable regions, the socio-economic characteristics of most vulnerable areas reflect the heterogeneity of COVID-19 impacts; while the majority are located in deprived urban centres and peripheries, nearly one quarter of areas identified as most vulnerable are categorised as affluent (in the top five IMD deciles), including the rural town of Holbeach. These observations may explain the only moderate correlation between deprivation and vulnerability reported above. The economic heterogeneity of vulnerable areas is consistent with the known demographic dynamics of severe illness from COVID-19, in particular the risk to aging communities [54]. Comparatively affluent vulnerable regions like Ripley West in the Amber Valley, and Holbeach in Lincolnshire are characterised by a higher proportion of aging population compared to surrounding areas [63, 64].

Geospatial trends associated with vulnerability and deprivation also reflect the complex economic history of regions. Four of most vulnerable regions (IMD deciles 1–5) are characterised by ‘post-industrial towns’, non-metropolitan urban areas including Clay Cross, Rose Hill and Castle Ward, and Buxton North in Derbyshire, typically associated with low pay, low skilled service sector employment [65]. Five of most vulnerable regions (IMD deciles 1–2) are declining former coastal tourism destinations located along the Lincolnshire coastline. Based on current economic conditions, these geospatially distant post-industrial and declining coastal areas are similarly considered as ‘left behind’ of ‘forgotten’ places with few opportunities for social mobility [66–68]. However, they also share a unique legacy.

During the early 20th Century, large areas of Nottinghamshire and Derbyshire contained coalfields, with communities developing around single coalmines. These communities were devastated by colliery closures from the 1980s onwards, and many have never fully recovered [69, 70]. Seaside towns on the Lincolnshire coast, like Skegness, Ingoldmells, Chapel St Leonards, Mablethorpe and Sutton-On-Sea, developed as popular tourist resorts in the 19th century, principally serving the industrial working-class of the East Midlands [71]. Similar regional organisation of industrial workforces and resort towns can be identified in Lancashire with Blackpool, Yorkshire with Scarborough, and Bridlington and London with Margate, Southend-on-Sea and Brighton. The links between Lincolnshire coastal towns and the industrial East Midlands is evident in the Derbyshire Miners Convalescent Home in Skegness, which served as a health retreat for Derbyshire miners recovering from illness for 90 years [72]. Following deindustrialisation, families with mining legacies often relocated to former coastal holiday destinations like Skegness. Thus, the historic connectivity between coastal and industrial regions is mirrored by contemporary geospatial health patterns, highlighting the importance of contextualising routinely collated medical data. These patterns are not intuitive. Coastal towns are typically perceived as sites of restoration [73] while post-industrial regions are often associated with high rates of underlying health issues [74, 75].

Overall, the characteristics of most and least vulnerable regions identified by the MHVI highlight distinctions between rural and urban areas, more affluent and more deprived areas, and areas that have undergone gentrification compared to those that have suffered economic hardship associated with deindustrialisation and the decline of coastal tourism. Urban density and deprivation are often considered as indicators of high risk to severe illness from disease [76]. In the UK, younger and older generations are heavily segregated into urban and often-affluent rural areas respectively [35]. Thus, given the epidemiology of COVID-19 and the susceptibility of aging communities [54], both deprived urban and affluent rural communities are vulnerable to severe illness from the virus [5].

### Reliability of MHVI scores across cumulative periods

Analysis of maps displaying MHVI scores for each of three cumulative periods compared to the final complete dataset suggests that while scores for some most and least vulnerable regions remain consistently high (e.g., Skegness on the east coast, Clay Cross in Derbyshire) or low (e.g., west of Derby towards Stoke-on-Trent) over time, most scores stabilise between 11 and 17 months into the pandemic. Overall, geospatial patterns from MHVI scores during the first cumulative period (May 18th to October 31st, 2020) exaggerate the severity of vulnerability, particularly in the south and between Boston and Peterborough in the east. This is probably due to the slow dissemination of community testing in the months following the introduction of testing beyond Public Health England level 3 laboratories [77]. A Similar observation is made by Davenport and colleagues [42] who calculated a case fatality rate (CFR) for England in the early months of the pandemic and concluded that their values were likely to be an overestimation of how lethal the virus was due to testing being limited to key workers and only seriously ill patients at this time [42].

In contrast, ambulance use for severe illness from COVID-19 was more consistent than rates of testing. Not all patients attended by ambulances for COVID-19 were considered severely ill and transported to hospital [78]. However, throughout the pandemic, patients experiencing severe illness from the virus were more likely to be admitted to hospital via ambulance conveyance rather than walk-in, partly due to government guidance and mobility restrictions limiting taxi and public transport use [79]. Thus, although ambulance data is indicative of numbers of patients experiencing severe illness rather than an accurate measure, the degree of accuracy was more stable over the study period compared to positive case records, for which accuracy varied considerably depending on the extent of community testing. As a result, the ratio of ambulance attendances to all positive cases occurring in the first cumulative period (Fig. 3a) is artificially high in multiple regions.

Our observations about MHVI scores over successive cumulative periods are illuminating for several reasons. Firstly, regions with vastly different MHVI scores in the first 5 months compared to the first 11 months of the pandemic, such as areas in rural Lincolnshire, may have experienced longer delays with the expansion of community testing. Further research could examine the root cause of health service disparities related to establishing testing centres in those regions. Secondly, most vulnerable regions with connected industrial-coastal legacies identified from the full dataset map are consistently some of the most vulnerable regions in each iteration of the MHVI, which may indicate community characteristics related to susceptibility to severe illness from the virus, such as pre-existing health conditions [74] and older age [54].

A final point of comparison between MHVI scores for each cumulative time period (Fig. 3) and the complete dataset is related to the nature of scores; over time, and as the size of the dataset examined increases, the MHVI values decrease substantially. For the first cumulative period (Fig. 3a), the maximum score represented on the map in red indicating greatest vulnerability, is 0.636364, suggesting that in most vulnerable regions, more than 60% of all positive cases were likely to have required ambulance attendance for severe illness from COVID-19. For the second cumulative period (Fig. 3b), the highest score indicating greatest vulnerability was 0.2, suggesting that 20% of all positive cases were likely to have required ambulance attendance. The maximum score for the third cumulative period was 0.112727, suggesting approximately 10% of positive cases requiring ambulance attendance. For the final complete dataset (Fig. 3c), most vulnerable regions are characterised by a score of 0.061585, indicating that approximately 0.6% of all positive cases are likely to have required ambulance attendance for severe illness. This final score reflecting greatest vulnerability across the entire dataset from May 18th, 2020 to April 2nd, 2022 is consistent with the average infection fatality rate (IFR) for the virus internationally. Meyerowitz-Katz and Merone [80] conducted a meta-analysis of 24 studies estimating the COVID-19 IFR and concluded the average rate across a wide range of geographical regions was 0.68%, with a range of 0.53–0.82%. Thus, while the accuracy of the MHVI computed from the complete dataset containing nearly two years’ worth of records is comparable to more widely established infection fatality measures, earlier iterations based on smaller data sets are less reliable. However, geospatial analysis of cumulative datasets may elucidate genuine regional vulnerability inequalities with important implications for prioritising mitigation efforts. The merit of our index is highlighting regions that are comparatively more or less vulnerable than others in real time.

### Validity and utility of the MHVI for precision public health

#### Comparative analysis for exploring validity

Common geospatial indicators of vulnerability to severe illness from COVID-19 include measures of deprivation [47] like the IMD [46], positive case rates [15], and mortality rates [31]. To validate the utility of our novel index for precision public health, we compared the spatial distribution of the MHVI to that of the IMD and positive case rates per 100,000 population for the same region. We also considered similarities and differences between our index and an alternative mortality-based index published by Daras et al. [31] for the whole of England. All comparative analyses were conducted at the same scale, MSOA.

Deprivation often reflects underlying susceptibility related to factors like prior multimorbidity [81] and poor health literacy [82]. However, in the case of COVID-19, affluent aging populations are also vulnerable to severe illness [5]. Overall, we observe strong continuity between the geospatial trends of the MHVI and the IMD for most deprived regions like Skegness on the Lincolnshire coastline. However, unlike the IMD, the MHVI also identifies more affluent regions with aging communities, such as areas in the Peak District.

Positive case rates reflect exposure to a virus rather than vulnerability to severe illness. Unsurprisingly, the geospatial distribution of the MHVI varies considerably from that of positive case rates (Fig. 4). Regions like the Lincolnshire coastline and some rural areas in the Peak District have low rates of positive cases while the MHVI identifies these areas as highly vulnerable based on the proportion of all cases that are likely to have been severe. However, in some areas the MHVI and case-rate data converges. For example, some urban areas identified as highly vulnerable by the MHVI, such as the cluster in the north of Nottinghamshire and the post-industrial town of Buxton in Derbyshire, also experienced high positive case rates per population. Thus, the geospatial patterns demonstrated by the MHVI and positive case rates overlap in vulnerable urban regions, which are likely to have experienced greater exposure to the virus [83], and deviate considerably in more isolated rural and coastal regions.

The distribution of mortality from COVID-19 also demonstrates some similarities and differences to the MHVI, indicative of varying exposure between regions. Like case rates, similarities exist between mortality rates per population and the MHVI for regions that are likely to have experienced high exposure like urban areas (e.g., Buxton). In contrast, patterns of vulnerability in more isolated locations vary between the two indices, such as for the Lincolnshire coastline. Both Skegness and Mablethorpe are characterised by extreme deprivation and larger than national average aging populations [84]. According to Daras et al. [31], Mablethorpe experienced considerably higher mortality per population from COVID-19 than Skegness. In contrast, our MHVI suggests these regions have experienced similarly high vulnerability in relation to the proportion of cases that are likely to be severe.

The difference in vulnerability classification between the mortality index and our MHVI for these two coastal towns may be due to the confounding issue of exposure. The population of Mablethorpe is nearly half that of Skegness with more than double the population density [85]. Given that contagious disease spreads more rapidly in higher density environments [76], it is likely that a larger proportion of vulnerable people per population were exposed to, and subsequently died from the virus in Mablethorpe compared to Skegness. Thus, considering mortality rates without controlling for factors that impact exposure, such as population density, as opposed to population size, may misrepresent underlying vulnerability; vulnerable individuals in more diffuse geographical landscapes have less chance of contracting a contagious disease [42].

In summary, our analysis suggests that the MHVI captures the nuances and complexities of vulnerability related to a novel virus with heterogenous effects across the demographic, socio-economic, and geographic dynamics of populations in the UK. By comparison, standard measures of vulnerability, like the IMD [42], only identify a proportion of most vulnerable regions, and these tend to be in high population density areas.

#### Utility of the MHVI for precision public health

The MHVI could be used to improve the reliability of other data sets that emerged prior to accurate measures of positive case rates, such as patient self-reports of symptoms lodged through the NHS COVID-19 phone application, for the purpose of informing service delivery and prioritising resources like personal protective equipment (PPE) for medical staff, particularly in low-density areas with high vulnerability like rural and coastal Lincolnshire where health services may be less prepared for pandemic-related medical emergencies. For example, during the early phase of the pandemic when PPE was scarce, the MHVI could identify regions, outside of the most obvious high-density urban centres, where primary care staff are most likely to encounter extremely vulnerable patients. This analysis could facilitate the allocation of resources to most vulnerable regions for preventing contagion within medical environments like hospitals, nursing homes, and general practices. To support the utility of the MHVI for precision public health, the geospatial patterns presented here should be considered alongside other COVID-19 vulnerability analyses, such as studies that include adjustment for demographic variables [31]. Taken together, IFRs and vulnerability analyses that elucidate relationships between risk factors and health outcomes are a more holistic representation of pandemic health burden than either approach independently.

High rates of ambulance attendance for severe illness from a novel coronavirus may also indicate areas where additional support from primary care providers could help to prevent mortality. Not all patients attended by ambulances for suspected severe illness from COVID-19 are conveyed to hospital; the attending paramedic may decide that the condition of the patient does not require immediate medical attention. In some cases, those non-conveyed patients have been found to experience worsening illness over the following month resulting in mortality [43]. Our index could act as an early warning system for prioritising follow-up consultation services for non-conveyed patients (E.g., telehealth services), particularly in regions with high proportions of people experiencing severe illness. In the event of a future pandemic with similar epidemiological traits, professional networks like the UK-wide 999 Research Forum and National Ambulance Research Steering Group (NARSG), are well placed to harness existing collaborations between emergency service providers and research groups to conduct rapid analysis of ambulance data. Currently, all UK ambulance Trusts have data share agreements with higher educational institutes coordinated through NARSG.

Importantly, the characteristics of contagious diseases vary. For example, compared to influenza, COVID-19 has a longer incubation period, is more contagious with highly heterogenous health outcomes, infected individuals are more likely to be asymptomatic than experience severe symptoms, and the virus is most contagious before symptoms present [56]. Thus, the MHVI will be most useful for predicting vulnerability during pandemics involving contagious diseases with similar etiological and epidemiological traits as COVID-19. In such cases, regions with high MHVI scores, indicating extreme vulnerability, could be targeted for educational interventions to raise awareness about worsening symptoms. Botan et al. [86] found that a leaflet-based educational intervention about diabetes management was effective for reducing repeat use of ambulance services for hypoglycaemic events. A similar approach could be used to inform non-conveyed COVID-19 patients in highly vulnerable regions of symptoms associated with rapid deterioration, such as low blood-oxygen levels, and to encourage home self-assessment, including the use of smart-phone based breathing sound apps that detect declining lung capacity [87]. App development for identifying unhealthy breathing associated with COVID-19 is currently underway [88].

Over the longer-term, the geospatial trends elucidated by the MHVI offer insights to the underlying drivers of vulnerability, and the importance of society-environment relationships. The characteristics of the spaces that people inhabit, and the legacy of those spaces influences contemporary health inequalities that may be obscured by one-dimensional indices like case-rates or mortality alone. Some regions, like coastal towns, that experience challenges associated with contemporary conditions, such as poor access to healthcare, are also faced with the enduring and inter-generational health profiles of communities. Regions at the intersection of past and current risk factors for poor health outcomes like Mablethorpe and Skegness [89] are an increasing priority for wider investment to improve social mobility beyond the timescale of the pandemic.

### Limitations

Using ambulance data as a proxy measure of severe illness from COVID-19 encounters similar limitations to other routine clinical data, such as self-reported data collated via the NHS Test and Trace App. In particular, patient self-assessments of illness are often inaccurate. However, the clinical judgement of paramedics attending medical emergencies is guided by Public Health England’s case definition symptom criteria for COVID-19 diagnosis, which includes measures that have been proven to predict positive COVID-19 case rates with a high degree of accuracy [5]. These measures, such as self-reported olfactory and taste dysfunction [90, 91] and blood oxygen levels [49], are routinely collected by paramedics and used to inform decisions about whether a patient is likely to be experiencing illness from the virus.

While spatial data can indicate socio-economic and landscape related factors that might explain severe illness, these data cannot determine causality. Qualitative research is required to ground truth our findings. Further, it is likely that in addition to geographic and the area-level socio-economic characteristics of regions, other factors, such as the demographic characteristics of regions, also influence vulnerability. For instance, conditions related to ethnicity almost certainly increase vulnerability, including multi-generational occupancy housing [91, 92], increased exposure due to low-income employment in ‘essential’ services [93], and higher rates of pre-existing co-morbidities [94]. However, these dynamics cannot be determined from aggregate anonymised datasets and would require costly, time-consuming data linkage that is unlikely to be achieved in real-time.

Factors beyond the scope of this study may affect ambulance use, such as close proximity to hospitals with accident and emergency services [5], and willingness to call an ambulance, which is often related to deprivation and health literacy, including ability to recognize severe symptoms of escalating illness [82]. The underreporting of cases and mortality is well documented [95]. Using South Korea as a benchmark region to adjust case rates for underreporting, Jagodnik et al. [96] estimate that case rates in the UK during 2020 were likely to have been more than 14 times greater than reported rates. Thus, we recognize that our index is indicative of *comparative* geospatial vulnerability trends, rather than an *absolute* measure of COVID-19 cases. Future research could involve adjusting the MHVI for underreporting, although to be meaningful this process would need to consider regional variation in underreporting, such as differences between more affluent and more deprived areas.

Finally, further research is needed to validate the MHVI beyond the epidemiological context of the COVID-19 pandemic and the regional context of the East Midlands of the UK. A starting point would be to replicate the research for other regions of the UK and other regions of the world with centralised ambulance services.

## Conclusions

Vulnerability to severe illness from contagious disease occurs at the nexus of underlying susceptibility to poor health outcomes and exposure to a virus [5]. The most reliable measure of vulnerability is the proportion of overall positive cases resulting in mortality [40]. However, at the height of a pandemic, the data required for computing a case fatality rate (CFR) may not be available in the timeframe required, or at the geographical scale, for decision making. Further, while a CFR based on mortality records suggest opportunities for localised mitigation efforts, such as enforcing regional lock-down, this approach is somewhat retrospective; there is no avenue for intervention to prevent the escalation of illness to mortality. Our index uses ambulance data and adapts the concept of a CFR for real-time precision public health. In summary, our analysis of geospatial trends over the first nearly two years of the COVID-19 pandemic emphasize the following key points:

- The geospatial distribution of the MHVI suggests that vulnerability transcends socio-economic, and rural-urban boundaries, challenging many of the traditional assumptions about contagious disease disproportionately impacting more deprived and more urban regions;
- Iterations of the MHVI computed from data collated in early phases of the pandemic experience similar inaccuracies to other indices developed from alternative measures of vulnerability during the same period. However, the final iteration, computed from nearly two years’ worth of data, is consistent with international CFRs computed using mortality records;
- Unlike traditional methods of computing CFRs, the MHVI presents opportunities for intervention, including prioritising PPE and educational programmes to prevent the worsening health of vulnerable patients, as well as protecting health care staff;
- The MHVI captures geospatial dynamics that single metrics alone often overlook, including the compound health challenges associated with deprived and declining coastal towns inhabited by communities with post-industrial health legacies;

In the context of emerging infectious disease in the modern world, knowledge is cumulative and all possible avenues for protecting societies should be embraced. The global evidence base is convergent, and our understanding of host-society dynamics is evolving as we continue to triangulate our knowledges and datasets from across numerous research disciplines, institutions, and regions. Our contribution to precision public health is twofold; we introduce a historic geographical component to the narrative around vulnerability, and also highlight the merits of pre-hospital data for optimising health service delivery, identifying vulnerable communities in real-time, and intervention to prevent mortality. We hope that others working with ambulance data will replicate our methodologies and ground truth our approach beyond the UK. The geospatial analysis of ambulance data is in its infancy; the opportunities for future-proofing our societies against the threat of novel diseases are limitless.

### Electronic supplementary material

Below is the link to the electronic supplementary material.

Supplementary Material 1

Supplementary Material 2

Supplementary Material 3

## Data Availability

The research involves two datasets; one obtained from GOV.UK (https://coronavirus.data.gov.uk/details/download) and the other obtained from the East Midlands Ambulance Service. This data cannot be made publicly available due uploading the terms of data share agreements. Contact: Harriet Elizabeth Moore, HaMoore@lincoln.ac.uk.
