# Supplementary material for: An early warning precision public health approach for assessing COVID-19 vulnerability in the UK: the Moore-Hill Vulnerability Index (MHVI)
Source: BMC Public Health. 2023 Nov 2;23:2147. doi: 10.1186/s12889-023-17092-7 (PMC10623819; doi:10.1186/s12889-023-17092-7)
Supplement: Supplementary file 1 — Supplementary Material 1 [file 12889_2023_17092_MOESM1_ESM.docx]

S-3

**Data harmonisation steps**

*Stage one: harmonising full dataset*

1. EMAS Database including records of date and MSOA for each attendance received from 18^th^ March 2020 to 2^nd^ April 2022 (n=33311).

2. 430 records deleted because missing dates of ambulance attendance (n=32881). An additional 51 records were removed because they were missing MSOAs, likely due to the inclusion of records of ambulance attendance outside of the official East Midland borders, such as Yorkshire and the Humber (n=32830). This is because EMAS service areas cover historic county line boundaries while government records use ONS geocodes for areas based off of the 2001 output areas from the UK census. On average, LSOAs typically have a population of 1500 people, while MSOAs have a population of 7200.

3. 10742 records were removed from the EMAS dataset because they do not fall within the study period of May 18^th^, 2020, to April 2^nd^, 2022 (n=22088).

4. Confirmed cases, including dates and MSOA, were downloaded from GOV.UK in the form of weekly rolling cumulative numbers (i.e., a value for the number of cases per week), from the week beginning 18^th^ of May to the week ending April 2^nd^ (n=1507793).

5. Data harmonisation was performed in three steps (See S-1, Table S1). Firstly, EMAS and GOV.UK data joined via MSOA. Secondly, ambulance attendance in 64 MSOAs (n=1364) were removed from EMAS data because they did not appear in GOV.UK data (n=20724). Thirdly, records from four MSOAs (E02004093, E02004094, E02004095, E02004096; n=11154) in GOV.UK data were excluded because these areas do not appear in EMAS data, likely due to these services areas (n=1496639). These regions lie within the Peak District, and it is possible that ambulances did not attend because other local services were more accessible and convenient. The remaining number of EMAS cases was 20724 and the remaining number of GOV.UK cases was 1496639 for the period 18^th^ of May 2020 to 2^nd^ April 2022.

*Stage two: harmonising sub-sets by cumulative intervals*

Following Step 4 above, sub-sets of data were extracted from the main dataset for the purpose of producing cumulative MHVI datasets and maps displaying change over time.

1. Data sub-set for first cumulative period: All records of suspected severe COVID-19 cases attended by ambulances (EMAS data) and all records of confirmed cases (GOV.UK) between May 18^th^ and October 31^st^ (approximately 0-5months) were extracted. This subset included 6,821 EMAS records of ambulance attendance for suspected severe COVID-19 and 68,116 GOV.UK records of confirmed COVID-19 cases.

2. Data harmonisation was performed in two steps (see S-1, Table S2). The first step involved removing records from two MSOAs (E02003460, E02005924) that appeared in EMAS data but did not appear in GOV.UK data for the time-period (n=2 cases). The second step involved removing records from 5 MSOAs (E02004093, E02004094, E02004095, E02004096, E02005851) that appeared in GOV.UK data but did not appear in EMAS data for the time-period (n=728 cases). The final sub-set of data for the first cumulative period included 6,819 EMAs records of ambulance attendance and 67,388 records of confirmed cases.

3. Data sub-set for second cumulative period: All records of suspected severe COVID-19 cases attended by ambulances (EMAS data) and all records of confirmed cases (GOV.UK) between May 18^th,^ 2020, and April 17^th^, 2021 (approximately 0-12 months) were extracted, including 16,758 EMAS records and 313,523 GOV.UK records.

4. Data harmonisation was performed in two steps (See S-1, Table S3). The first step involved removing records from 11 MSOAs that appeared in EMAS data but did not appear in GOV.UK data (n=12 cases). The second step involved removing records from four MSOAs (E02004093, E02004094, E02004095, E02004096) that appeared in GOV.UK data but did not appear in EMAS data (n=1866). The final sub-set of data for the second cumulative period included 16,746 EMAS records and 311,657 GOV.UK records.

5. Data sub-set for third cumulative period: All records of suspected severe COVID-19 cases attended by ambulances (EMAS data) and all records of confirmed cases (GOV.UK) between May 18^th^, 2020 and October 2^nd^, 2021 (approximately 0-17 months) were extracted, including 18,946 EMAS records and 583,842 GOV.UK records.

6. Data harmonisation was performed in two steps (See S-1, Table S4). The first step involved removing records from 11 MSOAs that appeared in EMAS data but did not appear in GOV.UK data (n=12 cases). The second step involved removing records from four (E02004093, E02004094, E02004095, E02004096) that appeared in GOV.UK data but did not appear in EMAS data (n=4431 cases). The final sub-set of data for the third cumulative period included 18,934 EMAS records and 579,411 GOV.UK records.
