# Supplementary material for: An early warning precision public health approach for assessing COVID-19 vulnerability in the UK: the Moore-Hill Vulnerability Index (MHVI)
Source: BMC Public Health. 2023 Nov 2;23:2147. doi: 10.1186/s12889-023-17092-7 (PMC10623819; doi:10.1186/s12889-023-17092-7)
Supplement: Supplementary file 2 — Supplementary Material 2 [file 12889_2023_17092_MOESM2_ESM.docx]

S-2

**Table S5.** Values for each MSOA, including MHVI (calculated from cumulative from weekly GOV.UK positive case numbers and daily EMAS suspected severe illness case numbers), case-rates per/100,000 population (calculated from weekly GOV.UK positive case records) between March 18^th^, 2020 and April 2^nd^, 2022, and aggregate IMD Decile.

| **MSOA** | **GOV.UK** | **EMAS** | **MHVI** | **Confirmed cases Per/100,000** | **IMD Decile** |
| --- | --- | --- | --- | --- | --- |
| E02002796 | 1932 | 20 | 0.010352 | 0.01932 | 10 |
| E02002797 | 2174 | 33 | 0.015179 | 0.02174 | 10 |
| E02002798 | 3375 | 38 | 0.011259 | 0.03375 | 10 |
| E02002799 | 2192 | 34 | 0.015511 | 0.02192 | 3 |
| E02002800 | 2414 | 25 | 0.010356 | 0.02414 | 8 |
| E02002801 | 2438 | 51 | 0.020919 | 0.02438 | 5 |
| E02002802 | 2606 | 36 | 0.014198 | 0.02606 | 1 |
| E02002803 | 2349 | 41 | 0.017454 | 0.02349 | 3 |
| E02002804 | 2954 | 45 | 0.015234 | 0.02954 | 3 |
| E02002805 | 1709 | 20 | 0.011703 | 0.01709 | 10 |
| E02002806 | 1909 | 38 | 0.019906 | 0.01909 | 3 |
| E02002807 | 2912 | 39 | 0.014080 | 0.02912 | 4 |
| E02002808 | 3791 | 53 | 0.013980 | 0.03791 | 2 |
| E02002809 | 2377 | 53 | 0.022297 | 0.02377 | 4 |
| E02002810 | 2922 | 52 | 0.017796 | 0.02922 | 10 |
| E02002811 | 2881 | 68 | 0.023603 | 0.02881 | 1 |
| E02002812 | 2695 | 41 | 0.015213 | 0.02695 | 5 |
| E02002813 | 3152 | 87 | 0.027602 | 0.03152 | 1 |
| E02002814 | 2356 | 35 | 0.014856 | 0.02356 | 10 |
| E02002815 | 2237 | 43 | 0.019669 | 0.02237 | 2 |
| E02002816 | 3264 | 42 | 0.012868 | 0.03264 | 9 |
| E02002817 | 1940 | 43 | 0.022165 | 0.0194 | 8 |
| E02002818 | 2405 | 33 | 0.013721 | 0.02405 | 2 |
| E02002819 | 2476 | 48 | 0.019386 | 0.02476 | 4 |
| E02002820 | 3130 | 42 | 0.013419 | 0.0313 | 2 |
| E02002821 | 3821 | 65 | 0.017011 | 0.03821 | 1 |
| E02002822 | 2210 | 46 | 0.020814 | 0.0221 | 5 |
| E02002823 | 2463 | 44 | 0.017864 | 0.02463 | 3 |
| E02002824 | 3354 | 66 | 0.019678 | 0.03354 | 2 |
| E02002825 | 2981 | 41 | 0.013754 | 0.02981 | 6 |
| E02002826 | 2631 | 24 | 0.009502 | 0.02631 | 8 |
| E02002827 | 2296 | 35 | 0.015679 | 0.02296 | 3 |
| E02002828 | 2702 | 50 | 0.018875 | 0.02702 | 5 |
| E02002829 | 4089 | 61 | 0.014918 | 0.04089 | 1 |
| E02002830 | 3759 | 70 | 0.018622 | 0.03759 | 2 |
| E02002831 | 2369 | 58 | 0.024483 | 0.02369 | 4 |
| E02002832 | 2998 | 68 | 0.022682 | 0.02998 | 3 |
| E02002833 | 2506 | 42 | 0.017558 | 0.02506 | 3 |
| E02002834 | 4060 | 47 | 0.011576 | 0.0406 | 6 |
| E02002835 | 5339 | 98 | 0.018543 | 0.05339 | 6 |
| E02002836 | 2825 | 59 | 0.020885 | 0.02825 | 3 |
| E02002837 | 2910 | 34 | 0.012027 | 0.0291 | 1 |
| E02002838 | 2974 | 56 | 0.019166 | 0.02974 | 1 |
| E02002839 | 2916 | 56 | 0.019547 | 0.02916 | 4 |
| E02002842 | 2741 | 31 | 0.011310 | 0.02741 | 2 |
| E02002843 | 3798 | 75 | 0.019747 | 0.03798 | 3 |
| E02002844 | 3377 | 62 | 0.018359 | 0.03377 | 1 |
| E02002845 | 3323 | 80 | 0.024075 | 0.03323 | 2 |
| E02002846 | 2476 | 17 | 0.006866 | 0.02476 | 6 |
| E02002847 | 3216 | 56 | 0.017413 | 0.03216 | 4 |
| E02002848 | 3332 | 62 | 0.018607 | 0.03332 | 2 |
| E02002849 | 4301 | 40 | 0.009300 | 0.04301 | 3 |
| E02002851 | 2854 | 51 | 0.017870 | 0.02854 | 7 |
| E02002852 | 2713 | 42 | 0.015481 | 0.02713 | 1 |
| E02002853 | 4310 | 85 | 0.019722 | 0.0431 | 5 |
| E02002854 | 2987 | 39 | 0.013057 | 0.02987 | 1 |
| E02002855 | 2375 | 18 | 0.007579 | 0.02375 | 4 |
| E02002856 | 3865 | 39 | 0.010091 | 0.03865 | 7 |
| E02002857 | 2418 | 36 | 0.014888 | 0.02418 | 2 |
| E02002858 | 2486 | 45 | 0.018101 | 0.02486 | 10 |
| E02002860 | 2960 | 42 | 0.014189 | 0.0296 | 4 |
| E02002861 | 2717 | 52 | 0.019139 | 0.02717 | 1 |
| E02002862 | 2802 | 53 | 0.018915 | 0.02802 | 1 |
| E02002863 | 2317 | 18 | 0.007769 | 0.02317 | 8 |
| E02002864 | 2473 | 31 | 0.012940 | 0.02473 | 9 |
| E02002865 | 1900 | 19 | 0.010000 | 0.019 | 10 |
| E02002866 | 2055 | 20 | 0.009732 | 0.02055 | 10 |
| E02002867 | 2311 | 14 | 0.006058 | 0.02311 | 9 |
| E02002868 | 1959 | 31 | 0.015824 | 0.01959 | 4 |
| E02002869 | 2523 | 48 | 0.019025 | 0.02523 | 1 |
| E02002871 | 2538 | 48 | 0.018913 | 0.02538 | 2 |
| E02002872 | 2995 | 44 | 0.014691 | 0.02995 | 1 |
| E02002873 | 3385 | 48 | 0.014476 | 0.03385 | 2 |
| E02002874 | 3483 | 50 | 0.014355 | 0.03483 | 2 |
| E02002875 | 2755 | 49 | 0.017786 | 0.02755 | 2 |
| E02002876 | 2498 | 42 | 0.016813 | 0.02498 | 2 |
| E02002877 | 2635 | 44 | 0.017078 | 0.02635 | 5 |
| E02002878 | 3009 | 49 | 0.016284 | 0.03009 | 1 |
| E02002879 | 3172 | 55 | 0.017339 | 0.03172 | 3 |
| E02002880 | 2920 | 46 | 0.016438 | 0.0292 | 1 |
| E02002881 | 2572 | 32 | 0.012442 | 0.02572 | 1 |
| E02002882 | 2798 | 30 | 0.010722 | 0.02798 | 3 |
| E02002883 | 2370 | 44 | 0.018565 | 0.0237 | 2 |
| E02002884 | 2176 | 72 | 0.033088 | 0.02176 | 2 |
| E02002885 | 2603 | 53 | 0.020745 | 0.02603 | 1 |
| E02002886 | 3187 | 57 | 0.017885 | 0.03187 | 3 |
| E02002887 | 2160 | 65 | 0.030093 | 0.0216 | 1 |
| E02002888 | 2225 | 34 | 0.015281 | 0.02225 | 1 |
| E02002889 | 3827 | 33 | 0.008884 | 0.03827 | 2 |
| E02002890 | 3475 | 39 | 0.011799 | 0.03475 | 2 |
| E02002891 | 2559 | 45 | 0.017976 | 0.02559 | 3 |
| E02002892 | 2006 | 43 | 0.021436 | 0.02006 | 1 |
| E02002893 | 4073 | 51 | 0.012521 | 0.04073 | 2 |
| E02002894 | 2385 | 37 | 0.015514 | 0.02385 | 8 |
| E02002895 | 3324 | 30 | 0.009025 | 0.03324 | 6 |
| E02002896 | 3039 | 55 | 0.018098 | 0.03039 | 1 |
| E02002897 | 2455 | 35 | 0.014257 | 0.02455 | 9 |
| E02002898 | 3806 | 94 | 0.025223 | 0.03806 | 6 |
| E02002899 | 3419 | 32 | 0.009359 | 0.03419 | 5 |
| E02002901 | 2107 | 27 | 0.012814 | 0.02107 | 5 |
| E02002902 | 3157 | 50 | 0.015838 | 0.03157 | 2 |
| E02002903 | 1930 | 32 | 0.016580 | 0.0193 | 2 |
| E02002904 | 2302 | 57 | 0.024761 | 0.02302 | 2 |
| E02004029 | 2283 | 40 | 0.017521 | 0.02283 | 3 |
| E02004030 | 1534 | 17 | 0.011082 | 0.01534 | 8 |
| E02004031 | 2349 | 54 | 0.022989 | 0.02349 | 2 |
| E02004032 | 2604 | 40 | 0.015745 | 0.02604 | 8 |
| E02004033 | 2762 | 63 | 0.023172 | 0.02762 | 5 |
| E02004034 | 1892 | 47 | 0.025370 | 0.01892 | 8 |
| E02004035 | 1671 | 17 | 0.010174 | 0.01671 | 8 |
| E02004036 | 3257 | 64 | 0.019650 | 0.03257 | 4 |
| E02004037 | 2553 | 21 | 0.008226 | 0.02553 | 10 |
| E02004038 | 2213 | 42 | 0.018979 | 0.02213 | 6 |
| E02004039 | 2284 | 27 | 0.011821 | 0.02284 | 8 |
| E02004040 | 3115 | 39 | 0.012520 | 0.03115 | 6 |
| E02004041 | 2453 | 43 | 0.017937 | 0.02453 | 6 |
| E02004043 | 2215 | 33 | 0.014898 | 0.02215 | 7 |
| E02004044 | 2900 | 25 | 0.008966 | 0.029 | 10 |
| E02004045 | 2986 | 31 | 0.010382 | 0.02986 | 7 |
| E02004046 | 1876 | 28 | 0.014925 | 0.01876 | 4 |
| E02004047 | 1911 | 33 | 0.017268 | 0.01911 | 2 |
| E02004048 | 2079 | 33 | 0.015873 | 0.02079 | 2 |
| E02004049 | 2315 | 38 | 0.016847 | 0.02315 | 5 |
| E02004050 | 2680 | 40 | 0.015299 | 0.0268 | 2 |
| E02004051 | 3269 | 67 | 0.021107 | 0.03269 | 3 |
| E02004052 | 2753 | 60 | 0.021794 | 0.02753 | 5 |
| E02004053 | 2390 | 34 | 0.014226 | 0.0239 | 4 |
| E02004054 | 2774 | 65 | 0.023432 | 0.02774 | 5 |
| E02004055 | 2046 | 40 | 0.020039 | 0.02046 | 4 |
| E02004056 | 2606 | 54 | 0.020721 | 0.02606 | 2 |
| E02004057 | 2583 | 31 | 0.012002 | 0.02583 | 3 |
| E02004058 | 2451 | 55 | 0.022440 | 0.02451 | 2 |
| E02004059 | 2320 | 38 | 0.016810 | 0.0232 | 5 |
| E02004060 | 2521 | 50 | 0.019833 | 0.02521 | 3 |
| E02004061 | 2217 | 39 | 0.018042 | 0.02217 | 3 |
| E02004062 | 2058 | 23 | 0.011176 | 0.02058 | 4 |
| E02004063 | 2367 | 46 | 0.019856 | 0.02367 | 8 |
| E02004064 | 3114 | 48 | 0.015414 | 0.03114 | 3 |
| E02004065 | 2412 | 29 | 0.012023 | 0.02412 | 10 |
| E02004066 | 3696 | 52 | 0.014069 | 0.03696 | 6 |
| E02004067 | 1797 | 43 | 0.025042 | 0.01797 | 1 |
| E02004068 | 1927 | 25 | 0.012974 | 0.01927 | 10 |
| E02004069 | 1503 | 24 | 0.015968 | 0.01503 | 10 |
| E02004070 | 1457 | 24 | 0.016472 | 0.01457 | 7 |
| E02004071 | 1247 | 22 | 0.017642 | 0.01247 | 8 |
| E02004072 | 2650 | 45 | 0.016981 | 0.0265 | 9 |
| E02004073 | 2185 | 33 | 0.015561 | 0.02185 | 5 |
| E02004074 | 1519 | 18 | 0.011850 | 0.01519 | 8 |
| E02004075 | 1609 | 25 | 0.015538 | 0.01609 | 7 |
| E02004076 | 2277 | 32 | 0.014054 | 0.02277 | 8 |
| E02004077 | 2300 | 18 | 0.007826 | 0.023 | 7 |
| E02004078 | 2079 | 36 | 0.017316 | 0.02079 | 1 |
| E02004080 | 3236 | 49 | 0.015142 | 0.03236 | 3 |
| E02004081 | 1702 | 30 | 0.017626 | 0.01702 | 9 |
| E02004082 | 1920 | 25 | 0.013021 | 0.0192 | 10 |
| E02004083 | 2611 | 23 | 0.009192 | 0.02611 | 5 |
| E02004084 | 1989 | 29 | 0.015083 | 0.01989 | 2 |
| E02004085 | 2760 | 36 | 0.013406 | 0.0276 | 6 |
| E02004086 | 2220 | 19 | 0.009009 | 0.0222 | 8 |
| E02004087 | 2457 | 28 | 0.011396 | 0.02457 | 4 |
| E02004088 | 2394 | 11 | 0.004595 | 0.02394 | 10 |
| E02004089 | 2227 | 32 | 0.014818 | 0.02227 | 3 |
| E02004090 | 1806 | 27 | 0.014950 | 0.01806 | 8 |
| E02004091 | 2875 | 32 | 0.011130 | 0.02875 | 8 |
| E02004092 | 2880 | 25 | 0.009028 | 0.0288 | 6 |
| E02004097 | 2271 | 32 | 0.014091 | 0.02271 | 9 |
| E02004098 | 2198 | 37 | 0.016833 | 0.02198 | 5 |
| E02004100 | 2365 | 34 | 0.014376 | 0.02365 | 9 |
| E02004102 | 2188 | 57 | 0.026508 | 0.02188 | 5 |
| E02004103 | 2313 | 47 | 0.020320 | 0.02313 | 4 |
| E02004104 | 2246 | 35 | 0.015583 | 0.02246 | 9 |
| E02004105 | 2949 | 42 | 0.014581 | 0.02949 | 7 |
| E02004106 | 1907 | 25 | 0.013110 | 0.01907 | 9 |
| E02004108 | 2310 | 41 | 0.017749 | 0.0231 | 6 |
| E02004109 | 1991 | 17 | 0.008538 | 0.01991 | 10 |
| E02004110 | 2409 | 23 | 0.009548 | 0.02409 | 10 |
| E02004111 | 1622 | 18 | 0.012330 | 0.01622 | 5 |
| E02004112 | 1735 | 27 | 0.015562 | 0.01735 | 9 |
| E02004113 | 3091 | 46 | 0.015205 | 0.03091 | 2 |
| E02004114 | 2343 | 38 | 0.016219 | 0.02343 | 8 |
| E02004115 | 2611 | 33 | 0.012639 | 0.02611 | 6 |
| E02004116 | 2679 | 78 | 0.029115 | 0.02679 | 3 |
| E02004117 | 2274 | 34 | 0.014952 | 0.02274 | 5 |
| E02004118 | 2564 | 20 | 0.007800 | 0.02564 | 7 |
| E02004119 | 4147 | 39 | 0.009404 | 0.04147 | 10 |
| E02004120 | 3714 | 54 | 0.014540 | 0.03714 | 8 |
| E02004121 | 3185 | 34 | 0.010675 | 0.03185 | 9 |
| E02004122 | 2115 | 11 | 0.005201 | 0.02115 | 10 |
| E02004123 | 1940 | 17 | 0.008763 | 0.0194 | 9 |
| E02004124 | 2389 | 25 | 0.010883 | 0.02389 | 5 |
| E02004125 | 2662 | 47 | 0.017656 | 0.02662 | 4 |
| E02004126 | 2312 | 34 | 0.014706 | 0.02312 | 5 |
| E02004128 | 3484 | 48 | 0.014064 | 0.03484 | 6 |
| E02005334 | 2188 | 19 | 0.008684 | 0.02188 | 9 |
| E02005335 | 2527 | 20 | 0.007915 | 0.02527 | 10 |
| E02005336 | 2584 | 32 | 0.012384 | 0.02584 | 7 |
| E02005337 | 3059 | 53 | 0.017326 | 0.03059 | 7 |
| E02005338 | 3931 | 40 | 0.010176 | 0.03931 | 7 |
| E02005339 | 2947 | 21 | 0.007126 | 0.02947 | 10 |
| E02005340 | 2792 | 36 | 0.012894 | 0.02792 | 7 |
| E02005341 | 2584 | 21 | 0.008127 | 0.02584 | 9 |
| E02005342 | 2240 | 15 | 0.006696 | 0.0224 | 9 |
| E02005343 | 2622 | 13 | 0.004958 | 0.02622 | 10 |
| E02005344 | 2942 | 24 | 0.008158 | 0.02942 | 9 |
| E02005345 | 1951 | 20 | 0.010251 | 0.01951 | 8 |
| E02005346 | 4043 | 54 | 0.013356 | 0.04043 | 2 |
| E02005347 | 3602 | 59 | 0.016380 | 0.03602 | 6 |
| E02005348 | 1539 | 26 | 0.016894 | 0.01539 | 8 |
| E02005349 | 2501 | 39 | 0.015594 | 0.02501 | 7 |
| E02005350 | 2173 | 37 | 0.017027 | 0.02173 | 7 |
| E02005351 | 3189 | 37 | 0.011602 | 0.03189 | 8 |
| E02005352 | 1965 | 15 | 0.007634 | 0.01965 | 10 |
| E02005353 | 3479 | 32 | 0.009198 | 0.03479 | 4 |
| E02005354 | 3041 | 41 | 0.013482 | 0.03041 | 10 |
| E02005355 | 2362 | 21 | 0.008891 | 0.02362 | 9 |
| E02005356 | 2274 | 37 | 0.016271 | 0.02274 | 9 |
| E02005357 | 2858 | 29 | 0.010147 | 0.02858 | 7 |
| E02005358 | 4026 | 48 | 0.012171 | 0.04026 | 9 |
| E02005359 | 2599 | 25 | 0.010004 | 0.02599 | 9 |
| E02005360 | 1812 | 17 | 0.009382 | 0.01812 | 10 |
| E02005361 | 2089 | 24 | 0.011489 | 0.02089 | 7 |
| E02005362 | 2456 | 34 | 0.013844 | 0.02456 | 6 |
| E02005363 | 3144 | 42 | 0.013359 | 0.03144 | 9 |
| E02005364 | 1721 | 19 | 0.011040 | 0.01721 | 9 |
| E02005365 | 3352 | 62 | 0.018795 | 0.03352 | 6 |
| E02005366 | 2607 | 30 | 0.011507 | 0.02607 | 8 |
| E02005368 | 1657 | 17 | 0.010260 | 0.01657 | 8 |
| E02005369 | 4023 | 38 | 0.009446 | 0.04023 | 10 |
| E02005370 | 3104 | 19 | 0.006121 | 0.03104 | 10 |
| E02005371 | 3075 | 27 | 0.008780 | 0.03075 | 9 |
| E02005372 | 2773 | 14 | 0.005049 | 0.02773 | 9 |
| E02005373 | 3592 | 27 | 0.007517 | 0.03592 | 10 |
| E02005374 | 1826 | 35 | 0.019168 | 0.01826 | 6 |
| E02005375 | 3048 | 44 | 0.014764 | 0.03048 | 10 |
| E02005376 | 3224 | 34 | 0.010546 | 0.03224 | 9 |
| E02005377 | 2729 | 25 | 0.009894 | 0.02729 | 8 |
| E02005378 | 1759 | 22 | 0.012507 | 0.01759 | 10 |
| E02005379 | 1939 | 18 | 0.009283 | 0.01939 | 9 |
| E02005380 | 2523 | 12 | 0.004756 | 0.02523 | 9 |
| E02005381 | 3000 | 16 | 0.005333 | 0.03 | 8 |
| E02005382 | 3148 | 23 | 0.007306 | 0.03148 | 5 |
| E02005383 | 2888 | 24 | 0.008310 | 0.02888 | 5 |
| E02005384 | 2259 | 17 | 0.007525 | 0.02259 | 8 |
| E02005385 | 2013 | 16 | 0.007948 | 0.02013 | 9 |
| E02005386 | 2924 | 35 | 0.011970 | 0.02924 | 5 |
| E02005387 | 3207 | 19 | 0.005925 | 0.03207 | 7 |
| E02005388 | 1833 | 17 | 0.009274 | 0.01833 | 5 |
| E02005389 | 1698 | 20 | 0.011779 | 0.01698 | 5 |
| E02005390 | 3221 | 13 | 0.004036 | 0.03221 | 10 |
| E02005391 | 2787 | 25 | 0.008970 | 0.02787 | 9 |
| E02005392 | 2915 | 29 | 0.010292 | 0.02915 | 10 |
| E02005393 | 2144 | 32 | 0.014925 | 0.02144 | 8 |
| E02005394 | 3101 | 49 | 0.015801 | 0.03101 | 6 |
| E02005395 | 2793 | 25 | 0.008951 | 0.02793 | 7 |
| E02005396 | 1744 | 18 | 0.010321 | 0.01744 | 7 |
| E02005397 | 3055 | 28 | 0.009165 | 0.03055 | 7 |
| E02005398 | 2010 | 20 | 0.009950 | 0.0201 | 9 |
| E02005399 | 2725 | 18 | 0.006606 | 0.02725 | 8 |
| E02005400 | 1548 | 30 | 0.019380 | 0.01548 | 6 |
| E02005401 | 1931 | 14 | 0.007250 | 0.01931 | 8 |
| E02005402 | 2176 | 18 | 0.008272 | 0.02176 | 8 |
| E02005403 | 1871 | 25 | 0.013362 | 0.01871 | 8 |
| E02005404 | 2571 | 23 | 0.008946 | 0.02571 | 6 |
| E02005405 | 2161 | 26 | 0.012031 | 0.02161 | 2 |
| E02005406 | 2615 | 31 | 0.011855 | 0.02615 | 5 |
| E02005407 | 3698 | 41 | 0.011087 | 0.03698 | 9 |
| E02005408 | 2294 | 15 | 0.006975 | 0.02294 | 7 |
| E02005409 | 3969 | 47 | 0.011842 | 0.03969 | 7 |
| E02005412 | 2908 | 55 | 0.018913 | 0.02908 | 8 |
| E02005414 | 2881 | 55 | 0.019091 | 0.02881 | 4 |
| E02005415 | 2774 | 52 | 0.018745 | 0.02774 | 4 |
| E02005416 | 2872 | 31 | 0.010794 | 0.02872 | 9 |
| E02005417 | 1917 | 27 | 0.014085 | 0.01917 | 4 |
| E02005418 | 3030 | 38 | 0.012541 | 0.0303 | 2 |
| E02005419 | 2682 | 37 | 0.013796 | 0.02682 | 4 |
| E02005420 | 2580 | 43 | 0.016667 | 0.0258 | 4 |
| E02005422 | 1684 | 21 | 0.012470 | 0.01684 | 4 |
| E02005423 | 2860 | 49 | 0.017832 | 0.0286 | 4 |
| E02005424 | 2749 | 25 | 0.009094 | 0.02749 | 7 |
| E02005425 | 1497 | 21 | 0.014028 | 0.01497 | 5 |
| E02005426 | 3235 | 51 | 0.015765 | 0.03235 | 3 |
| E02005427 | 1880 | 21 | 0.011702 | 0.0188 | 5 |
| E02005428 | 1826 | 73 | 0.040526 | 0.01826 | 1 |
| E02005429 | 1098 | 47 | 0.043716 | 0.01098 | 2 |
| E02005430 | 1546 | 14 | 0.009056 | 0.01546 | 4 |
| E02005431 | 1843 | 42 | 0.023332 | 0.01843 | 3 |
| E02005432 | 1552 | 29 | 0.018686 | 0.01552 | 4 |
| E02005433 | 1640 | 101 | 0.061585 | 0.0164 | 1 |
| E02005434 | 1998 | 42 | 0.021021 | 0.01998 | 5 |
| E02005435 | 2099 | 50 | 0.023821 | 0.02099 | 2 |
| E02005436 | 2062 | 27 | 0.013094 | 0.02062 | 4 |
| E02005437 | 1846 | 49 | 0.026544 | 0.01846 | 1 |
| E02005438 | 2069 | 65 | 0.031416 | 0.02069 | 1 |
| E02005439 | 3283 | 41 | 0.012489 | 0.03283 | 7 |
| E02005440 | 1287 | 26 | 0.020979 | 0.01287 | 1 |
| E02005441 | 1863 | 24 | 0.013419 | 0.01863 | 4 |
| E02005442 | 2074 | 35 | 0.016876 | 0.02074 | 1 |
| E02005443 | 2380 | 32 | 0.013445 | 0.0238 | 3 |
| E02005444 | 4369 | 47 | 0.010758 | 0.04369 | 6 |
| E02005445 | 4055 | 51 | 0.012577 | 0.04055 | 2 |
| E02005446 | 2889 | 34 | 0.011769 | 0.02889 | 5 |
| E02005447 | 2602 | 44 | 0.017294 | 0.02602 | 2 |
| E02005448 | 2270 | 32 | 0.014537 | 0.0227 | 2 |
| E02005449 | 2695 | 36 | 0.013729 | 0.02695 | 6 |
| E02005450 | 2437 | 40 | 0.016414 | 0.02437 | 7 |
| E02005451 | 2545 | 34 | 0.013360 | 0.02545 | 1 |
| E02005452 | 3524 | 40 | 0.011351 | 0.03524 | 6 |
| E02005453 | 3048 | 27 | 0.008858 | 0.03048 | 10 |
| E02005455 | 4424 | 46 | 0.010398 | 0.04424 | 9 |
| E02005456 | 3389 | 38 | 0.011213 | 0.03389 | 8 |
| E02005457 | 2506 | 36 | 0.014366 | 0.02506 | 8 |
| E02005458 | 1779 | 18 | 0.010118 | 0.01779 | 9 |
| E02005459 | 2179 | 26 | 0.012391 | 0.02179 | 4 |
| E02005460 | 1841 | 12 | 0.006518 | 0.01841 | 10 |
| E02005461 | 1443 | 27 | 0.019404 | 0.01443 | 8 |
| E02005462 | 2870 | 35 | 0.012195 | 0.0287 | 5 |
| E02005463 | 3035 | 22 | 0.007578 | 0.03035 | 9 |
| E02005464 | 2046 | 34 | 0.016618 | 0.02046 | 6 |
| E02005465 | 2227 | 33 | 0.014818 | 0.02227 | 6 |
| E02005466 | 1641 | 21 | 0.012797 | 0.01641 | 4 |
| E02005467 | 2102 | 53 | 0.025214 | 0.02102 | 6 |
| E02005468 | 1953 | 28 | 0.014337 | 0.01953 | 6 |
| E02005469 | 2134 | 22 | 0.010778 | 0.02134 | 6 |
| E02005470 | 4066 | 44 | 0.011067 | 0.04066 | 8 |
| E02005471 | 2733 | 49 | 0.017929 | 0.02733 | 5 |
| E02005472 | 2665 | 41 | 0.015760 | 0.02665 | 4 |
| E02005473 | 3094 | 33 | 0.010989 | 0.03094 | 8 |
| E02005474 | 1616 | 25 | 0.015470 | 0.01616 | 4 |
| E02005475 | 2054 | 29 | 0.014119 | 0.02054 | 7 |
| E02005476 | 1852 | 14 | 0.007559 | 0.01852 | 8 |
| E02005477 | 2422 | 33 | 0.013625 | 0.02422 | 9 |
| E02005478 | 3045 | 55 | 0.018062 | 0.03045 | 5 |
| E02005479 | 3248 | 53 | 0.016318 | 0.03248 | 8 |
| E02005480 | 2530 | 32 | 0.013043 | 0.0253 | 2 |
| E02005481 | 3814 | 65 | 0.017042 | 0.03814 | 5 |
| E02005482 | 2657 | 24 | 0.009033 | 0.02657 | 8 |
| E02005483 | 2149 | 32 | 0.014891 | 0.02149 | 6 |
| E02005484 | 1851 | 18 | 0.009724 | 0.01851 | 6 |
| E02005485 | 2770 | 33 | 0.011913 | 0.0277 | 10 |
| E02005486 | 2838 | 41 | 0.014447 | 0.02838 | 8 |
| E02005487 | 2778 | 29 | 0.010439 | 0.02778 | 10 |
| E02005488 | 2361 | 23 | 0.010165 | 0.02361 | 8 |
| E02005489 | 2192 | 17 | 0.007755 | 0.02192 | 9 |
| E02005490 | 2903 | 45 | 0.015501 | 0.02903 | 7 |
| E02005491 | 3229 | 47 | 0.014556 | 0.03229 | 9 |
| E02005492 | 2538 | 34 | 0.013396 | 0.02538 | 6 |
| E02005493 | 3095 | 35 | 0.011309 | 0.03095 | 7 |
| E02005494 | 2314 | 33 | 0.014261 | 0.02314 | 5 |
| E02005495 | 3124 | 53 | 0.017606 | 0.03124 | 1 |
| E02005496 | 1664 | 19 | 0.012019 | 0.01664 | 5 |
| E02005497 | 2400 | 52 | 0.021667 | 0.024 | 1 |
| E02005498 | 1925 | 21 | 0.010909 | 0.01925 | 6 |
| E02005499 | 2739 | 16 | 0.005842 | 0.02739 | 8 |
| E02005500 | 2188 | 29 | 0.013254 | 0.02188 | 8 |
| E02005501 | 2456 | 27 | 0.010993 | 0.02456 | 10 |
| E02005502 | 2893 | 38 | 0.013481 | 0.02893 | 6 |
| E02005612 | 2503 | 44 | 0.017579 | 0.02503 | 6 |
| E02005613 | 3177 | 43 | 0.013535 | 0.03177 | 4 |
| E02005614 | 4527 | 48 | 0.010603 | 0.04527 | 5 |
| E02005615 | 3244 | 55 | 0.016954 | 0.03244 | 4 |
| E02005616 | 2075 | 30 | 0.014458 | 0.02075 | 2 |
| E02005617 | 3775 | 35 | 0.009272 | 0.03775 | 1 |
| E02005619 | 1737 | 14 | 0.008060 | 0.01737 | 7 |
| E02005620 | 2664 | 27 | 0.010135 | 0.02664 | 10 |
| E02005621 | 2616 | 25 | 0.009557 | 0.02616 | 8 |
| E02005622 | 2950 | 18 | 0.006102 | 0.0295 | 10 |
| E02005623 | 3691 | 42 | 0.011379 | 0.03691 | 9 |
| E02005624 | 3627 | 41 | 0.011304 | 0.03627 | 8 |
| E02005625 | 2833 | 52 | 0.018355 | 0.02833 | 5 |
| E02005626 | 2177 | 35 | 0.016077 | 0.02177 | 3 |
| E02005627 | 2164 | 25 | 0.011553 | 0.02164 | 6 |
| E02005628 | 2212 | 25 | 0.011302 | 0.02212 | 8 |
| E02005629 | 1692 | 12 | 0.007092 | 0.01692 | 7 |
| E02005630 | 2843 | 9 | 0.003166 | 0.02843 | 9 |
| E02005631 | 3155 | 33 | 0.010777 | 0.03155 | 9 |
| E02005632 | 1835 | 25 | 0.013624 | 0.01835 | 8 |
| E02005633 | 2845 | 56 | 0.020035 | 0.02845 | 7 |
| E02005634 | 2913 | 53 | 0.018194 | 0.02913 | 6 |
| E02005635 | 2852 | 38 | 0.013324 | 0.02852 | 8 |
| E02005636 | 3809 | 42 | 0.011027 | 0.03809 | 5 |
| E02005637 | 2734 | 46 | 0.016825 | 0.02734 | 3 |
| E02005638 | 2983 | 39 | 0.013074 | 0.02983 | 10 |
| E02005639 | 1463 | 15 | 0.010253 | 0.01463 | 8 |
| E02005640 | 3760 | 45 | 0.011968 | 0.0376 | 7 |
| E02005641 | 2638 | 29 | 0.010993 | 0.02638 | 5 |
| E02005642 | 2733 | 36 | 0.013172 | 0.02733 | 8 |
| E02005643 | 2790 | 31 | 0.011111 | 0.0279 | 1 |
| E02005644 | 3031 | 52 | 0.017156 | 0.03031 | 3 |
| E02005645 | 4167 | 77 | 0.019678 | 0.04167 | 4 |
| E02005646 | 1981 | 32 | 0.016153 | 0.01981 | 9 |
| E02005647 | 2417 | 20 | 0.008275 | 0.02417 | 3 |
| E02005648 | 3338 | 39 | 0.011684 | 0.03338 | 9 |
| E02005649 | 5358 | 67 | 0.012691 | 0.05358 | 7 |
| E02005650 | 2101 | 28 | 0.013803 | 0.02101 | 7 |
| E02005651 | 1969 | 25 | 0.012697 | 0.01969 | 8 |
| E02005652 | 1980 | 39 | 0.019697 | 0.0198 | 6 |
| E02005653 | 2842 | 51 | 0.017945 | 0.02842 | 2 |
| E02005654 | 2668 | 40 | 0.015367 | 0.02668 | 2 |
| E02005655 | 1868 | 40 | 0.021413 | 0.01868 | 5 |
| E02005656 | 2306 | 36 | 0.015611 | 0.02306 | 2 |
| E02005657 | 1974 | 29 | 0.014691 | 0.01974 | 6 |
| E02005658 | 2177 | 33 | 0.015158 | 0.02177 | 2 |
| E02005659 | 1945 | 37 | 0.019023 | 0.01945 | 5 |
| E02005660 | 2150 | 35 | 0.016744 | 0.0215 | 2 |
| E02005661 | 2314 | 46 | 0.020311 | 0.02314 | 2 |
| E02005662 | 2344 | 31 | 0.013225 | 0.02344 | 5 |
| E02005663 | 1724 | 24 | 0.013921 | 0.01724 | 9 |
| E02005664 | 2121 | 53 | 0.024988 | 0.02121 | 3 |
| E02005665 | 2610 | 42 | 0.016092 | 0.0261 | 6 |
| E02005666 | 2989 | 61 | 0.021412 | 0.02989 | 1 |
| E02005667 | 2201 | 44 | 0.019991 | 0.02201 | 4 |
| E02005668 | 2028 | 43 | 0.021203 | 0.02028 | 7 |
| E02005669 | 1832 | 39 | 0.021288 | 0.01832 | 10 |
| E02005670 | 2865 | 46 | 0.016056 | 0.02865 | 2 |
| E02005671 | 2517 | 36 | 0.014303 | 0.02517 | 6 |
| E02005672 | 2674 | 38 | 0.014211 | 0.02674 | 3 |
| E02005673 | 2331 | 56 | 0.024024 | 0.02331 | 4 |
| E02005674 | 2877 | 64 | 0.022245 | 0.02877 | 2 |
| E02005675 | 2081 | 30 | 0.014897 | 0.02081 | 2 |
| E02005676 | 2080 | 28 | 0.013462 | 0.0208 | 4 |
| E02005677 | 3078 | 37 | 0.012021 | 0.03078 | 6 |
| E02005678 | 5156 | 73 | 0.014352 | 0.05156 | 7 |
| E02005679 | 2951 | 23 | 0.007794 | 0.02951 | 9 |
| E02005680 | 3335 | 26 | 0.007796 | 0.03335 | 10 |
| E02005681 | 2577 | 37 | 0.014358 | 0.02577 | 9 |
| E02005682 | 2212 | 24 | 0.010850 | 0.02212 | 10 |
| E02005683 | 4751 | 56 | 0.011787 | 0.04751 | 10 |
| E02005684 | 2427 | 25 | 0.010301 | 0.02427 | 9 |
| E02005685 | 3070 | 31 | 0.010098 | 0.0307 | 9 |
| E02005686 | 2189 | 24 | 0.010964 | 0.02189 | 9 |
| E02005687 | 1869 | 15 | 0.008026 | 0.01869 | 9 |
| E02005688 | 3040 | 34 | 0.011184 | 0.0304 | 10 |
| E02005689 | 3497 | 33 | 0.009437 | 0.03497 | 10 |
| E02005690 | 1519 | 21 | 0.013825 | 0.01519 | 9 |
| E02005691 | 1554 | 15 | 0.009653 | 0.01554 | 9 |
| E02005692 | 1824 | 29 | 0.016447 | 0.01824 | 6 |
| E02005693 | 2596 | 42 | 0.016179 | 0.02596 | 4 |
| E02005694 | 2358 | 33 | 0.013995 | 0.02358 | 9 |
| E02005695 | 3197 | 50 | 0.015640 | 0.03197 | 4 |
| E02005696 | 2053 | 30 | 0.016074 | 0.02053 | 2 |
| E02005697 | 2316 | 48 | 0.021157 | 0.02316 | 2 |
| E02005698 | 1795 | 41 | 0.022841 | 0.01795 | 4 |
| E02005699 | 2657 | 32 | 0.012044 | 0.02657 | 3 |
| E02005700 | 2908 | 25 | 0.008597 | 0.02908 | 9 |
| E02005701 | 3304 | 44 | 0.013317 | 0.03304 | 8 |
| E02005819 | 1855 | 23 | 0.012399 | 0.01855 | 3 |
| E02005820 | 1779 | 16 | 0.008994 | 0.01779 | 5 |
| E02005821 | 2123 | 30 | 0.014131 | 0.02123 | 2 |
| E02005822 | 3307 | 73 | 0.022074 | 0.03307 | 2 |
| E02005823 | 2729 | 58 | 0.021253 | 0.02729 | 1 |
| E02005824 | 3202 | 29 | 0.009057 | 0.03202 | 6 |
| E02005825 | 2531 | 36 | 0.014224 | 0.02531 | 3 |
| E02005826 | 2029 | 22 | 0.011336 | 0.02029 | 1 |
| E02005827 | 2978 | 35 | 0.012089 | 0.02978 | 5 |
| E02005828 | 2319 | 35 | 0.015955 | 0.02319 | 5 |
| E02005829 | 2092 | 24 | 0.011472 | 0.02092 | 5 |
| E02005830 | 1684 | 23 | 0.013658 | 0.01684 | 6 |
| E02005831 | 3566 | 47 | 0.013180 | 0.03566 | 5 |
| E02005832 | 3083 | 46 | 0.015894 | 0.03083 | 3 |
| E02005833 | 2409 | 33 | 0.013699 | 0.02409 | 8 |
| E02005834 | 2966 | 32 | 0.010789 | 0.02966 | 2 |
| E02005835 | 3614 | 46 | 0.012728 | 0.03614 | 4 |
| E02005836 | 2616 | 23 | 0.009174 | 0.02616 | 8 |
| E02005837 | 2479 | 24 | 0.009681 | 0.02479 | 6 |
| E02005838 | 2482 | 40 | 0.016116 | 0.02482 | 3 |
| E02005839 | 1946 | 32 | 0.016444 | 0.01946 | 5 |
| E02005840 | 3664 | 45 | 0.012282 | 0.03664 | 7 |
| E02005842 | 2630 | 52 | 0.019772 | 0.0263 | 4 |
| E02005843 | 2304 | 31 | 0.013889 | 0.02304 | 5 |
| E02005844 | 2863 | 36 | 0.012924 | 0.02863 | 4 |
| E02005846 | 2205 | 57 | 0.025850 | 0.02205 | 1 |
| E02005847 | 2285 | 36 | 0.015755 | 0.02285 | 4 |
| E02005848 | 2037 | 38 | 0.018655 | 0.02037 | 5 |
| E02005849 | 2674 | 35 | 0.013089 | 0.02674 | 5 |
| E02005850 | 2081 | 29 | 0.013936 | 0.02081 | 6 |
| E02005851 | 1955 | 15 | 0.008184 | 0.01955 | 2 |
| E02005852 | 2547 | 31 | 0.012564 | 0.02547 | 9 |
| E02005853 | 2800 | 41 | 0.014643 | 0.028 | 7 |
| E02005856 | 2206 | 19 | 0.008613 | 0.02206 | 10 |
| E02005857 | 1932 | 30 | 0.015528 | 0.01932 | 5 |
| E02005858 | 2642 | 22 | 0.008327 | 0.02642 | 7 |
| E02005859 | 3077 | 32 | 0.010725 | 0.03077 | 6 |
| E02005860 | 3108 | 29 | 0.009331 | 0.03108 | 7 |
| E02005861 | 1836 | 16 | 0.008715 | 0.01836 | 6 |
| E02005862 | 2473 | 40 | 0.016175 | 0.02473 | 6 |
| E02005863 | 2480 | 20 | 0.008065 | 0.0248 | 9 |
| E02005864 | 2744 | 32 | 0.011662 | 0.02744 | 10 |
| E02005865 | 2479 | 13 | 0.005647 | 0.02479 | 9 |
| E02005866 | 2963 | 36 | 0.012150 | 0.02963 | 7 |
| E02005868 | 2542 | 33 | 0.012982 | 0.02542 | 7 |
| E02005869 | 2102 | 43 | 0.020457 | 0.02102 | 3 |
| E02005870 | 2318 | 35 | 0.015099 | 0.02318 | 4 |
| E02005871 | 2219 | 29 | 0.013520 | 0.02219 | 9 |
| E02005872 | 2065 | 16 | 0.008232 | 0.02065 | 10 |
| E02005873 | 3308 | 35 | 0.010883 | 0.03308 | 10 |
| E02005874 | 2752 | 21 | 0.007631 | 0.02752 | 9 |
| E02005875 | 2846 | 45 | 0.015812 | 0.02846 | 7 |
| E02005876 | 3221 | 37 | 0.011487 | 0.03221 | 7 |
| E02005877 | 2426 | 34 | 0.014015 | 0.02426 | 6 |
| E02005878 | 3096 | 54 | 0.017442 | 0.03096 | 5 |
| E02005879 | 2342 | 29 | 0.012383 | 0.02342 | 4 |
| E02005880 | 2096 | 43 | 0.020515 | 0.02096 | 2 |
| E02005881 | 2103 | 37 | 0.017594 | 0.02103 | 2 |
| E02005882 | 2400 | 45 | 0.018750 | 0.024 | 6 |
| E02005883 | 2800 | 43 | 0.015357 | 0.028 | 2 |
| E02005884 | 1883 | 35 | 0.019649 | 0.01883 | 5 |
| E02005885 | 2773 | 47 | 0.017310 | 0.02773 | 2 |
| E02005886 | 3967 | 34 | 0.008571 | 0.03967 | 6 |
| E02005887 | 3471 | 57 | 0.016710 | 0.03471 | 2 |
| E02005888 | 2705 | 47 | 0.017745 | 0.02705 | 1 |
| E02005889 | 3479 | 62 | 0.018109 | 0.03479 | 3 |
| E02005890 | 2157 | 21 | 0.009736 | 0.02157 | 8 |
| E02005891 | 2002 | 42 | 0.021978 | 0.02002 | 1 |
| E02005892 | 4330 | 54 | 0.012702 | 0.0433 | 9 |
| E02005893 | 3737 | 64 | 0.017126 | 0.03737 | 3 |
| E02005894 | 3920 | 40 | 0.010459 | 0.0392 | 5 |
| E02005895 | 2123 | 25 | 0.011776 | 0.02123 | 7 |
| E02005896 | 1935 | 23 | 0.011886 | 0.01935 | 7 |
| E02005897 | 2614 | 40 | 0.015685 | 0.02614 | 7 |
| E02005898 | 3977 | 52 | 0.013327 | 0.03977 | 4 |
| E02005899 | 3663 | 59 | 0.016107 | 0.03663 | 3 |
| E02005900 | 2060 | 22 | 0.010680 | 0.0206 | 10 |
| E02005901 | 2999 | 53 | 0.017673 | 0.02999 | 5 |
| E02005902 | 2336 | 39 | 0.016695 | 0.02336 | 2 |
| E02005903 | 3042 | 56 | 0.018409 | 0.03042 | 6 |
| E02005904 | 2858 | 35 | 0.012246 | 0.02858 | 8 |
| E02005905 | 2063 | 33 | 0.015996 | 0.02063 | 8 |
| E02005906 | 2329 | 17 | 0.007299 | 0.02329 | 9 |
| E02005907 | 3137 | 21 | 0.006694 | 0.03137 | 10 |
| E02005908 | 2982 | 23 | 0.007713 | 0.02982 | 10 |
| E02005909 | 3004 | 15 | 0.005659 | 0.03004 | 10 |
| E02005910 | 3307 | 24 | 0.007257 | 0.03307 | 9 |
| E02005911 | 3037 | 31 | 0.010207 | 0.03037 | 10 |
| E02005912 | 3199 | 41 | 0.012817 | 0.03199 | 10 |
| E02005913 | 3002 | 29 | 0.009993 | 0.03002 | 10 |
| E02005914 | 2862 | 35 | 0.012928 | 0.02862 | 7 |
| E02005915 | 1592 | 19 | 0.011935 | 0.01592 | 9 |
| E02005916 | 2872 | 25 | 0.008705 | 0.02872 | 10 |
| E02005917 | 1768 | 19 | 0.010747 | 0.01768 | 10 |
| E02005918 | 1471 | 9 | 0.006118 | 0.01471 | 9 |
| E02005919 | 2166 | 10 | 0.004617 | 0.02166 | 9 |
| E02005920 | 2559 | 13 | 0.005080 | 0.02559 | 10 |
| E02006804 | 2632 | 64 | 0.021534 | 0.02632 | 5 |
| E02006815 | 3974 | 35 | 0.009969 | 0.03974 | 2 |
| E02006816 | 2879 | 39 | 0.015886 | 0.02879 | 10 |
| E02006817 | 2735 | 36 | 0.015195 | 0.02735 | 5 |
| E02006818 | 2791 | 52 | 0.022270 | 0.02791 | 8 |
| E02006819 | 2972 | 25 | 0.011563 | 0.02972 | 1 |
| E02006820 | 3511 | 37 | 0.012697 | 0.03511 | 8 |
| E02006827 | 2455 | 32 | 0.010760 | 0.02455 | 2 |
| E02006828 | 2435 | 29 | 0.014423 | 0.02435 | 7 |
| E02006834 | 2335 | 41 | 0.009176 | 0.02335 | 1 |
| E02006835 | 2162 | 40 | 0.016064 | 0.02162 | 6 |
| E02006850 | 2914 | 42 | 0.016306 | 0.02914 | 4 |
| E02006851 | 2974 | 27 | 0.009275 | 0.02974 | 3 |
| E02006862 | 2080 | 41 | 0.016439 | 0.0208 | 5 |
| E02006863 | 4468 | 29 | 0.008416 | 0.04468 | 7 |
| E02006864 | 2490 | 70 | 0.018201 | 0.0249 | 8 |
| E02006865 | 2637 | 45 | 0.013749 | 0.02637 | 8 |
| E02006866 | 2911 | 47 | 0.021739 | 0.02911 | 8 |
| E02006867 | 2494 | 55 | 0.014667 | 0.02494 | 9 |
| E02006872 | 3446 | 59 | 0.011732 | 0.03446 | 7 |
| E02006903 | 3846 | 50 | 0.015098 | 0.03846 | 4 |
| E02006904 | 3273 | 36 | 0.013667 | 0.03273 | 3 |
| E02006905 | 2162 | 20 | 0.010352 | 0.02162 | 2 |
| E02006906 | 3818 | 33 | 0.015179 | 0.03818 | 8 |
| E02006911 | 5029 | 38 | 0.011259 | 0.05029 | 10 |
| E02006919 | 3378 | 34 | 0.015511 | 0.03378 | 6 |
| E02006920 | 2634 | 25 | 0.010356 | 0.02634 | 5 |

**Table S6.** MHVI score (calculated from cumulative weekly GOV.UK positive case numbers and daily EMAS suspected severe illness case numbers) for cumulative period one (C1) between May 18^th^, 2020, and October 31^st^, 2020.

| **MSOA** | **GOV.UK** | **EMAS** | **MHVI** |
| --- | --- | --- | --- |
| E02002796 | 67 | 6 | 0.089552 |
| E02002797 | 68 | 10 | 0.147059 |
| E02002798 | 160 | 21 | 0.13125 |
| E02002799 | 97 | 13 | 0.134021 |
| E02002800 | 78 | 8 | 0.102564 |
| E02002801 | 65 | 12 | 0.184615 |
| E02002802 | 73 | 10 | 0.136986 |
| E02002803 | 127 | 14 | 0.110236 |
| E02002804 | 85 | 9 | 0.105882 |
| E02002805 | 38 | 4 | 0.105263 |
| E02002806 | 84 | 13 | 0.154762 |
| E02002807 | 95 | 14 | 0.147368 |
| E02002808 | 139 | 19 | 0.136691 |
| E02002809 | 75 | 20 | 0.266667 |
| E02002810 | 124 | 14 | 0.112903 |
| E02002811 | 146 | 23 | 0.157534 |
| E02002812 | 117 | 10 | 0.08547 |
| E02002813 | 143 | 27 | 0.188811 |
| E02002814 | 104 | 9 | 0.086538 |
| E02002815 | 96 | 14 | 0.145833 |
| E02002816 | 128 | 11 | 0.085938 |
| E02002817 | 83 | 12 | 0.144578 |
| E02002818 | 100 | 13 | 0.13 |
| E02002819 | 115 | 20 | 0.173913 |
| E02002820 | 119 | 13 | 0.109244 |
| E02002821 | 156 | 21 | 0.134615 |
| E02002822 | 83 | 12 | 0.144578 |
| E02002823 | 77 | 11 | 0.142857 |
| E02002824 | 151 | 25 | 0.165563 |
| E02002825 | 140 | 15 | 0.107143 |
| E02002826 | 128 | 5 | 0.039063 |
| E02002827 | 117 | 15 | 0.128205 |
| E02002828 | 259 | 18 | 0.069498 |
| E02002829 | 292 | 25 | 0.085616 |
| E02002830 | 185 | 22 | 0.118919 |
| E02002831 | 251 | 22 | 0.087649 |
| E02002832 | 463 | 24 | 0.051836 |
| E02002833 | 415 | 15 | 0.036145 |
| E02002834 | 317 | 16 | 0.050473 |
| E02002835 | 417 | 34 | 0.081535 |
| E02002836 | 512 | 24 | 0.046875 |
| E02002837 | 391 | 14 | 0.035806 |
| E02002838 | 106 | 24 | 0.226415 |
| E02002839 | 270 | 21 | 0.077778 |
| E02002842 | 148 | 12 | 0.081081 |
| E02002843 | 669 | 32 | 0.047833 |
| E02002844 | 420 | 27 | 0.064286 |
| E02002845 | 378 | 34 | 0.089947 |
| E02002846 | 101 | 5 | 0.049505 |
| E02002847 | 516 | 25 | 0.04845 |
| E02002848 | 469 | 23 | 0.049041 |
| E02002849 | 360 | 19 | 0.052778 |
| E02002851 | 294 | 16 | 0.054422 |
| E02002852 | 92 | 18 | 0.195652 |
| E02002853 | 483 | 31 | 0.064182 |
| E02002854 | 138 | 15 | 0.108696 |
| E02002855 | 151 | 5 | 0.033113 |
| E02002856 | 271 | 9 | 0.03321 |
| E02002857 | 97 | 11 | 0.113402 |
| E02002858 | 124 | 13 | 0.104839 |
| E02002860 | 139 | 11 | 0.079137 |
| E02002861 | 103 | 23 | 0.223301 |
| E02002862 | 107 | 21 | 0.196262 |
| E02002863 | 46 | 5 | 0.108696 |
| E02002864 | 35 | 12 | 0.342857 |
| E02002865 | 38 | 8 | 0.210526 |
| E02002866 | 29 | 5 | 0.172414 |
| E02002867 | 36 | 2 | 0.055556 |
| E02002868 | 91 | 8 | 0.087912 |
| E02002869 | 121 | 17 | 0.140496 |
| E02002871 | 139 | 23 | 0.165468 |
| E02002872 | 113 | 14 | 0.123894 |
| E02002873 | 155 | 17 | 0.109677 |
| E02002874 | 267 | 20 | 0.074906 |
| E02002875 | 151 | 18 | 0.119205 |
| E02002876 | 208 | 16 | 0.076923 |
| E02002877 | 136 | 14 | 0.102941 |
| E02002878 | 153 | 19 | 0.124183 |
| E02002879 | 206 | 30 | 0.145631 |
| E02002880 | 171 | 23 | 0.134503 |
| E02002881 | 101 | 10 | 0.09901 |
| E02002882 | 278 | 12 | 0.043165 |
| E02002883 | 137 | 19 | 0.138686 |
| E02002884 | 236 | 35 | 0.148305 |
| E02002885 | 305 | 27 | 0.088525 |
| E02002886 | 286 | 23 | 0.08042 |
| E02002887 | 145 | 22 | 0.151724 |
| E02002888 | 140 | 13 | 0.092857 |
| E02002889 | 1611 | 15 | 0.009311 |
| E02002890 | 922 | 15 | 0.016269 |
| E02002891 | 163 | 20 | 0.122699 |
| E02002892 | 111 | 11 | 0.099099 |
| E02002893 | 1009 | 20 | 0.019822 |
| E02002894 | 123 | 15 | 0.121951 |
| E02002895 | 1057 | 15 | 0.014191 |
| E02002896 | 203 | 15 | 0.073892 |
| E02002897 | 100 | 16 | 0.16 |
| E02002898 | 1189 | 61 | 0.051304 |
| E02002899 | 1160 | 13 | 0.011207 |
| E02002901 | 88 | 4 | 0.045455 |
| E02002902 | 454 | 22 | 0.048458 |
| E02002903 | 114 | 8 | 0.070175 |
| E02002904 | 129 | 29 | 0.224806 |
| E02004029 | 61 | 11 | 0.180328 |
| E02004030 | 42 | 4 | 0.095238 |
| E02004031 | 41 | 10 | 0.243902 |
| E02004032 | 95 | 11 | 0.115789 |
| E02004033 | 84 | 20 | 0.238095 |
| E02004034 | 53 | 10 | 0.188679 |
| E02004035 | 38 | 2 | 0.052632 |
| E02004036 | 148 | 12 | 0.081081 |
| E02004037 | 88 | 6 | 0.068182 |
| E02004038 | 76 | 11 | 0.144737 |
| E02004039 | 78 | 13 | 0.166667 |
| E02004040 | 109 | 13 | 0.119266 |
| E02004041 | 94 | 16 | 0.170213 |
| E02004043 | 80 | 9 | 0.1125 |
| E02004044 | 135 | 8 | 0.059259 |
| E02004045 | 152 | 11 | 0.072368 |
| E02004046 | 67 | 5 | 0.074627 |
| E02004047 | 71 | 11 | 0.15493 |
| E02004048 | 64 | 12 | 0.1875 |
| E02004049 | 91 | 14 | 0.153846 |
| E02004050 | 113 | 13 | 0.115044 |
| E02004051 | 163 | 12 | 0.07362 |
| E02004052 | 153 | 17 | 0.111111 |
| E02004053 | 62 | 11 | 0.177419 |
| E02004054 | 109 | 14 | 0.12844 |
| E02004055 | 99 | 13 | 0.131313 |
| E02004056 | 127 | 12 | 0.094488 |
| E02004057 | 68 | 10 | 0.147059 |
| E02004058 | 110 | 20 | 0.181818 |
| E02004059 | 98 | 15 | 0.153061 |
| E02004060 | 70 | 12 | 0.171429 |
| E02004061 | 81 | 10 | 0.123457 |
| E02004062 | 83 | 4 | 0.048193 |
| E02004063 | 75 | 11 | 0.146667 |
| E02004064 | 112 | 16 | 0.142857 |
| E02004065 | 73 | 12 | 0.164384 |
| E02004066 | 196 | 14 | 0.071429 |
| E02004067 | 57 | 8 | 0.140351 |
| E02004068 | 68 | 11 | 0.161765 |
| E02004069 | 45 | 7 | 0.155556 |
| E02004070 | 32 | 4 | 0.125 |
| E02004071 | 33 | 8 | 0.242424 |
| E02004072 | 82 | 11 | 0.134146 |
| E02004073 | 81 | 12 | 0.148148 |
| E02004074 | 11 | 6 | 0.545455 |
| E02004075 | 22 | 3 | 0.136364 |
| E02004076 | 36 | 11 | 0.305556 |
| E02004077 | 57 | 1 | 0.017544 |
| E02004078 | 89 | 13 | 0.146067 |
| E02004080 | 139 | 18 | 0.129496 |
| E02004081 | 51 | 11 | 0.215686 |
| E02004082 | 64 | 7 | 0.109375 |
| E02004083 | 125 | 9 | 0.072 |
| E02004084 | 66 | 10 | 0.151515 |
| E02004085 | 148 | 10 | 0.067568 |
| E02004086 | 73 | 9 | 0.123288 |
| E02004087 | 92 | 5 | 0.054348 |
| E02004088 | 108 | 3 | 0.027778 |
| E02004089 | 82 | 8 | 0.097561 |
| E02004090 | 97 | 6 | 0.061856 |
| E02004091 | 101 | 15 | 0.148515 |
| E02004092 | 112 | 6 | 0.053571 |
| E02004097 | 56 | 5 | 0.089286 |
| E02004098 | 64 | 12 | 0.1875 |
| E02004100 | 84 | 9 | 0.107143 |
| E02004102 | 70 | 19 | 0.271429 |
| E02004103 | 45 | 17 | 0.377778 |
| E02004104 | 58 | 9 | 0.155172 |
| E02004105 | 172 | 14 | 0.081395 |
| E02004106 | 110 | 8 | 0.072727 |
| E02004108 | 149 | 15 | 0.100671 |
| E02004109 | 123 | 5 | 0.04065 |
| E02004110 | 117 | 12 | 0.102564 |
| E02004111 | 51 | 7 | 0.137255 |
| E02004112 | 65 | 4 | 0.061538 |
| E02004113 | 83 | 15 | 0.180723 |
| E02004114 | 55 | 13 | 0.236364 |
| E02004115 | 112 | 10 | 0.089286 |
| E02004116 | 88 | 19 | 0.215909 |
| E02004117 | 109 | 15 | 0.137615 |
| E02004118 | 95 | 7 | 0.073684 |
| E02004119 | 159 | 9 | 0.056604 |
| E02004120 | 147 | 10 | 0.068027 |
| E02004121 | 122 | 12 | 0.098361 |
| E02004122 | 84 | 1 | 0.011905 |
| E02004123 | 59 | 5 | 0.084746 |
| E02004124 | 90 | 10 | 0.111111 |
| E02004125 | 100 | 12 | 0.12 |
| E02004126 | 81 | 9 | 0.111111 |
| E02004128 | 135 | 10 | 0.074074 |
| E02005334 | 119 | 10 | 0.084034 |
| E02005335 | 95 | 6 | 0.063158 |
| E02005336 | 156 | 11 | 0.070513 |
| E02005337 | 155 | 19 | 0.122581 |
| E02005338 | 140 | 5 | 0.035714 |
| E02005339 | 122 | 7 | 0.057377 |
| E02005340 | 84 | 12 | 0.142857 |
| E02005341 | 115 | 5 | 0.043478 |
| E02005342 | 64 | 6 | 0.09375 |
| E02005343 | 87 | 1 | 0.011494 |
| E02005344 | 54 | 4 | 0.074074 |
| E02005345 | 58 | 6 | 0.103448 |
| E02005346 | 230 | 21 | 0.091304 |
| E02005347 | 773 | 27 | 0.034929 |
| E02005348 | 80 | 5 | 0.0625 |
| E02005349 | 81 | 7 | 0.08642 |
| E02005350 | 67 | 5 | 0.074627 |
| E02005351 | 514 | 17 | 0.033074 |
| E02005352 | 64 | 5 | 0.078125 |
| E02005353 | 166 | 10 | 0.060241 |
| E02005354 | 145 | 10 | 0.068966 |
| E02005355 | 83 | 4 | 0.048193 |
| E02005356 | 105 | 12 | 0.114286 |
| E02005357 | 128 | 11 | 0.085938 |
| E02005358 | 160 | 10 | 0.0625 |
| E02005359 | 130 | 11 | 0.084615 |
| E02005360 | 38 | 6 | 0.157895 |
| E02005361 | 76 | 10 | 0.131579 |
| E02005362 | 132 | 15 | 0.113636 |
| E02005363 | 187 | 15 | 0.080214 |
| E02005364 | 94 | 10 | 0.106383 |
| E02005365 | 193 | 15 | 0.07772 |
| E02005366 | 92 | 11 | 0.119565 |
| E02005368 | 56 | 5 | 0.089286 |
| E02005369 | 116 | 14 | 0.12069 |
| E02005370 | 65 | 2 | 0.030769 |
| E02005371 | 94 | 9 | 0.095745 |
| E02005372 | 41 | 2 | 0.04878 |
| E02005373 | 55 | 11 | 0.2 |
| E02005374 | 49 | 9 | 0.183673 |
| E02005375 | 94 | 16 | 0.170213 |
| E02005376 | 62 | 11 | 0.177419 |
| E02005377 | 67 | 8 | 0.119403 |
| E02005378 | 66 | 4 | 0.060606 |
| E02005379 | 49 | 3 | 0.061224 |
| E02005380 | 49 | 1 | 0.020408 |
| E02005381 | 80 | 4 | 0.05 |
| E02005382 | 86 | 7 | 0.081395 |
| E02005383 | 63 | 10 | 0.15873 |
| E02005384 | 34 | 2 | 0.058824 |
| E02005385 | 57 | 6 | 0.105263 |
| E02005386 | 131 | 14 | 0.10687 |
| E02005387 | 83 | 7 | 0.084337 |
| E02005388 | 66 | 4 | 0.060606 |
| E02005389 | 46 | 6 | 0.130435 |
| E02005390 | 56 | 5 | 0.089286 |
| E02005391 | 88 | 5 | 0.056818 |
| E02005392 | 62 | 8 | 0.129032 |
| E02005393 | 52 | 10 | 0.192308 |
| E02005394 | 77 | 20 | 0.25974 |
| E02005395 | 56 | 9 | 0.160714 |
| E02005396 | 37 | 5 | 0.135135 |
| E02005397 | 88 | 10 | 0.113636 |
| E02005398 | 76 | 6 | 0.078947 |
| E02005399 | 43 | 4 | 0.093023 |
| E02005400 | 24 | 7 | 0.291667 |
| E02005401 | 47 | 3 | 0.06383 |
| E02005402 | 52 | 6 | 0.115385 |
| E02005403 | 57 | 10 | 0.175439 |
| E02005404 | 56 | 11 | 0.196429 |
| E02005405 | 36 | 7 | 0.194444 |
| E02005406 | 38 | 13 | 0.342105 |
| E02005407 | 72 | 12 | 0.166667 |
| E02005408 | 43 | 5 | 0.116279 |
| E02005409 | 105 | 14 | 0.133333 |
| E02005412 | 249 | 23 | 0.092369 |
| E02005414 | 130 | 16 | 0.123077 |
| E02005415 | 97 | 14 | 0.14433 |
| E02005416 | 84 | 6 | 0.071429 |
| E02005417 | 19 | 9 | 0.473684 |
| E02005418 | 83 | 17 | 0.204819 |
| E02005419 | 76 | 12 | 0.157895 |
| E02005420 | 62 | 14 | 0.225806 |
| E02005422 | 35 | 6 | 0.171429 |
| E02005423 | 88 | 16 | 0.181818 |
| E02005424 | 93 | 7 | 0.075269 |
| E02005425 | 19 | 8 | 0.421053 |
| E02005426 | 67 | 10 | 0.149254 |
| E02005427 | 41 | 5 | 0.121951 |
| E02005428 | 74 | 23 | 0.310811 |
| E02005429 | 81 | 17 | 0.209877 |
| E02005430 | 36 | 6 | 0.166667 |
| E02005431 | 35 | 8 | 0.228571 |
| E02005432 | 19 | 9 | 0.473684 |
| E02005433 | 69 | 34 | 0.492754 |
| E02005434 | 25 | 12 | 0.48 |
| E02005435 | 54 | 18 | 0.333333 |
| E02005436 | 30 | 10 | 0.333333 |
| E02005437 | 35 | 15 | 0.428571 |
| E02005438 | 66 | 30 | 0.454545 |
| E02005439 | 68 | 9 | 0.132353 |
| E02005440 | 19 | 10 | 0.526316 |
| E02005441 | 22 | 14 | 0.636364 |
| E02005442 | 45 | 9 | 0.2 |
| E02005443 | 43 | 9 | 0.209302 |
| E02005444 | 134 | 8 | 0.059701 |
| E02005445 | 156 | 19 | 0.121795 |
| E02005446 | 354 | 15 | 0.042373 |
| E02005447 | 128 | 12 | 0.09375 |
| E02005448 | 28 | 13 | 0.464286 |
| E02005449 | 150 | 11 | 0.073333 |
| E02005450 | 63 | 6 | 0.095238 |
| E02005451 | 60 | 8 | 0.133333 |
| E02005452 | 118 | 14 | 0.118644 |
| E02005453 | 68 | 8 | 0.117647 |
| E02005455 | 102 | 14 | 0.137255 |
| E02005456 | 82 | 13 | 0.158537 |
| E02005457 | 57 | 9 | 0.157895 |
| E02005458 | 17 | 6 | 0.352941 |
| E02005459 | 43 | 6 | 0.139535 |
| E02005460 | 37 | 4 | 0.108108 |
| E02005461 | 26 | 9 | 0.346154 |
| E02005462 | 41 | 6 | 0.146341 |
| E02005463 | 56 | 6 | 0.107143 |
| E02005464 | 56 | 12 | 0.214286 |
| E02005465 | 27 | 12 | 0.444444 |
| E02005466 | 26 | 7 | 0.269231 |
| E02005467 | 33 | 20 | 0.606061 |
| E02005468 | 22 | 11 | 0.5 |
| E02005469 | 25 | 14 | 0.56 |
| E02005470 | 128 | 15 | 0.117188 |
| E02005471 | 99 | 18 | 0.181818 |
| E02005472 | 56 | 16 | 0.285714 |
| E02005473 | 117 | 21 | 0.179487 |
| E02005474 | 35 | 6 | 0.171429 |
| E02005475 | 23 | 8 | 0.347826 |
| E02005476 | 53 | 2 | 0.037736 |
| E02005477 | 63 | 3 | 0.047619 |
| E02005478 | 53 | 13 | 0.245283 |
| E02005479 | 83 | 9 | 0.108434 |
| E02005480 | 58 | 14 | 0.241379 |
| E02005481 | 90 | 24 | 0.266667 |
| E02005482 | 56 | 3 | 0.053571 |
| E02005483 | 33 | 10 | 0.30303 |
| E02005484 | 29 | 5 | 0.172414 |
| E02005485 | 45 | 13 | 0.288889 |
| E02005486 | 50 | 18 | 0.36 |
| E02005487 | 27 | 7 | 0.259259 |
| E02005488 | 85 | 10 | 0.117647 |
| E02005489 | 65 | 6 | 0.092308 |
| E02005490 | 38 | 11 | 0.289474 |
| E02005491 | 37 | 18 | 0.486486 |
| E02005492 | 73 | 5 | 0.068493 |
| E02005493 | 82 | 13 | 0.158537 |
| E02005494 | 27 | 9 | 0.333333 |
| E02005495 | 101 | 17 | 0.168317 |
| E02005496 | 36 | 8 | 0.222222 |
| E02005497 | 43 | 9 | 0.209302 |
| E02005498 | 32 | 2 | 0.0625 |
| E02005499 | 63 | 4 | 0.063492 |
| E02005500 | 53 | 8 | 0.150943 |
| E02005501 | 52 | 7 | 0.134615 |
| E02005502 | 82 | 14 | 0.170732 |
| E02005612 | 41 | 18 | 0.439024 |
| E02005613 | 58 | 10 | 0.172414 |
| E02005614 | 113 | 16 | 0.141593 |
| E02005615 | 58 | 19 | 0.327586 |
| E02005616 | 33 | 11 | 0.333333 |
| E02005617 | 72 | 12 | 0.166667 |
| E02005619 | 23 | 1 | 0.043478 |
| E02005620 | 65 | 15 | 0.230769 |
| E02005621 | 32 | 10 | 0.3125 |
| E02005622 | 47 | 5 | 0.106383 |
| E02005623 | 73 | 10 | 0.136986 |
| E02005624 | 50 | 9 | 0.18 |
| E02005625 | 50 | 20 | 0.4 |
| E02005626 | 48 | 16 | 0.333333 |
| E02005627 | 31 | 8 | 0.258065 |
| E02005628 | 30 | 7 | 0.233333 |
| E02005629 | 17 | 3 | 0.176471 |
| E02005630 | 69 | 5 | 0.072464 |
| E02005631 | 50 | 11 | 0.22 |
| E02005632 | 37 | 6 | 0.162162 |
| E02005633 | 85 | 15 | 0.176471 |
| E02005634 | 73 | 18 | 0.246575 |
| E02005635 | 65 | 11 | 0.169231 |
| E02005636 | 76 | 15 | 0.197368 |
| E02005637 | 73 | 18 | 0.246575 |
| E02005638 | 76 | 8 | 0.105263 |
| E02005639 | 39 | 5 | 0.128205 |
| E02005640 | 96 | 12 | 0.125 |
| E02005641 | 49 | 12 | 0.244898 |
| E02005642 | 63 | 9 | 0.142857 |
| E02005643 | 48 | 13 | 0.270833 |
| E02005644 | 54 | 25 | 0.462963 |
| E02005645 | 136 | 28 | 0.205882 |
| E02005646 | 29 | 12 | 0.413793 |
| E02005647 | 46 | 9 | 0.195652 |
| E02005648 | 81 | 13 | 0.160494 |
| E02005649 | 127 | 22 | 0.173228 |
| E02005650 | 64 | 7 | 0.109375 |
| E02005651 | 72 | 7 | 0.097222 |
| E02005652 | 58 | 9 | 0.155172 |
| E02005653 | 118 | 10 | 0.084746 |
| E02005654 | 65 | 9 | 0.138462 |
| E02005655 | 61 | 14 | 0.229508 |
| E02005656 | 91 | 14 | 0.153846 |
| E02005657 | 38 | 17 | 0.447368 |
| E02005658 | 75 | 7 | 0.093333 |
| E02005659 | 65 | 17 | 0.261538 |
| E02005660 | 75 | 17 | 0.226667 |
| E02005661 | 86 | 19 | 0.22093 |
| E02005662 | 70 | 12 | 0.171429 |
| E02005663 | 55 | 4 | 0.072727 |
| E02005664 | 57 | 14 | 0.245614 |
| E02005665 | 88 | 14 | 0.159091 |
| E02005666 | 88 | 26 | 0.295455 |
| E02005667 | 43 | 14 | 0.325581 |
| E02005668 | 51 | 9 | 0.176471 |
| E02005669 | 66 | 13 | 0.19697 |
| E02005670 | 130 | 16 | 0.123077 |
| E02005671 | 117 | 10 | 0.08547 |
| E02005672 | 104 | 14 | 0.134615 |
| E02005673 | 88 | 21 | 0.238636 |
| E02005674 | 159 | 17 | 0.106918 |
| E02005675 | 31 | 9 | 0.290323 |
| E02005676 | 61 | 15 | 0.245902 |
| E02005677 | 135 | 12 | 0.088889 |
| E02005678 | 90 | 19 | 0.211111 |
| E02005679 | 48 | 8 | 0.166667 |
| E02005680 | 67 | 4 | 0.059701 |
| E02005681 | 52 | 7 | 0.134615 |
| E02005682 | 42 | 10 | 0.238095 |
| E02005683 | 85 | 18 | 0.211765 |
| E02005684 | 31 | 8 | 0.258065 |
| E02005685 | 41 | 5 | 0.121951 |
| E02005686 | 53 | 4 | 0.075472 |
| E02005687 | 27 | 5 | 0.185185 |
| E02005688 | 34 | 5 | 0.147059 |
| E02005689 | 54 | 7 | 0.12963 |
| E02005690 | 24 | 8 | 0.333333 |
| E02005691 | 18 | 3 | 0.166667 |
| E02005692 | 22 | 10 | 0.454545 |
| E02005693 | 75 | 13 | 0.173333 |
| E02005694 | 58 | 11 | 0.189655 |
| E02005695 | 133 | 18 | 0.135338 |
| E02005696 | 25 | 9 | 0.36 |
| E02005697 | 66 | 21 | 0.318182 |
| E02005698 | 49 | 12 | 0.244898 |
| E02005699 | 67 | 12 | 0.179104 |
| E02005700 | 65 | 8 | 0.123077 |
| E02005701 | 60 | 17 | 0.283333 |
| E02005819 | 47 | 8 | 0.170213 |
| E02005820 | 57 | 4 | 0.070175 |
| E02005821 | 48 | 10 | 0.208333 |
| E02005822 | 119 | 27 | 0.226891 |
| E02005823 | 105 | 14 | 0.133333 |
| E02005824 | 91 | 5 | 0.054945 |
| E02005825 | 113 | 11 | 0.097345 |
| E02005826 | 46 | 6 | 0.130435 |
| E02005827 | 106 | 7 | 0.066038 |
| E02005828 | 103 | 13 | 0.126214 |
| E02005829 | 95 | 7 | 0.073684 |
| E02005830 | 61 | 8 | 0.131148 |
| E02005831 | 196 | 12 | 0.061224 |
| E02005832 | 203 | 24 | 0.118227 |
| E02005833 | 116 | 11 | 0.094828 |
| E02005834 | 189 | 10 | 0.05291 |
| E02005835 | 241 | 12 | 0.049793 |
| E02005836 | 93 | 9 | 0.096774 |
| E02005837 | 67 | 6 | 0.089552 |
| E02005838 | 109 | 7 | 0.06422 |
| E02005839 | 95 | 7 | 0.073684 |
| E02005840 | 157 | 19 | 0.121019 |
| E02005842 | 75 | 16 | 0.213333 |
| E02005843 | 90 | 12 | 0.133333 |
| E02005844 | 108 | 9 | 0.083333 |
| E02005846 | 82 | 21 | 0.256098 |
| E02005847 | 120 | 15 | 0.125 |
| E02005848 | 70 | 12 | 0.171429 |
| E02005849 | 53 | 8 | 0.150943 |
| E02005850 | 101 | 4 | 0.039604 |
| E02005852 | 119 | 14 | 0.117647 |
| E02005853 | 146 | 23 | 0.157534 |
| E02005856 | 84 | 9 | 0.107143 |
| E02005857 | 103 | 16 | 0.15534 |
| E02005858 | 375 | 12 | 0.032 |
| E02005859 | 122 | 11 | 0.090164 |
| E02005860 | 200 | 8 | 0.04 |
| E02005861 | 74 | 9 | 0.121622 |
| E02005862 | 86 | 13 | 0.151163 |
| E02005863 | 127 | 7 | 0.055118 |
| E02005864 | 112 | 8 | 0.071429 |
| E02005865 | 102 | 2 | 0.019608 |
| E02005866 | 135 | 20 | 0.148148 |
| E02005868 | 170 | 7 | 0.041176 |
| E02005869 | 114 | 14 | 0.122807 |
| E02005870 | 130 | 10 | 0.076923 |
| E02005871 | 123 | 9 | 0.073171 |
| E02005872 | 71 | 4 | 0.056338 |
| E02005873 | 203 | 10 | 0.049261 |
| E02005874 | 103 | 7 | 0.067961 |
| E02005875 | 125 | 13 | 0.104 |
| E02005876 | 156 | 8 | 0.051282 |
| E02005877 | 142 | 9 | 0.06338 |
| E02005878 | 182 | 21 | 0.115385 |
| E02005879 | 77 | 11 | 0.142857 |
| E02005880 | 57 | 17 | 0.298246 |
| E02005881 | 57 | 17 | 0.298246 |
| E02005882 | 110 | 12 | 0.109091 |
| E02005883 | 133 | 10 | 0.075188 |
| E02005884 | 70 | 11 | 0.157143 |
| E02005885 | 139 | 11 | 0.079137 |
| E02005886 | 133 | 9 | 0.067669 |
| E02005887 | 153 | 15 | 0.098039 |
| E02005888 | 103 | 10 | 0.097087 |
| E02005889 | 113 | 16 | 0.141593 |
| E02005890 | 59 | 9 | 0.152542 |
| E02005891 | 69 | 22 | 0.318841 |
| E02005892 | 177 | 13 | 0.073446 |
| E02005893 | 193 | 25 | 0.129534 |
| E02005894 | 166 | 12 | 0.072289 |
| E02005895 | 48 | 5 | 0.104167 |
| E02005896 | 49 | 4 | 0.081633 |
| E02005897 | 120 | 14 | 0.116667 |
| E02005898 | 146 | 11 | 0.075342 |
| E02005899 | 111 | 16 | 0.144144 |
| E02005900 | 98 | 7 | 0.071429 |
| E02005901 | 72 | 15 | 0.208333 |
| E02005902 | 76 | 10 | 0.131579 |
| E02005903 | 73 | 19 | 0.260274 |
| E02005904 | 100 | 12 | 0.12 |
| E02005905 | 242 | 17 | 0.070248 |
| E02005906 | 148 | 6 | 0.040541 |
| E02005907 | 82 | 8 | 0.097561 |
| E02005908 | 123 | 9 | 0.073171 |
| E02005909 | 163 | 6 | 0.03681 |
| E02005910 | 297 | 12 | 0.040404 |
| E02005911 | 160 | 9 | 0.05625 |
| E02005912 | 176 | 11 | 0.0625 |
| E02005913 | 154 | 14 | 0.090909 |
| E02005914 | 184 | 11 | 0.059783 |
| E02005915 | 44 | 6 | 0.136364 |
| E02005916 | 194 | 7 | 0.036082 |
| E02005917 | 111 | 3 | 0.027027 |
| E02005918 | 62 | 3 | 0.048387 |
| E02005919 | 120 | 1 | 0.008333 |
| E02005920 | 76 | 3 | 0.039474 |
| E02006804 | 136 | 11 | 0.080882 |
| E02006815 | 244 | 21 | 0.086066 |
| E02006816 | 145 | 13 | 0.089655 |
| E02006817 | 224 | 14 | 0.0625 |
| E02006818 | 142 | 17 | 0.119718 |
| E02006819 | 103 | 20 | 0.194175 |
| E02006820 | 145 | 7 | 0.048276 |
| E02006827 | 118 | 18 | 0.152542 |
| E02006828 | 85 | 7 | 0.082353 |
| E02006834 | 102 | 21 | 0.205882 |
| E02006835 | 110 | 10 | 0.090909 |
| E02006850 | 374 | 15 | 0.040107 |
| E02006851 | 313 | 11 | 0.035144 |
| E02006862 | 38 | 7 | 0.184211 |
| E02006863 | 92 | 10 | 0.108696 |
| E02006864 | 64 | 12 | 0.1875 |
| E02006865 | 65 | 6 | 0.092308 |
| E02006866 | 37 | 11 | 0.297297 |
| E02006867 | 39 | 12 | 0.307692 |
| E02006872 | 129 | 8 | 0.062016 |
| E02006903 | 197 | 21 | 0.106599 |
| E02006904 | 489 | 21 | 0.042945 |
| E02006905 | 121 | 16 | 0.132231 |
| E02006906 | 260 | 12 | 0.046154 |
| E02006911 | 534 | 24 | 0.044944 |
| E02006919 | 111 | 8 | 0.072072 |
| E02006920 | 11 | 77 | 0.142857 |

**Table S7.** MHVI score (calculated from cumulative weekly GOV.UK positive case numbers and daily EMAS suspected severe illness case numbers) for cumulative period two (C2) between May 18^th^, 2020, and April 17^th^, 2021.

| **MSOA** | **GOV.UK** | **EMAS** | **MHVI** |
| --- | --- | --- | --- |
| E02002796 | 267 | 13 | 0.048689 |
| E02002797 | 338 | 22 | 0.065089 |
| E02002798 | 647 | 29 | 0.044822 |
| E02002799 | 467 | 25 | 0.053533 |
| E02002800 | 408 | 13 | 0.031863 |
| E02002801 | 462 | 35 | 0.075758 |
| E02002802 | 563 | 23 | 0.040853 |
| E02002803 | 498 | 29 | 0.058233 |
| E02002804 | 638 | 33 | 0.051724 |
| E02002805 | 250 | 11 | 0.044 |
| E02002806 | 456 | 28 | 0.061404 |
| E02002807 | 582 | 33 | 0.056701 |
| E02002808 | 915 | 40 | 0.043716 |
| E02002809 | 424 | 42 | 0.099057 |
| E02002810 | 685 | 44 | 0.064234 |
| E02002811 | 1061 | 58 | 0.054665 |
| E02002812 | 771 | 32 | 0.041505 |
| E02002813 | 1149 | 73 | 0.063534 |
| E02002814 | 557 | 26 | 0.046679 |
| E02002815 | 795 | 35 | 0.044025 |
| E02002816 | 776 | 30 | 0.03866 |
| E02002817 | 561 | 35 | 0.062389 |
| E02002818 | 682 | 28 | 0.041056 |
| E02002819 | 613 | 38 | 0.06199 |
| E02002820 | 694 | 35 | 0.050432 |
| E02002821 | 923 | 45 | 0.048754 |
| E02002822 | 603 | 34 | 0.056385 |
| E02002823 | 547 | 39 | 0.071298 |
| E02002824 | 907 | 46 | 0.050717 |
| E02002825 | 610 | 32 | 0.052459 |
| E02002826 | 435 | 14 | 0.032184 |
| E02002827 | 634 | 30 | 0.047319 |
| E02002828 | 790 | 41 | 0.051899 |
| E02002829 | 1262 | 47 | 0.037242 |
| E02002830 | 1019 | 58 | 0.056919 |
| E02002831 | 840 | 47 | 0.055952 |
| E02002832 | 1244 | 57 | 0.04582 |
| E02002833 | 1000 | 38 | 0.038 |
| E02002834 | 1194 | 35 | 0.029313 |
| E02002835 | 1715 | 75 | 0.043732 |
| E02002836 | 1152 | 50 | 0.043403 |
| E02002837 | 1049 | 27 | 0.025739 |
| E02002838 | 686 | 42 | 0.061224 |
| E02002839 | 940 | 47 | 0.05 |
| E02002842 | 659 | 22 | 0.033384 |
| E02002843 | 1702 | 66 | 0.038778 |
| E02002844 | 1363 | 59 | 0.043287 |
| E02002845 | 1183 | 59 | 0.049873 |
| E02002846 | 565 | 14 | 0.024779 |
| E02002847 | 1376 | 50 | 0.036337 |
| E02002848 | 1319 | 51 | 0.038666 |
| E02002849 | 1253 | 31 | 0.024741 |
| E02002851 | 1062 | 42 | 0.039548 |
| E02002852 | 629 | 37 | 0.058824 |
| E02002853 | 1585 | 72 | 0.045426 |
| E02002854 | 713 | 33 | 0.046283 |
| E02002855 | 648 | 16 | 0.024691 |
| E02002856 | 895 | 29 | 0.032402 |
| E02002857 | 566 | 29 | 0.051237 |
| E02002858 | 535 | 34 | 0.063551 |
| E02002860 | 690 | 29 | 0.042029 |
| E02002861 | 587 | 39 | 0.06644 |
| E02002862 | 654 | 38 | 0.058104 |
| E02002863 | 465 | 10 | 0.021505 |
| E02002864 | 344 | 22 | 0.063953 |
| E02002865 | 237 | 15 | 0.063291 |
| E02002866 | 227 | 16 | 0.070485 |
| E02002867 | 266 | 9 | 0.033835 |
| E02002868 | 444 | 19 | 0.042793 |
| E02002869 | 627 | 37 | 0.059011 |
| E02002871 | 655 | 37 | 0.056489 |
| E02002872 | 699 | 36 | 0.051502 |
| E02002873 | 721 | 37 | 0.051318 |
| E02002874 | 934 | 43 | 0.046039 |
| E02002875 | 628 | 34 | 0.05414 |
| E02002876 | 627 | 35 | 0.055821 |
| E02002877 | 494 | 30 | 0.060729 |
| E02002878 | 754 | 37 | 0.049072 |
| E02002879 | 783 | 49 | 0.06258 |
| E02002880 | 742 | 38 | 0.051213 |
| E02002881 | 559 | 22 | 0.039356 |
| E02002882 | 689 | 25 | 0.036284 |
| E02002883 | 560 | 32 | 0.057143 |
| E02002884 | 806 | 68 | 0.084367 |
| E02002885 | 857 | 47 | 0.054842 |
| E02002886 | 929 | 43 | 0.046286 |
| E02002887 | 540 | 51 | 0.094444 |
| E02002888 | 599 | 31 | 0.051753 |
| E02002889 | 1908 | 23 | 0.012055 |
| E02002890 | 1325 | 27 | 0.020377 |
| E02002891 | 637 | 37 | 0.058085 |
| E02002892 | 474 | 28 | 0.059072 |
| E02002893 | 1593 | 42 | 0.026365 |
| E02002894 | 479 | 28 | 0.058455 |
| E02002895 | 1351 | 25 | 0.018505 |
| E02002896 | 725 | 48 | 0.066207 |
| E02002897 | 447 | 30 | 0.067114 |
| E02002898 | 1546 | 87 | 0.056274 |
| E02002899 | 1369 | 22 | 0.01607 |
| E02002901 | 454 | 23 | 0.050661 |
| E02002902 | 947 | 40 | 0.042239 |
| E02002903 | 464 | 24 | 0.051724 |
| E02002904 | 566 | 45 | 0.079505 |
| E02004029 | 486 | 31 | 0.063786 |
| E02004030 | 243 | 13 | 0.053498 |
| E02004031 | 551 | 37 | 0.067151 |
| E02004032 | 671 | 29 | 0.043219 |
| E02004033 | 672 | 45 | 0.066964 |
| E02004034 | 436 | 35 | 0.080275 |
| E02004035 | 319 | 13 | 0.040752 |
| E02004036 | 788 | 49 | 0.062183 |
| E02004037 | 441 | 14 | 0.031746 |
| E02004038 | 433 | 30 | 0.069284 |
| E02004039 | 461 | 24 | 0.052061 |
| E02004040 | 648 | 35 | 0.054012 |
| E02004041 | 535 | 33 | 0.061682 |
| E02004043 | 511 | 23 | 0.04501 |
| E02004044 | 502 | 21 | 0.041833 |
| E02004045 | 608 | 20 | 0.032895 |
| E02004046 | 384 | 17 | 0.044271 |
| E02004047 | 447 | 22 | 0.049217 |
| E02004048 | 404 | 21 | 0.05198 |
| E02004049 | 569 | 29 | 0.050967 |
| E02004050 | 674 | 28 | 0.041543 |
| E02004051 | 831 | 39 | 0.046931 |
| E02004052 | 627 | 44 | 0.070175 |
| E02004053 | 473 | 26 | 0.054968 |
| E02004054 | 664 | 54 | 0.081325 |
| E02004055 | 408 | 23 | 0.056373 |
| E02004056 | 527 | 24 | 0.045541 |
| E02004057 | 403 | 19 | 0.047146 |
| E02004058 | 439 | 38 | 0.08656 |
| E02004059 | 471 | 29 | 0.061571 |
| E02004060 | 448 | 30 | 0.066964 |
| E02004061 | 419 | 28 | 0.066826 |
| E02004062 | 348 | 17 | 0.048851 |
| E02004063 | 389 | 27 | 0.069409 |
| E02004064 | 519 | 33 | 0.063584 |
| E02004065 | 348 | 20 | 0.057471 |
| E02004066 | 706 | 32 | 0.045326 |
| E02004067 | 338 | 23 | 0.068047 |
| E02004068 | 254 | 16 | 0.062992 |
| E02004069 | 186 | 14 | 0.075269 |
| E02004070 | 185 | 13 | 0.07027 |
| E02004071 | 184 | 18 | 0.097826 |
| E02004072 | 392 | 27 | 0.068878 |
| E02004073 | 321 | 26 | 0.080997 |
| E02004074 | 145 | 11 | 0.075862 |
| E02004075 | 233 | 16 | 0.06867 |
| E02004076 | 306 | 23 | 0.075163 |
| E02004077 | 505 | 10 | 0.019802 |
| E02004078 | 463 | 26 | 0.056156 |
| E02004080 | 652 | 34 | 0.052147 |
| E02004081 | 293 | 24 | 0.081911 |
| E02004082 | 329 | 13 | 0.039514 |
| E02004083 | 546 | 19 | 0.034799 |
| E02004084 | 396 | 23 | 0.058081 |
| E02004085 | 585 | 23 | 0.039316 |
| E02004086 | 316 | 14 | 0.044304 |
| E02004087 | 464 | 23 | 0.049569 |
| E02004088 | 395 | 7 | 0.017722 |
| E02004089 | 415 | 23 | 0.055422 |
| E02004090 | 294 | 18 | 0.061224 |
| E02004091 | 572 | 25 | 0.043706 |
| E02004092 | 464 | 17 | 0.036638 |
| E02004097 | 341 | 21 | 0.061584 |
| E02004098 | 398 | 28 | 0.070352 |
| E02004100 | 352 | 25 | 0.071023 |
| E02004102 | 351 | 45 | 0.128205 |
| E02004103 | 392 | 32 | 0.081633 |
| E02004104 | 354 | 28 | 0.079096 |
| E02004105 | 562 | 33 | 0.058719 |
| E02004106 | 345 | 20 | 0.057971 |
| E02004108 | 408 | 29 | 0.071078 |
| E02004109 | 354 | 7 | 0.019774 |
| E02004110 | 344 | 17 | 0.049419 |
| E02004111 | 302 | 14 | 0.046358 |
| E02004112 | 257 | 18 | 0.070039 |
| E02004113 | 575 | 31 | 0.053913 |
| E02004114 | 393 | 29 | 0.073791 |
| E02004115 | 549 | 26 | 0.047359 |
| E02004116 | 519 | 52 | 0.100193 |
| E02004117 | 462 | 23 | 0.049784 |
| E02004118 | 540 | 16 | 0.02963 |
| E02004119 | 764 | 30 | 0.039267 |
| E02004120 | 827 | 40 | 0.048368 |
| E02004121 | 610 | 27 | 0.044262 |
| E02004122 | 356 | 8 | 0.022472 |
| E02004123 | 251 | 10 | 0.039841 |
| E02004124 | 545 | 23 | 0.042202 |
| E02004125 | 569 | 35 | 0.061511 |
| E02004126 | 509 | 26 | 0.051081 |
| E02004128 | 774 | 30 | 0.03876 |
| E02005334 | 535 | 17 | 0.031776 |
| E02005335 | 447 | 13 | 0.029083 |
| E02005336 | 578 | 23 | 0.039792 |
| E02005337 | 676 | 44 | 0.065089 |
| E02005338 | 768 | 30 | 0.039063 |
| E02005339 | 558 | 17 | 0.030466 |
| E02005340 | 563 | 29 | 0.05151 |
| E02005341 | 551 | 15 | 0.027223 |
| E02005342 | 406 | 13 | 0.03202 |
| E02005343 | 450 | 10 | 0.022222 |
| E02005344 | 541 | 19 | 0.03512 |
| E02005345 | 297 | 18 | 0.060606 |
| E02005346 | 916 | 43 | 0.046943 |
| E02005347 | 1290 | 47 | 0.036434 |
| E02005348 | 279 | 21 | 0.075269 |
| E02005349 | 445 | 22 | 0.049438 |
| E02005350 | 380 | 23 | 0.060526 |
| E02005351 | 898 | 27 | 0.030067 |
| E02005352 | 301 | 12 | 0.039867 |
| E02005353 | 695 | 24 | 0.034532 |
| E02005354 | 490 | 25 | 0.05102 |
| E02005355 | 333 | 11 | 0.033033 |
| E02005356 | 344 | 24 | 0.069767 |
| E02005357 | 462 | 25 | 0.054113 |
| E02005358 | 686 | 33 | 0.048105 |
| E02005359 | 534 | 22 | 0.041199 |
| E02005360 | 251 | 12 | 0.047809 |
| E02005361 | 398 | 19 | 0.047739 |
| E02005362 | 528 | 26 | 0.049242 |
| E02005363 | 729 | 33 | 0.045267 |
| E02005364 | 349 | 15 | 0.04298 |
| E02005365 | 902 | 51 | 0.056541 |
| E02005366 | 498 | 23 | 0.046185 |
| E02005368 | 224 | 12 | 0.053571 |
| E02005369 | 646 | 28 | 0.043344 |
| E02005370 | 506 | 17 | 0.033597 |
| E02005371 | 517 | 22 | 0.042553 |
| E02005372 | 516 | 7 | 0.013566 |
| E02005373 | 464 | 21 | 0.045259 |
| E02005374 | 293 | 22 | 0.075085 |
| E02005375 | 458 | 32 | 0.069869 |
| E02005376 | 530 | 26 | 0.049057 |
| E02005377 | 451 | 21 | 0.046563 |
| E02005378 | 351 | 18 | 0.051282 |
| E02005379 | 403 | 11 | 0.027295 |
| E02005380 | 400 | 7 | 0.0175 |
| E02005381 | 447 | 10 | 0.022371 |
| E02005382 | 494 | 18 | 0.036437 |
| E02005383 | 482 | 20 | 0.041494 |
| E02005384 | 323 | 6 | 0.018576 |
| E02005385 | 337 | 10 | 0.029674 |
| E02005386 | 531 | 23 | 0.043315 |
| E02005387 | 536 | 11 | 0.020522 |
| E02005388 | 280 | 15 | 0.053571 |
| E02005389 | 252 | 14 | 0.055556 |
| E02005390 | 428 | 11 | 0.025701 |
| E02005391 | 423 | 15 | 0.035461 |
| E02005392 | 510 | 24 | 0.047059 |
| E02005393 | 339 | 28 | 0.082596 |
| E02005394 | 549 | 38 | 0.069217 |
| E02005395 | 436 | 23 | 0.052752 |
| E02005396 | 288 | 13 | 0.045139 |
| E02005397 | 520 | 23 | 0.044231 |
| E02005398 | 327 | 13 | 0.039755 |
| E02005399 | 337 | 10 | 0.029674 |
| E02005400 | 272 | 17 | 0.0625 |
| E02005401 | 309 | 7 | 0.022654 |
| E02005402 | 373 | 14 | 0.037534 |
| E02005403 | 362 | 18 | 0.049724 |
| E02005404 | 475 | 18 | 0.037895 |
| E02005405 | 448 | 18 | 0.040179 |
| E02005406 | 498 | 25 | 0.050201 |
| E02005407 | 663 | 33 | 0.049774 |
| E02005408 | 379 | 11 | 0.029024 |
| E02005409 | 831 | 33 | 0.039711 |
| E02005412 | 745 | 46 | 0.061745 |
| E02005414 | 683 | 44 | 0.064422 |
| E02005415 | 638 | 39 | 0.061129 |
| E02005416 | 563 | 17 | 0.030195 |
| E02005417 | 480 | 24 | 0.05 |
| E02005418 | 735 | 31 | 0.042177 |
| E02005419 | 663 | 33 | 0.049774 |
| E02005420 | 608 | 35 | 0.057566 |
| E02005422 | 289 | 12 | 0.041522 |
| E02005423 | 587 | 42 | 0.07155 |
| E02005424 | 486 | 21 | 0.04321 |
| E02005425 | 150 | 15 | 0.1 |
| E02005426 | 456 | 35 | 0.076754 |
| E02005427 | 259 | 21 | 0.081081 |
| E02005428 | 303 | 59 | 0.194719 |
| E02005429 | 328 | 46 | 0.140244 |
| E02005430 | 252 | 10 | 0.039683 |
| E02005431 | 286 | 32 | 0.111888 |
| E02005432 | 301 | 23 | 0.076412 |
| E02005433 | 440 | 88 | 0.2 |
| E02005434 | 324 | 36 | 0.111111 |
| E02005435 | 374 | 43 | 0.114973 |
| E02005436 | 360 | 23 | 0.063889 |
| E02005437 | 377 | 40 | 0.106101 |
| E02005438 | 356 | 58 | 0.162921 |
| E02005439 | 539 | 32 | 0.059369 |
| E02005440 | 182 | 20 | 0.10989 |
| E02005441 | 270 | 21 | 0.077778 |
| E02005442 | 376 | 33 | 0.087766 |
| E02005443 | 436 | 26 | 0.059633 |
| E02005444 | 899 | 37 | 0.041157 |
| E02005445 | 840 | 41 | 0.04881 |
| E02005446 | 842 | 21 | 0.024941 |
| E02005447 | 655 | 31 | 0.047328 |
| E02005448 | 419 | 27 | 0.064439 |
| E02005449 | 582 | 28 | 0.04811 |
| E02005450 | 462 | 33 | 0.071429 |
| E02005451 | 510 | 28 | 0.054902 |
| E02005452 | 644 | 34 | 0.052795 |
| E02005453 | 525 | 24 | 0.045714 |
| E02005455 | 622 | 31 | 0.049839 |
| E02005456 | 540 | 30 | 0.055556 |
| E02005457 | 395 | 22 | 0.055696 |
| E02005458 | 251 | 15 | 0.059761 |
| E02005459 | 388 | 18 | 0.046392 |
| E02005460 | 238 | 9 | 0.037815 |
| E02005461 | 213 | 21 | 0.098592 |
| E02005462 | 422 | 19 | 0.045024 |
| E02005463 | 436 | 16 | 0.036697 |
| E02005464 | 407 | 29 | 0.071253 |
| E02005465 | 365 | 26 | 0.071233 |
| E02005466 | 253 | 20 | 0.079051 |
| E02005467 | 368 | 39 | 0.105978 |
| E02005468 | 279 | 21 | 0.075269 |
| E02005469 | 308 | 19 | 0.061688 |
| E02005470 | 935 | 37 | 0.039572 |
| E02005471 | 569 | 40 | 0.070299 |
| E02005472 | 529 | 28 | 0.05293 |
| E02005473 | 577 | 30 | 0.051993 |
| E02005474 | 279 | 20 | 0.071685 |
| E02005475 | 304 | 17 | 0.055921 |
| E02005476 | 313 | 8 | 0.025559 |
| E02005477 | 421 | 24 | 0.057007 |
| E02005478 | 753 | 51 | 0.067729 |
| E02005479 | 728 | 44 | 0.06044 |
| E02005480 | 527 | 27 | 0.051233 |
| E02005481 | 912 | 59 | 0.064693 |
| E02005482 | 483 | 21 | 0.043478 |
| E02005483 | 295 | 23 | 0.077966 |
| E02005484 | 289 | 14 | 0.048443 |
| E02005485 | 333 | 26 | 0.078078 |
| E02005486 | 393 | 30 | 0.076336 |
| E02005487 | 328 | 22 | 0.067073 |
| E02005488 | 320 | 19 | 0.059375 |
| E02005489 | 268 | 11 | 0.041045 |
| E02005490 | 411 | 34 | 0.082725 |
| E02005491 | 380 | 37 | 0.097368 |
| E02005492 | 411 | 24 | 0.058394 |
| E02005493 | 533 | 25 | 0.046904 |
| E02005494 | 283 | 24 | 0.084806 |
| E02005495 | 549 | 39 | 0.071038 |
| E02005496 | 280 | 17 | 0.060714 |
| E02005497 | 430 | 40 | 0.093023 |
| E02005498 | 351 | 15 | 0.042735 |
| E02005499 | 388 | 11 | 0.028351 |
| E02005500 | 402 | 22 | 0.054726 |
| E02005501 | 382 | 19 | 0.049738 |
| E02005502 | 498 | 23 | 0.046185 |
| E02005612 | 619 | 32 | 0.051696 |
| E02005613 | 800 | 32 | 0.04 |
| E02005614 | 885 | 33 | 0.037288 |
| E02005615 | 741 | 46 | 0.062078 |
| E02005616 | 463 | 21 | 0.045356 |
| E02005617 | 857 | 27 | 0.031505 |
| E02005619 | 208 | 7 | 0.033654 |
| E02005620 | 370 | 23 | 0.062162 |
| E02005621 | 427 | 22 | 0.051522 |
| E02005622 | 435 | 13 | 0.029885 |
| E02005623 | 592 | 27 | 0.045608 |
| E02005624 | 535 | 33 | 0.061682 |
| E02005625 | 431 | 43 | 0.099768 |
| E02005626 | 332 | 26 | 0.078313 |
| E02005627 | 325 | 18 | 0.055385 |
| E02005628 | 334 | 17 | 0.050898 |
| E02005629 | 217 | 10 | 0.046083 |
| E02005630 | 339 | 7 | 0.020649 |
| E02005631 | 413 | 23 | 0.05569 |
| E02005632 | 276 | 17 | 0.061594 |
| E02005633 | 520 | 42 | 0.080769 |
| E02005634 | 522 | 44 | 0.084291 |
| E02005635 | 455 | 26 | 0.057143 |
| E02005636 | 621 | 28 | 0.045089 |
| E02005637 | 582 | 30 | 0.051546 |
| E02005638 | 554 | 29 | 0.052347 |
| E02005639 | 182 | 12 | 0.065934 |
| E02005640 | 659 | 26 | 0.039454 |
| E02005641 | 384 | 25 | 0.065104 |
| E02005642 | 447 | 21 | 0.04698 |
| E02005643 | 484 | 21 | 0.043388 |
| E02005644 | 611 | 45 | 0.07365 |
| E02005645 | 888 | 54 | 0.060811 |
| E02005646 | 301 | 28 | 0.093023 |
| E02005647 | 418 | 19 | 0.045455 |
| E02005648 | 475 | 26 | 0.054737 |
| E02005649 | 812 | 50 | 0.061576 |
| E02005650 | 431 | 20 | 0.046404 |
| E02005651 | 449 | 19 | 0.042316 |
| E02005652 | 404 | 29 | 0.071782 |
| E02005653 | 692 | 39 | 0.056358 |
| E02005654 | 580 | 28 | 0.048276 |
| E02005655 | 416 | 28 | 0.067308 |
| E02005656 | 534 | 33 | 0.061798 |
| E02005657 | 377 | 22 | 0.058355 |
| E02005658 | 519 | 23 | 0.044316 |
| E02005659 | 445 | 29 | 0.065169 |
| E02005660 | 528 | 30 | 0.056818 |
| E02005661 | 538 | 36 | 0.066914 |
| E02005662 | 478 | 22 | 0.046025 |
| E02005663 | 379 | 19 | 0.050132 |
| E02005664 | 472 | 37 | 0.07839 |
| E02005665 | 637 | 35 | 0.054945 |
| E02005666 | 757 | 53 | 0.070013 |
| E02005667 | 520 | 36 | 0.069231 |
| E02005668 | 404 | 27 | 0.066832 |
| E02005669 | 498 | 32 | 0.064257 |
| E02005670 | 734 | 37 | 0.050409 |
| E02005671 | 616 | 27 | 0.043831 |
| E02005672 | 698 | 27 | 0.038682 |
| E02005673 | 637 | 42 | 0.065934 |
| E02005674 | 808 | 54 | 0.066832 |
| E02005675 | 401 | 23 | 0.057357 |
| E02005676 | 456 | 25 | 0.054825 |
| E02005677 | 692 | 23 | 0.033237 |
| E02005678 | 1065 | 52 | 0.048826 |
| E02005679 | 532 | 18 | 0.033835 |
| E02005680 | 585 | 19 | 0.032479 |
| E02005681 | 470 | 30 | 0.06383 |
| E02005682 | 344 | 20 | 0.05814 |
| E02005683 | 747 | 35 | 0.046854 |
| E02005684 | 362 | 19 | 0.052486 |
| E02005685 | 506 | 24 | 0.047431 |
| E02005686 | 416 | 16 | 0.038462 |
| E02005687 | 225 | 12 | 0.053333 |
| E02005688 | 464 | 24 | 0.051724 |
| E02005689 | 493 | 23 | 0.046653 |
| E02005690 | 189 | 16 | 0.084656 |
| E02005691 | 202 | 9 | 0.044554 |
| E02005692 | 260 | 22 | 0.084615 |
| E02005693 | 504 | 27 | 0.053571 |
| E02005694 | 416 | 27 | 0.064904 |
| E02005695 | 709 | 36 | 0.050776 |
| E02005696 | 385 | 27 | 0.07013 |
| E02005697 | 548 | 36 | 0.065693 |
| E02005698 | 366 | 32 | 0.087432 |
| E02005699 | 515 | 24 | 0.046602 |
| E02005700 | 492 | 16 | 0.03252 |
| E02005701 | 520 | 34 | 0.065385 |
| E02005819 | 389 | 18 | 0.046272 |
| E02005820 | 413 | 12 | 0.029056 |
| E02005821 | 468 | 20 | 0.042735 |
| E02005822 | 756 | 53 | 0.070106 |
| E02005823 | 723 | 36 | 0.049793 |
| E02005824 | 696 | 17 | 0.024425 |
| E02005825 | 635 | 28 | 0.044094 |
| E02005826 | 473 | 16 | 0.033827 |
| E02005827 | 680 | 21 | 0.030882 |
| E02005828 | 544 | 26 | 0.047794 |
| E02005829 | 513 | 18 | 0.035088 |
| E02005830 | 340 | 21 | 0.061765 |
| E02005831 | 756 | 39 | 0.051587 |
| E02005832 | 652 | 41 | 0.062883 |
| E02005833 | 513 | 23 | 0.044834 |
| E02005834 | 662 | 22 | 0.033233 |
| E02005835 | 835 | 42 | 0.050299 |
| E02005836 | 431 | 20 | 0.046404 |
| E02005837 | 680 | 16 | 0.023529 |
| E02005838 | 496 | 24 | 0.048387 |
| E02005839 | 349 | 26 | 0.074499 |
| E02005840 | 723 | 43 | 0.059474 |
| E02005842 | 519 | 40 | 0.077071 |
| E02005843 | 469 | 23 | 0.049041 |
| E02005844 | 519 | 25 | 0.04817 |
| E02005846 | 520 | 40 | 0.076923 |
| E02005847 | 528 | 30 | 0.056818 |
| E02005848 | 408 | 29 | 0.071078 |
| E02005849 | 415 | 23 | 0.055422 |
| E02005850 | 477 | 19 | 0.039832 |
| E02005851 | 379 | 9 | 0.023747 |
| E02005852 | 514 | 24 | 0.046693 |
| E02005853 | 484 | 36 | 0.07438 |
| E02005856 | 324 | 17 | 0.052469 |
| E02005857 | 370 | 23 | 0.062162 |
| E02005858 | 671 | 15 | 0.022355 |
| E02005859 | 557 | 25 | 0.044883 |
| E02005860 | 590 | 17 | 0.028814 |
| E02005861 | 278 | 13 | 0.046763 |
| E02005862 | 427 | 30 | 0.070258 |
| E02005863 | 371 | 15 | 0.040431 |
| E02005864 | 438 | 22 | 0.050228 |
| E02005865 | 434 | 5 | 0.011521 |
| E02005866 | 536 | 30 | 0.05597 |
| E02005868 | 579 | 26 | 0.044905 |
| E02005869 | 479 | 36 | 0.075157 |
| E02005870 | 482 | 29 | 0.060166 |
| E02005871 | 478 | 24 | 0.050209 |
| E02005872 | 307 | 15 | 0.04886 |
| E02005873 | 658 | 30 | 0.045593 |
| E02005874 | 447 | 15 | 0.033557 |
| E02005875 | 605 | 35 | 0.057851 |
| E02005876 | 637 | 27 | 0.042386 |
| E02005877 | 526 | 24 | 0.045627 |
| E02005878 | 766 | 41 | 0.053525 |
| E02005879 | 426 | 25 | 0.058685 |
| E02005880 | 445 | 30 | 0.067416 |
| E02005881 | 471 | 26 | 0.055202 |
| E02005882 | 516 | 32 | 0.062016 |
| E02005883 | 677 | 32 | 0.047267 |
| E02005884 | 389 | 25 | 0.064267 |
| E02005885 | 596 | 27 | 0.045302 |
| E02005886 | 866 | 24 | 0.027714 |
| E02005887 | 847 | 41 | 0.048406 |
| E02005888 | 641 | 31 | 0.048362 |
| E02005889 | 844 | 44 | 0.052133 |
| E02005890 | 430 | 16 | 0.037209 |
| E02005891 | 433 | 37 | 0.08545 |
| E02005892 | 902 | 38 | 0.042129 |
| E02005893 | 856 | 50 | 0.058411 |
| E02005894 | 820 | 25 | 0.030488 |
| E02005895 | 301 | 17 | 0.056478 |
| E02005896 | 279 | 19 | 0.0681 |
| E02005897 | 490 | 32 | 0.065306 |
| E02005898 | 881 | 38 | 0.043133 |
| E02005899 | 734 | 43 | 0.058583 |
| E02005900 | 300 | 15 | 0.05 |
| E02005901 | 460 | 30 | 0.065217 |
| E02005902 | 420 | 24 | 0.057143 |
| E02005903 | 510 | 36 | 0.070588 |
| E02005904 | 465 | 24 | 0.051613 |
| E02005905 | 503 | 25 | 0.049702 |
| E02005906 | 677 | 15 | 0.022157 |
| E02005907 | 406 | 18 | 0.044335 |
| E02005908 | 397 | 14 | 0.035264 |
| E02005909 | 396 | 15 | 0.037879 |
| E02005910 | 637 | 17 | 0.026688 |
| E02005911 | 448 | 22 | 0.049107 |
| E02005912 | 589 | 28 | 0.047538 |
| E02005913 | 446 | 23 | 0.05157 |
| E02005914 | 559 | 31 | 0.055456 |
| E02005915 | 194 | 11 | 0.056701 |
| E02005916 | 530 | 16 | 0.030189 |
| E02005917 | 292 | 6 | 0.020548 |
| E02005918 | 246 | 4 | 0.01626 |
| E02005919 | 342 | 9 | 0.026316 |
| E02005920 | 362 | 5 | 0.013812 |
| E02006804 | 450 | 29 | 0.064444 |
| E02006815 | 1190 | 59 | 0.04958 |
| E02006816 | 667 | 29 | 0.043478 |
| E02006817 | 676 | 31 | 0.045858 |
| E02006818 | 628 | 43 | 0.068471 |
| E02006819 | 716 | 45 | 0.062849 |
| E02006820 | 709 | 22 | 0.03103 |
| E02006827 | 533 | 31 | 0.058161 |
| E02006828 | 502 | 26 | 0.051793 |
| E02006834 | 554 | 46 | 0.083032 |
| E02006835 | 457 | 19 | 0.041575 |
| E02006850 | 825 | 32 | 0.038788 |
| E02006851 | 850 | 21 | 0.024706 |
| E02006862 | 408 | 16 | 0.039216 |
| E02006863 | 827 | 33 | 0.039903 |
| E02006864 | 584 | 32 | 0.054795 |
| E02006865 | 658 | 37 | 0.056231 |
| E02006866 | 472 | 20 | 0.042373 |
| E02006867 | 372 | 31 | 0.083333 |
| E02006872 | 611 | 18 | 0.02946 |
| E02006903 | 927 | 53 | 0.057174 |
| E02006904 | 842 | 34 | 0.04038 |
| E02006905 | 543 | 44 | 0.081031 |
| E02006906 | 883 | 38 | 0.043035 |
| E02006911 | 1512 | 49 | 0.032407 |
| E02006919 | 665 | 42 | 0.063158 |
| E02006920 | 573 | 23 | 0.04014 |

**Table S8.** MHVI score (calculated from cumulative weekly GOV.UK positive case numbers and daily EMAS suspected severe illness case numbers) for cumulative period two (C3) between May 18^th^, 2020, and October 2^nd^, 2021.

| **MSOA** | **GOV.UK** | **EMAS** | **MHVI** |
| --- | --- | --- | --- |
| E02002796 | 604 | 17 | 0.028146 |
| E02002797 | 715 | 27 | 0.037762 |
| E02002798 | 1248 | 33 | 0.026442 |
| E02002799 | 888 | 29 | 0.032658 |
| E02002800 | 792 | 18 | 0.022727 |
| E02002801 | 960 | 45 | 0.046875 |
| E02002802 | 1085 | 31 | 0.028571 |
| E02002803 | 890 | 35 | 0.039326 |
| E02002804 | 1093 | 37 | 0.033852 |
| E02002805 | 575 | 15 | 0.026087 |
| E02002806 | 711 | 31 | 0.043601 |
| E02002807 | 1092 | 36 | 0.032967 |
| E02002808 | 1489 | 44 | 0.02955 |
| E02002809 | 875 | 48 | 0.054857 |
| E02002810 | 1105 | 47 | 0.042534 |
| E02002811 | 1409 | 64 | 0.045422 |
| E02002812 | 1144 | 33 | 0.028846 |
| E02002813 | 1597 | 78 | 0.048842 |
| E02002814 | 923 | 27 | 0.029252 |
| E02002815 | 1157 | 39 | 0.033708 |
| E02002816 | 1352 | 37 | 0.027367 |
| E02002817 | 826 | 40 | 0.048426 |
| E02002818 | 1070 | 31 | 0.028972 |
| E02002819 | 1045 | 42 | 0.040191 |
| E02002820 | 1275 | 37 | 0.02902 |
| E02002821 | 1537 | 53 | 0.034483 |
| E02002822 | 958 | 40 | 0.041754 |
| E02002823 | 982 | 42 | 0.04277 |
| E02002824 | 1450 | 53 | 0.036552 |
| E02002825 | 1149 | 35 | 0.030461 |
| E02002826 | 909 | 19 | 0.020902 |
| E02002827 | 1061 | 33 | 0.031103 |
| E02002828 | 1234 | 44 | 0.035656 |
| E02002829 | 1979 | 54 | 0.027287 |
| E02002830 | 1722 | 65 | 0.037747 |
| E02002831 | 1203 | 50 | 0.041563 |
| E02002832 | 1639 | 63 | 0.038438 |
| E02002833 | 1363 | 40 | 0.029347 |
| E02002834 | 1945 | 40 | 0.020566 |
| E02002835 | 2671 | 87 | 0.032572 |
| E02002836 | 1617 | 52 | 0.032158 |
| E02002837 | 1557 | 30 | 0.019268 |
| E02002838 | 1280 | 46 | 0.035938 |
| E02002839 | 1473 | 53 | 0.035981 |
| E02002842 | 1187 | 23 | 0.019377 |
| E02002843 | 2391 | 70 | 0.029276 |
| E02002844 | 1963 | 60 | 0.030565 |
| E02002845 | 1815 | 75 | 0.041322 |
| E02002846 | 1010 | 15 | 0.014851 |
| E02002847 | 1892 | 54 | 0.028541 |
| E02002848 | 1952 | 54 | 0.027664 |
| E02002849 | 2031 | 36 | 0.017725 |
| E02002851 | 1530 | 46 | 0.030065 |
| E02002852 | 1211 | 40 | 0.033031 |
| E02002853 | 2361 | 78 | 0.033037 |
| E02002854 | 1328 | 36 | 0.027108 |
| E02002855 | 1094 | 17 | 0.015539 |
| E02002856 | 1656 | 35 | 0.021135 |
| E02002857 | 1060 | 32 | 0.030189 |
| E02002858 | 950 | 39 | 0.041053 |
| E02002860 | 1235 | 36 | 0.02915 |
| E02002861 | 1148 | 44 | 0.038328 |
| E02002862 | 1140 | 44 | 0.038596 |
| E02002863 | 815 | 11 | 0.013497 |
| E02002864 | 667 | 25 | 0.037481 |
| E02002865 | 533 | 16 | 0.030019 |
| E02002866 | 561 | 17 | 0.030303 |
| E02002867 | 710 | 9 | 0.012676 |
| E02002868 | 854 | 25 | 0.029274 |
| E02002869 | 1145 | 43 | 0.037555 |
| E02002871 | 1073 | 39 | 0.036347 |
| E02002872 | 1236 | 39 | 0.031553 |
| E02002873 | 1467 | 43 | 0.029312 |
| E02002874 | 1498 | 46 | 0.030708 |
| E02002875 | 1207 | 37 | 0.030655 |
| E02002876 | 1184 | 38 | 0.032095 |
| E02002877 | 944 | 38 | 0.040254 |
| E02002878 | 1372 | 47 | 0.034257 |
| E02002879 | 1389 | 52 | 0.037437 |
| E02002880 | 1305 | 39 | 0.029885 |
| E02002881 | 1130 | 23 | 0.020354 |
| E02002882 | 1198 | 27 | 0.022538 |
| E02002883 | 999 | 41 | 0.041041 |
| E02002884 | 1163 | 71 | 0.061049 |
| E02002885 | 1399 | 51 | 0.036455 |
| E02002886 | 1563 | 48 | 0.03071 |
| E02002887 | 950 | 57 | 0.06 |
| E02002888 | 1019 | 33 | 0.032385 |
| E02002889 | 2530 | 27 | 0.010672 |
| E02002890 | 1897 | 29 | 0.015287 |
| E02002891 | 1169 | 38 | 0.032506 |
| E02002892 | 904 | 34 | 0.037611 |
| E02002893 | 2278 | 47 | 0.020632 |
| E02002894 | 965 | 31 | 0.032124 |
| E02002895 | 1948 | 28 | 0.014374 |
| E02002896 | 1397 | 54 | 0.038654 |
| E02002897 | 891 | 30 | 0.03367 |
| E02002898 | 2221 | 91 | 0.040973 |
| E02002899 | 1825 | 26 | 0.014247 |
| E02002901 | 846 | 24 | 0.028369 |
| E02002902 | 1387 | 43 | 0.031002 |
| E02002903 | 793 | 26 | 0.032787 |
| E02002904 | 953 | 47 | 0.049318 |
| E02004029 | 819 | 33 | 0.040293 |
| E02004030 | 488 | 16 | 0.032787 |
| E02004031 | 953 | 43 | 0.045121 |
| E02004032 | 1070 | 30 | 0.028037 |
| E02004033 | 1108 | 52 | 0.046931 |
| E02004034 | 735 | 37 | 0.05034 |
| E02004035 | 629 | 13 | 0.020668 |
| E02004036 | 1346 | 52 | 0.038633 |
| E02004037 | 987 | 19 | 0.01925 |
| E02004038 | 814 | 36 | 0.044226 |
| E02004039 | 877 | 25 | 0.028506 |
| E02004040 | 1198 | 36 | 0.03005 |
| E02004041 | 975 | 41 | 0.042051 |
| E02004043 | 876 | 26 | 0.02968 |
| E02004044 | 1029 | 25 | 0.024295 |
| E02004045 | 1100 | 25 | 0.022727 |
| E02004046 | 681 | 22 | 0.032305 |
| E02004047 | 765 | 27 | 0.035294 |
| E02004048 | 769 | 28 | 0.036411 |
| E02004049 | 930 | 32 | 0.034409 |
| E02004050 | 1188 | 33 | 0.027778 |
| E02004051 | 1519 | 51 | 0.033575 |
| E02004052 | 1121 | 50 | 0.044603 |
| E02004053 | 870 | 28 | 0.032184 |
| E02004054 | 1148 | 58 | 0.050523 |
| E02004055 | 678 | 28 | 0.041298 |
| E02004056 | 897 | 34 | 0.037904 |
| E02004057 | 851 | 27 | 0.031727 |
| E02004058 | 803 | 47 | 0.058531 |
| E02004059 | 761 | 32 | 0.04205 |
| E02004060 | 822 | 37 | 0.045012 |
| E02004061 | 749 | 32 | 0.042724 |
| E02004062 | 635 | 19 | 0.029921 |
| E02004063 | 767 | 34 | 0.044329 |
| E02004064 | 1028 | 40 | 0.038911 |
| E02004065 | 733 | 22 | 0.030014 |
| E02004066 | 1280 | 40 | 0.03125 |
| E02004067 | 604 | 32 | 0.05298 |
| E02004068 | 549 | 22 | 0.040073 |
| E02004069 | 459 | 17 | 0.037037 |
| E02004070 | 452 | 16 | 0.035398 |
| E02004071 | 423 | 20 | 0.047281 |
| E02004072 | 921 | 31 | 0.033659 |
| E02004073 | 688 | 27 | 0.039244 |
| E02004074 | 382 | 16 | 0.041885 |
| E02004075 | 506 | 18 | 0.035573 |
| E02004076 | 755 | 26 | 0.034437 |
| E02004077 | 956 | 14 | 0.014644 |
| E02004078 | 908 | 32 | 0.035242 |
| E02004080 | 1471 | 44 | 0.029912 |
| E02004081 | 560 | 27 | 0.048214 |
| E02004082 | 762 | 18 | 0.023622 |
| E02004083 | 1108 | 21 | 0.018953 |
| E02004084 | 876 | 24 | 0.027397 |
| E02004085 | 1075 | 29 | 0.026977 |
| E02004086 | 753 | 19 | 0.025232 |
| E02004087 | 966 | 25 | 0.02588 |
| E02004088 | 879 | 7 | 0.007964 |
| E02004089 | 880 | 30 | 0.034091 |
| E02004090 | 588 | 21 | 0.035714 |
| E02004091 | 1081 | 28 | 0.025902 |
| E02004092 | 1019 | 19 | 0.018646 |
| E02004097 | 656 | 26 | 0.039634 |
| E02004098 | 761 | 34 | 0.044678 |
| E02004100 | 745 | 27 | 0.036242 |
| E02004102 | 857 | 48 | 0.056009 |
| E02004103 | 980 | 37 | 0.037755 |
| E02004104 | 857 | 31 | 0.036173 |
| E02004105 | 1193 | 37 | 0.031014 |
| E02004106 | 670 | 22 | 0.032836 |
| E02004108 | 838 | 30 | 0.0358 |
| E02004109 | 683 | 12 | 0.01757 |
| E02004110 | 818 | 19 | 0.023227 |
| E02004111 | 546 | 14 | 0.025641 |
| E02004112 | 566 | 22 | 0.038869 |
| E02004113 | 1025 | 39 | 0.038049 |
| E02004114 | 730 | 33 | 0.045205 |
| E02004115 | 960 | 28 | 0.029167 |
| E02004116 | 988 | 62 | 0.062753 |
| E02004117 | 837 | 29 | 0.034648 |
| E02004118 | 997 | 18 | 0.018054 |
| E02004119 | 1526 | 31 | 0.020315 |
| E02004120 | 1517 | 43 | 0.028345 |
| E02004121 | 1147 | 32 | 0.027899 |
| E02004122 | 687 | 8 | 0.011645 |
| E02004123 | 618 | 13 | 0.021036 |
| E02004124 | 837 | 24 | 0.028674 |
| E02004125 | 1027 | 43 | 0.04187 |
| E02004126 | 846 | 30 | 0.035461 |
| E02004128 | 1272 | 34 | 0.02673 |
| E02005334 | 1007 | 17 | 0.016882 |
| E02005335 | 1058 | 16 | 0.015123 |
| E02005336 | 1176 | 27 | 0.022959 |
| E02005337 | 1462 | 46 | 0.031464 |
| E02005338 | 1569 | 36 | 0.022945 |
| E02005339 | 1158 | 19 | 0.016408 |
| E02005340 | 1068 | 33 | 0.030899 |
| E02005341 | 993 | 18 | 0.018127 |
| E02005342 | 857 | 13 | 0.015169 |
| E02005343 | 924 | 12 | 0.012987 |
| E02005344 | 1084 | 22 | 0.020295 |
| E02005345 | 652 | 18 | 0.027607 |
| E02005346 | 1777 | 47 | 0.026449 |
| E02005347 | 1896 | 54 | 0.028481 |
| E02005348 | 553 | 24 | 0.0434 |
| E02005349 | 936 | 27 | 0.028846 |
| E02005350 | 764 | 30 | 0.039267 |
| E02005351 | 1458 | 32 | 0.021948 |
| E02005352 | 675 | 13 | 0.019259 |
| E02005353 | 1311 | 28 | 0.021358 |
| E02005354 | 984 | 32 | 0.03252 |
| E02005355 | 827 | 12 | 0.01451 |
| E02005356 | 773 | 30 | 0.03881 |
| E02005357 | 1015 | 27 | 0.026601 |
| E02005358 | 1481 | 40 | 0.027009 |
| E02005359 | 1004 | 24 | 0.023904 |
| E02005360 | 594 | 12 | 0.020202 |
| E02005361 | 823 | 22 | 0.026731 |
| E02005362 | 978 | 32 | 0.03272 |
| E02005363 | 1300 | 37 | 0.028462 |
| E02005364 | 672 | 17 | 0.025298 |
| E02005365 | 1485 | 57 | 0.038384 |
| E02005366 | 969 | 27 | 0.027864 |
| E02005368 | 559 | 13 | 0.023256 |
| E02005369 | 1471 | 31 | 0.021074 |
| E02005370 | 1133 | 19 | 0.01677 |
| E02005371 | 1137 | 24 | 0.021108 |
| E02005372 | 1101 | 10 | 0.009083 |
| E02005373 | 1058 | 24 | 0.022684 |
| E02005374 | 664 | 26 | 0.039157 |
| E02005375 | 1000 | 38 | 0.038 |
| E02005376 | 1260 | 30 | 0.02381 |
| E02005377 | 1095 | 23 | 0.021005 |
| E02005378 | 636 | 22 | 0.034591 |
| E02005379 | 779 | 15 | 0.019255 |
| E02005380 | 936 | 9 | 0.009615 |
| E02005381 | 1093 | 11 | 0.010064 |
| E02005382 | 1039 | 22 | 0.021174 |
| E02005383 | 1006 | 22 | 0.021869 |
| E02005384 | 725 | 9 | 0.012414 |
| E02005385 | 663 | 13 | 0.019608 |
| E02005386 | 1106 | 29 | 0.026221 |
| E02005387 | 1071 | 15 | 0.014006 |
| E02005388 | 615 | 17 | 0.027642 |
| E02005389 | 590 | 15 | 0.025424 |
| E02005390 | 1137 | 12 | 0.010554 |
| E02005391 | 998 | 19 | 0.019038 |
| E02005392 | 1033 | 26 | 0.025169 |
| E02005393 | 669 | 29 | 0.043348 |
| E02005394 | 1049 | 44 | 0.041945 |
| E02005395 | 957 | 23 | 0.024033 |
| E02005396 | 589 | 13 | 0.022071 |
| E02005397 | 1274 | 25 | 0.019623 |
| E02005398 | 789 | 17 | 0.021546 |
| E02005399 | 873 | 12 | 0.013746 |
| E02005400 | 600 | 24 | 0.04 |
| E02005401 | 708 | 10 | 0.014124 |
| E02005402 | 842 | 16 | 0.019002 |
| E02005403 | 746 | 19 | 0.025469 |
| E02005404 | 1049 | 20 | 0.019066 |
| E02005405 | 847 | 20 | 0.023613 |
| E02005406 | 1074 | 28 | 0.026071 |
| E02005407 | 1439 | 38 | 0.026407 |
| E02005408 | 900 | 13 | 0.014444 |
| E02005409 | 1694 | 38 | 0.022432 |
| E02005412 | 1276 | 50 | 0.039185 |
| E02005414 | 1144 | 49 | 0.042832 |
| E02005415 | 1124 | 45 | 0.040036 |
| E02005416 | 1058 | 21 | 0.019849 |
| E02005417 | 743 | 25 | 0.033647 |
| E02005418 | 1194 | 33 | 0.027638 |
| E02005419 | 1109 | 33 | 0.029757 |
| E02005420 | 1017 | 41 | 0.040315 |
| E02005422 | 647 | 18 | 0.027821 |
| E02005423 | 1080 | 45 | 0.041667 |
| E02005424 | 1022 | 23 | 0.022505 |
| E02005425 | 421 | 18 | 0.042755 |
| E02005426 | 1087 | 40 | 0.036799 |
| E02005427 | 633 | 21 | 0.033175 |
| E02005428 | 679 | 66 | 0.097202 |
| E02005429 | 474 | 48 | 0.101266 |
| E02005430 | 525 | 13 | 0.024762 |
| E02005431 | 615 | 35 | 0.056911 |
| E02005432 | 564 | 27 | 0.047872 |
| E02005433 | 825 | 93 | 0.112727 |
| E02005434 | 626 | 39 | 0.0623 |
| E02005435 | 895 | 46 | 0.051397 |
| E02005436 | 781 | 26 | 0.033291 |
| E02005437 | 836 | 44 | 0.052632 |
| E02005438 | 874 | 58 | 0.066362 |
| E02005439 | 1247 | 37 | 0.029671 |
| E02005440 | 435 | 22 | 0.050575 |
| E02005441 | 590 | 22 | 0.037288 |
| E02005442 | 744 | 34 | 0.045699 |
| E02005443 | 882 | 29 | 0.03288 |
| E02005444 | 1636 | 40 | 0.02445 |
| E02005445 | 1566 | 45 | 0.028736 |
| E02005446 | 1510 | 26 | 0.017219 |
| E02005447 | 1157 | 35 | 0.030251 |
| E02005448 | 879 | 29 | 0.032992 |
| E02005449 | 1106 | 31 | 0.028029 |
| E02005450 | 866 | 35 | 0.040416 |
| E02005451 | 969 | 32 | 0.033024 |
| E02005452 | 1270 | 34 | 0.026772 |
| E02005453 | 952 | 24 | 0.02521 |
| E02005455 | 1447 | 37 | 0.02557 |
| E02005456 | 1185 | 35 | 0.029536 |
| E02005457 | 804 | 28 | 0.034826 |
| E02005458 | 503 | 18 | 0.035785 |
| E02005459 | 666 | 21 | 0.031532 |
| E02005460 | 574 | 10 | 0.017422 |
| E02005461 | 393 | 22 | 0.05598 |
| E02005462 | 916 | 23 | 0.025109 |
| E02005463 | 883 | 19 | 0.021518 |
| E02005464 | 657 | 30 | 0.045662 |
| E02005465 | 766 | 28 | 0.036554 |
| E02005466 | 527 | 21 | 0.039848 |
| E02005467 | 671 | 46 | 0.068554 |
| E02005468 | 615 | 23 | 0.037398 |
| E02005469 | 603 | 22 | 0.036484 |
| E02005470 | 1474 | 40 | 0.027137 |
| E02005471 | 941 | 42 | 0.044633 |
| E02005472 | 925 | 32 | 0.034595 |
| E02005473 | 1010 | 31 | 0.030693 |
| E02005474 | 537 | 21 | 0.039106 |
| E02005475 | 621 | 24 | 0.038647 |
| E02005476 | 613 | 10 | 0.016313 |
| E02005477 | 739 | 25 | 0.033829 |
| E02005478 | 1178 | 53 | 0.044992 |
| E02005479 | 1134 | 45 | 0.039683 |
| E02005480 | 888 | 29 | 0.032658 |
| E02005481 | 1485 | 62 | 0.041751 |
| E02005482 | 837 | 23 | 0.027479 |
| E02005483 | 631 | 25 | 0.03962 |
| E02005484 | 524 | 15 | 0.028626 |
| E02005485 | 787 | 27 | 0.034307 |
| E02005486 | 832 | 36 | 0.043269 |
| E02005487 | 723 | 25 | 0.034578 |
| E02005488 | 646 | 20 | 0.03096 |
| E02005489 | 597 | 14 | 0.023451 |
| E02005490 | 874 | 38 | 0.043478 |
| E02005491 | 1005 | 42 | 0.041791 |
| E02005492 | 829 | 27 | 0.032569 |
| E02005493 | 1062 | 30 | 0.028249 |
| E02005494 | 653 | 28 | 0.042879 |
| E02005495 | 1079 | 46 | 0.042632 |
| E02005496 | 530 | 17 | 0.032075 |
| E02005497 | 786 | 44 | 0.05598 |
| E02005498 | 593 | 17 | 0.028668 |
| E02005499 | 899 | 13 | 0.014461 |
| E02005500 | 724 | 26 | 0.035912 |
| E02005501 | 756 | 22 | 0.029101 |
| E02005502 | 943 | 26 | 0.027572 |
| E02005612 | 1094 | 35 | 0.031993 |
| E02005613 | 1364 | 39 | 0.028592 |
| E02005614 | 1664 | 43 | 0.025841 |
| E02005615 | 1336 | 49 | 0.036677 |
| E02005616 | 878 | 28 | 0.031891 |
| E02005617 | 1535 | 27 | 0.01759 |
| E02005619 | 486 | 10 | 0.020576 |
| E02005620 | 763 | 25 | 0.032765 |
| E02005621 | 810 | 23 | 0.028395 |
| E02005622 | 950 | 16 | 0.016842 |
| E02005623 | 1147 | 34 | 0.029643 |
| E02005624 | 1226 | 36 | 0.029364 |
| E02005625 | 943 | 45 | 0.04772 |
| E02005626 | 670 | 31 | 0.046269 |
| E02005627 | 715 | 21 | 0.029371 |
| E02005628 | 683 | 20 | 0.029283 |
| E02005629 | 458 | 11 | 0.024017 |
| E02005630 | 785 | 9 | 0.011465 |
| E02005631 | 917 | 24 | 0.026172 |
| E02005632 | 584 | 20 | 0.034247 |
| E02005633 | 961 | 52 | 0.05411 |
| E02005634 | 956 | 45 | 0.047071 |
| E02005635 | 907 | 31 | 0.034179 |
| E02005636 | 1267 | 33 | 0.026046 |
| E02005637 | 1022 | 36 | 0.035225 |
| E02005638 | 1068 | 32 | 0.029963 |
| E02005639 | 429 | 14 | 0.032634 |
| E02005640 | 1479 | 37 | 0.025017 |
| E02005641 | 998 | 27 | 0.027054 |
| E02005642 | 952 | 27 | 0.028361 |
| E02005643 | 1082 | 26 | 0.02403 |
| E02005644 | 1260 | 48 | 0.038095 |
| E02005645 | 1667 | 65 | 0.038992 |
| E02005646 | 733 | 30 | 0.040928 |
| E02005647 | 880 | 20 | 0.022727 |
| E02005648 | 1089 | 32 | 0.029385 |
| E02005649 | 1766 | 58 | 0.032843 |
| E02005650 | 757 | 23 | 0.030383 |
| E02005651 | 688 | 20 | 0.02907 |
| E02005652 | 687 | 36 | 0.052402 |
| E02005653 | 1076 | 46 | 0.042751 |
| E02005654 | 930 | 35 | 0.037634 |
| E02005655 | 660 | 33 | 0.05 |
| E02005656 | 909 | 34 | 0.037404 |
| E02005657 | 706 | 24 | 0.033994 |
| E02005658 | 805 | 24 | 0.029814 |
| E02005659 | 701 | 32 | 0.045649 |
| E02005660 | 863 | 34 | 0.039397 |
| E02005661 | 916 | 40 | 0.043668 |
| E02005662 | 830 | 24 | 0.028916 |
| E02005663 | 620 | 21 | 0.033871 |
| E02005664 | 774 | 42 | 0.054264 |
| E02005665 | 953 | 39 | 0.040923 |
| E02005666 | 1193 | 56 | 0.04694 |
| E02005667 | 784 | 38 | 0.048469 |
| E02005668 | 684 | 32 | 0.046784 |
| E02005669 | 747 | 33 | 0.044177 |
| E02005670 | 1147 | 39 | 0.034002 |
| E02005671 | 993 | 30 | 0.030211 |
| E02005672 | 1098 | 29 | 0.026412 |
| E02005673 | 914 | 47 | 0.051422 |
| E02005674 | 1226 | 59 | 0.048124 |
| E02005675 | 734 | 25 | 0.03406 |
| E02005676 | 836 | 25 | 0.029904 |
| E02005677 | 1161 | 26 | 0.022394 |
| E02005678 | 1833 | 59 | 0.032188 |
| E02005679 | 1007 | 19 | 0.018868 |
| E02005680 | 1250 | 23 | 0.0184 |
| E02005681 | 839 | 31 | 0.036949 |
| E02005682 | 663 | 23 | 0.034691 |
| E02005683 | 1499 | 41 | 0.027352 |
| E02005684 | 794 | 23 | 0.028967 |
| E02005685 | 1057 | 27 | 0.025544 |
| E02005686 | 714 | 18 | 0.02521 |
| E02005687 | 547 | 14 | 0.025594 |
| E02005688 | 981 | 26 | 0.026504 |
| E02005689 | 947 | 24 | 0.025343 |
| E02005690 | 364 | 18 | 0.049451 |
| E02005691 | 408 | 10 | 0.02451 |
| E02005692 | 576 | 26 | 0.045139 |
| E02005693 | 911 | 36 | 0.039517 |
| E02005694 | 804 | 29 | 0.03607 |
| E02005695 | 1170 | 41 | 0.035043 |
| E02005696 | 733 | 29 | 0.039563 |
| E02005697 | 915 | 43 | 0.046995 |
| E02005698 | 655 | 35 | 0.053435 |
| E02005699 | 954 | 28 | 0.02935 |
| E02005700 | 904 | 18 | 0.019912 |
| E02005701 | 1050 | 37 | 0.035238 |
| E02005819 | 733 | 20 | 0.027285 |
| E02005820 | 737 | 13 | 0.017639 |
| E02005821 | 897 | 25 | 0.027871 |
| E02005822 | 1439 | 60 | 0.041696 |
| E02005823 | 1246 | 43 | 0.03451 |
| E02005824 | 1364 | 22 | 0.016129 |
| E02005825 | 1086 | 31 | 0.028545 |
| E02005826 | 864 | 17 | 0.019676 |
| E02005827 | 1213 | 26 | 0.021434 |
| E02005828 | 938 | 30 | 0.031983 |
| E02005829 | 897 | 19 | 0.021182 |
| E02005830 | 684 | 21 | 0.030702 |
| E02005831 | 1401 | 45 | 0.03212 |
| E02005832 | 1288 | 42 | 0.032609 |
| E02005833 | 925 | 28 | 0.03027 |
| E02005834 | 1215 | 26 | 0.021399 |
| E02005835 | 1530 | 43 | 0.028105 |
| E02005836 | 979 | 23 | 0.023493 |
| E02005837 | 1131 | 18 | 0.015915 |
| E02005838 | 1035 | 27 | 0.026087 |
| E02005839 | 731 | 29 | 0.039672 |
| E02005840 | 1391 | 44 | 0.031632 |
| E02005842 | 1016 | 47 | 0.04626 |
| E02005843 | 916 | 28 | 0.030568 |
| E02005844 | 1118 | 29 | 0.025939 |
| E02005846 | 925 | 49 | 0.052973 |
| E02005847 | 962 | 33 | 0.034304 |
| E02005848 | 860 | 31 | 0.036047 |
| E02005849 | 948 | 28 | 0.029536 |
| E02005850 | 859 | 23 | 0.026775 |
| E02005851 | 794 | 15 | 0.018892 |
| E02005852 | 1017 | 26 | 0.025565 |
| E02005853 | 1036 | 39 | 0.037645 |
| E02005856 | 679 | 18 | 0.02651 |
| E02005857 | 725 | 26 | 0.035862 |
| E02005858 | 1147 | 19 | 0.016565 |
| E02005859 | 1102 | 27 | 0.024501 |
| E02005860 | 1109 | 22 | 0.019838 |
| E02005861 | 591 | 14 | 0.023689 |
| E02005862 | 902 | 35 | 0.038803 |
| E02005863 | 782 | 18 | 0.023018 |
| E02005864 | 976 | 27 | 0.027664 |
| E02005865 | 895 | 9 | 0.010056 |
| E02005866 | 1115 | 31 | 0.027803 |
| E02005868 | 1054 | 29 | 0.027514 |
| E02005869 | 864 | 40 | 0.046296 |
| E02005870 | 1000 | 32 | 0.032 |
| E02005871 | 905 | 27 | 0.029834 |
| E02005872 | 698 | 15 | 0.02149 |
| E02005873 | 1241 | 32 | 0.025786 |
| E02005874 | 1055 | 18 | 0.017062 |
| E02005875 | 1115 | 40 | 0.035874 |
| E02005876 | 1252 | 31 | 0.02476 |
| E02005877 | 1031 | 30 | 0.029098 |
| E02005878 | 1385 | 47 | 0.033935 |
| E02005879 | 886 | 26 | 0.029345 |
| E02005880 | 919 | 38 | 0.041349 |
| E02005881 | 946 | 29 | 0.030655 |
| E02005882 | 985 | 39 | 0.039594 |
| E02005883 | 1248 | 38 | 0.030449 |
| E02005884 | 813 | 27 | 0.03321 |
| E02005885 | 1078 | 39 | 0.036178 |
| E02005886 | 1650 | 28 | 0.01697 |
| E02005887 | 1635 | 48 | 0.029358 |
| E02005888 | 1195 | 39 | 0.032636 |
| E02005889 | 1483 | 48 | 0.032367 |
| E02005890 | 815 | 17 | 0.020859 |
| E02005891 | 823 | 38 | 0.046173 |
| E02005892 | 1771 | 44 | 0.024845 |
| E02005893 | 1620 | 56 | 0.034568 |
| E02005894 | 1584 | 29 | 0.018308 |
| E02005895 | 759 | 21 | 0.027668 |
| E02005896 | 614 | 22 | 0.035831 |
| E02005897 | 951 | 34 | 0.035752 |
| E02005898 | 1593 | 44 | 0.027621 |
| E02005899 | 1390 | 50 | 0.035971 |
| E02005900 | 759 | 18 | 0.023715 |
| E02005901 | 1063 | 40 | 0.037629 |
| E02005902 | 880 | 29 | 0.032955 |
| E02005903 | 1064 | 46 | 0.043233 |
| E02005904 | 947 | 29 | 0.030623 |
| E02005905 | 875 | 27 | 0.030857 |
| E02005906 | 997 | 15 | 0.015045 |
| E02005907 | 975 | 18 | 0.018462 |
| E02005908 | 1095 | 17 | 0.015525 |
| E02005909 | 1092 | 15 | 0.013736 |
| E02005910 | 1285 | 22 | 0.017121 |
| E02005911 | 1103 | 25 | 0.022665 |
| E02005912 | 1175 | 31 | 0.026383 |
| E02005913 | 1088 | 24 | 0.022059 |
| E02005914 | 1121 | 32 | 0.028546 |
| E02005915 | 512 | 13 | 0.025391 |
| E02005916 | 1090 | 18 | 0.016514 |
| E02005917 | 580 | 14 | 0.024138 |
| E02005918 | 484 | 6 | 0.012397 |
| E02005919 | 740 | 9 | 0.012162 |
| E02005920 | 780 | 9 | 0.011538 |
| E02006804 | 969 | 37 | 0.038184 |
| E02006815 | 1905 | 69 | 0.03622 |
| E02006816 | 1150 | 33 | 0.028696 |
| E02006817 | 1118 | 35 | 0.031306 |
| E02006818 | 1074 | 47 | 0.043762 |
| E02006819 | 1366 | 51 | 0.037335 |
| E02006820 | 1432 | 27 | 0.018855 |
| E02006827 | 1017 | 35 | 0.034415 |
| E02006828 | 962 | 30 | 0.031185 |
| E02006834 | 1058 | 49 | 0.046314 |
| E02006835 | 847 | 21 | 0.024793 |
| E02006850 | 1395 | 35 | 0.02509 |
| E02006851 | 1384 | 25 | 0.018064 |
| E02006862 | 797 | 22 | 0.027604 |
| E02006863 | 1614 | 36 | 0.022305 |
| E02006864 | 933 | 38 | 0.040729 |
| E02006865 | 1113 | 38 | 0.034142 |
| E02006866 | 985 | 24 | 0.024365 |
| E02006867 | 838 | 38 | 0.045346 |
| E02006872 | 1285 | 27 | 0.021012 |
| E02006903 | 1676 | 58 | 0.034606 |
| E02006904 | 1426 | 36 | 0.025245 |
| E02006905 | 946 | 46 | 0.048626 |
| E02006906 | 1522 | 45 | 0.029566 |
| E02006911 | 2403 | 53 | 0.022056 |
| E02006919 | 1224 | 47 | 0.038399 |
| E02006920 | 1018 | 25 | 0.024558 |
