# Supplementary material for: An early warning precision public health approach for assessing COVID-19 vulnerability in the UK: the Moore-Hill Vulnerability Index (MHVI)
Source: BMC Public Health. 2023 Nov 2;23:2147. doi: 10.1186/s12889-023-17092-7 (PMC10623819; doi:10.1186/s12889-023-17092-7)
Supplement: Supplementary file 3 — Supplementary Material 3 [file 12889_2023_17092_MOESM3_ESM.docx]

S-1

**Table S1.** MSOAs and cases excluded from full dataset (May 18^th^, 2020, to April 2^nd^, 2022), including records from 64 MSOAs reported by the East Midlands Ambulance NHS Trust data (EMAS), and records from 4 MSOAs reported by GOV.UK.

| **EMAS** | | **GOV.UK** | |
| --- | --- | --- | --- |
| MSOA | Cases | MSOA | Cases |
| E02001215 | 1 | E02004093 | 2126 |
| E02002726 | 31 | E02004094 | 2512 |
| E02002727 | 36 | E02004095 | 3233 |
| E02002728 | 24 | E02004096 | 3283 |
| E02002729 | 22 |  |  |
| E02002730 | 20 |  |  |
| E02002731 | 18 |  |  |
| E02002732 | 16 |  |  |
| E02002733 | 19 |  |  |
| E02002734 | 28 |  |  |
| E02002735 | 21 |  |  |
| E02002736 | 10 |  |  |
| E02002737 | 33 |  |  |
| E02002738 | 21 |  |  |
| E02002739 | 21 |  |  |
| E02002740 | 22 |  |  |
| E02002741 | 44 |  |  |
| E02002742 | 16 |  |  |
| E02002743 | 19 |  |  |
| E02002744 | 19 |  |  |
| E02002745 | 18 |  |  |
| E02002746 | 34 |  |  |
| E02002747 | 33 |  |  |
| E02002748 | 36 |  |  |
| E02002749 | 25 |  |  |
| E02002750 | 9 |  |  |
| E02002751 | 23 |  |  |
| E02002752 | 13 |  |  |
| E02002753 | 43 |  |  |
| E02002754 | 30 |  |  |
| E02002755 | 30 |  |  |
| E02002756 | 26 |  |  |
| E02002757 | 29 |  |  |
| E02002758 | 23 |  |  |
| E02002759 | 41 |  |  |
| E02002760 | 13 |  |  |
| E02002761 | 17 |  |  |
| E02002762 | 27 |  |  |
| E02002763 | 42 |  |  |
| E02002764 | 20 |  |  |
| E02002765 | 21 |  |  |
| E02002766 | 24 |  |  |
| E02002767 | 19 |  |  |
| E02002768 | 11 |  |  |
| E02002769 | 13 |  |  |
| E02002770 | 21 |  |  |
| E02002771 | 13 |  |  |
| E02003240 | 1 |  |  |
| E02003459 | 1 |  |  |
| E02003460 | 1 |  |  |
| E02003461 | 1 |  |  |
| E02003464 | 1 |  |  |
| E02003467 | 1 |  |  |
| E02003470 | 1 |  |  |
| E02005921 | 1 |  |  |
| E02005923 | 1 |  |  |
| E02005924 | 2 |  |  |
| E02005926 | 1 |  |  |
| E02006210 | 1 |  |  |
| E02006804 | 49 |  |  |
| E02006815 | 83 |  |  |
| E02006816 | 35 |  |  |
| E02006817 | 40 |  |  |
| E02006818 | 49 |  |  |

S-1b.

**Table S2.** MSOAs and cases excluded from cumulative period one (C1) from May 18^th^, 2020, to October 31^st^, 2020, including records from 2 MSOAs reported by the East Midlands Ambulance NHS Trust data (EMAS), and records from 5 MSOAs reported by GOV.UK.

| **EMAS** | | **GOV.UK** | |
| --- | --- | --- | --- |
| MSOA | Cases | MSOA | Cases |
| E02003460 | 1 | E02004093 | 104 |
| E02005924 | 1 | E02004094 | 172 |
|  |  | E02004095 | 192 |
|  |  | E02004096 | 203 |
|  |  | E02005851 | 57 |

**Table S3.** MSOAs and cases excluded from cumulative period one (C2) from May 18^th^, 2020, to April 17^th^, 2021, including records from 11 MSOAs reported by the East Midlands Ambulance NHS Trust data (EMAS), and records from 4 MSOAs reported by GOV.UK.

| **EMSA** | | **GOV.UK** | |
| --- | --- | --- | --- |
| MSOA | Cases | MSOA | Case |
| E02003240 | 1 | E02004093 | 337 |
| E02003459 | 1 | E02004094 | 465 |
| E02003460 | 1 | E02004095 | 523 |
| E02003461 | 1 | E02004096 | 541 |
| E02003464 | 1 |  |  |
| E02003467 | 1 |  |  |
| E02003470 | 1 |  |  |
| E02005923 | 1 |  |  |
| E02005924 | 2 |  |  |
| E02005926 | 1 |  |  |
| E02006210 | 1 |  |  |

**Table S4.** MSOAs and cases excluded from cumulative period one (C3) from May 18^th^, 2020, to October 2^nd^, 2021, including records from 11 MSOAs reported by the East Midlands Ambulance NHS Trust data (EMAS), and records from 4 MSOAs reported by GOV.UK.

| **EMSA** | | **GOV.UK** | |
| --- | --- | --- | --- |
| MSOA | Cases | MSOA | Case |
| E02003240 | 1 | E02004093 | 828 |
| E02003459 | 1 | E02004094 | 1122 |
| E02003460 | 1 | E02004095 | 1261 |
| E02003461 | 1 | E02004096 | 1220 |
| E02003464 | 1 |  |  |
| E02003467 | 1 |  |  |
| E02003470 | 1 |  |  |
| E02005923 | 1 |  |  |
| E02005924 | 2 |  |  |
| E02005926 | 1 |  |  |
| E02006210 | 1 |  |  |
